# Biosynthesis of Diverse Ephedra-Type Alkaloids via a Newly Identified Enzymatic Cascade

**DOI:** 10.34133/bdr.0048

**Published:** 2024-09-03

**Authors:** Peiling Wu, Ding Luo, Yuezhou Wang, Xiaoxu Shang, Binju Wang, Xianming Deng, Jifeng Yuan

**Affiliations:** ^1^ State Key Laboratory of Cellular Stress Biology, School of Life Sciences, Faculty of Medicine and Life Sciences, Xiamen University, Fujian 361102, China.; ^2^ College of Chemistry and Chemical Engineering, Xiamen University, Fujian 361105, China.; ^3^ Shenzhen Research Institute of Xiamen University, Shenzhen 518057, China.

## Abstract

Ephedra-type alkaloids represent a large class of natural and synthetic phenylpropanolamine molecules with great pharmaceutical values. However, the existing methods typically rely on chemical approaches to diversify the *N*-group modification of Ephedra-type alkaloids. Herein, we report a 2-step enzymatic assembly line for creating structurally diverse Ephedra-type alkaloids to replace the conventional chemical modification steps. We first identified a new carboligase from *Bacillus subtilis* (*Bs*AlsS, acetolactate synthase) as a robust catalyst to yield different phenylacetylcarbinol (PAC) analogs from diverse aromatic aldehydes with near 100% conversions. Subsequently, we screened imine reductases (IREDs) for the reductive amination of PAC analogs. It was found that IRG02 from *Streptomyces albidoflavus* had good activities with conversions ranging from 37% to 84% for the reductive alkylamination with diverse amine partners such as allylamine, propargylamine, and cyclopropylamine. Overall, 3 new bio-modifications at the *N*-group of Ephedra-type alkaloids were established. Taken together, our work lays a foundation for the future implementation of biocatalysis for synthesizing structurally diverse Ephedra-type alkaloids with potential new pharmaceutical applications.

## Introduction

Ephedra-type alkaloids are naturally produced by *Catha edulis* and members of the genus *Ephedra* [1,2]. This class of alkaloids run a gamut of pharmacologic activities such as the bronchodilator and adrenergic receptor agonists [3–5] for treating bronchial asthma and low blood pressure [6]. Plant extraction methods are commonly used to obtain natural Ephedra-type alkaloids. However, due to the limited applications of natural Ephedra-type alkaloid, there is current interest in producing synthetic Ephedra-type alkaloids with improved pharmacological properties and reduced side effects [7]. Researchers have created non-natural Ephedra-type alkaloids with diverse functionalization by chemical or biocatalytic approaches. The structural modifications on the phenyl ring and the *N*-group are commonly implemented to create synthetic Ephedra-type alkaloids with changed pharmacologic activities [8,9]. For instance, butaxamine with the *N*-group modification is used as a selective β2-adrenoceptor antagonist [10]. Methoxamine (a potential candidate as a vasopressor [11]) and metaraminol (the prevention and treatment of acute hypotension [12,13]) are 2 representative synthetic alkaloids with modifications at the phenyl ring.

In plants, (pseudo)ephedrines are synthesized from the precursor of 1-phenylpropane-1,2-dione by the condensation of the benzylic fragment with pyruvate [14], which is further modified by transamination and *N*-alkylation. Although it is important to elucidate the natural biosynthetic pathway for Ephedra alkaloids, the structural diversity from the natural pathway is only limited to a few types of compounds. Instead, synthetic Ephedra-type alkaloids were reported to provide sympathomimetic drugs and chemical synthons [15], and it is desirable to generate more structurally diverse Ephedra-type alkaloids with potential novel pharmaceutical activities. However, current efforts of using biological approaches mainly have focused on generating the diversity on the phenyl ring using different aromatic aldehydes as starting substrates [16,17]. There is plenty of scope for diversifying the *N*-group of Ephedra-type alkaloids [18].

**Fig. 1:**
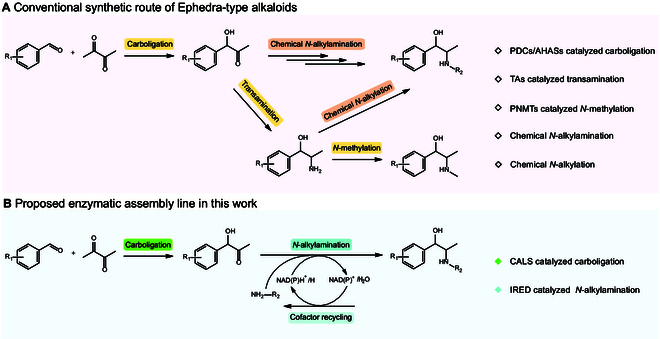
.Proposed 2-step enzymatic assembly line for creating diverse Ephedra-type alkaloids. (A) Conventional routes for synthesis of Ephedra-type alkaloids. (B) Two-step enzymatic assembly line of carboligation coupled with *N*-alkylamination to generate Ephedra-type alkaloids.

For the creation of different synthetic Ephedra-type alkaloids, chemical strategies involve the use of toxic reagents, long reaction procedures, and isolation [19–21], which are not environmentally friendly processes. In addition, different group modifications require distinct synthetic routes, which would limit its diversified synthesis [22,23]. Biological approaches have been developed to synthesize synthetic and natural Ephedra-type alkaloids. The early biocatalytic methods focused on modifying the phenyl ring to produce different nor(pseudo)ephedrine analogs (Fig. 1A). For instance, Rother et al. reported the synthesis of 4 nor(pseudo)ephedrine isomers by combining carboligases such as pyruvate decarboxylases (PDCs) [24] and acetohydroxyacid synthases (AHASs) [25] and transaminases (TAs) with different enantioselectivities [17,26]. Based on the promiscuity of these enzymes, these enzymatic cascades were further expanded to the synthesis of methoxamine [11] and metaraminol [13]. For the *N*-functionalization, *S*-adenosylmethionine (SAM)-dependent methyltransferases (PNMTs) [27] have been reported to catalyze the *N*-methylation of nor(pseudo)ephedrine. However, other *N*-modifications of nor(pseudo)ephedrine have to rely on chemical approaches (Fig. 1A), which require the use of toxic metal catalysts and large amounts of organic solvents [28].

To overcome the limitations of phenyl-ring modification and *N*-functionalization of non-natural Ephedra-type alkaloids, we aimed to develop a more concise 2-step enzymatic assembly line to create diverse Ephedra-type alkaloids, which eliminates toxic chemical approaches (Fig. 1B). We first identified that acetolactate synthase from *Bacillus subtilis* (*Bs*AlsS) exhibited a superior substrate scope for the carboligation of diverse aromatic aldehydes with pyruvate. To address the limitation of biological *N*-alkylamination, we screened and identified an imine reductase (IRED) from *Streptomyces albidoflavus* for one-step reductive alkylamination with different amine donors. Overall, we successfully synthesized 16 phenylacetylcarbinol (PAC)-class α-hydroxyketones with good yields and demonstrated 3 new bio-modifications at the *N*-group of Ephedra-type alkaloids. Taken together, we successfully explored the biological approach to expand the structural diversity of synthetic Ephedra-type alkaloids, which will provide new drug candidates with potential pharmaceutical applications.

## Materials and Methods

### Strains and reagents

*Escherichia coli* strain DH5α was used for plasmid constructions. *E. coli* MG1655 RARE [29] was used for whole-cell biotransformation studies. Luria–Bertani (LB) medium (1% tryptone, 0.5% yeast extract, 1% NaCl) with appropriate antibiotics (100 μg/ml ampicillin, 34 μg/ml chloramphenicol, and 50 μg/ml streptomycin) was used for the routine cultivation of *E. coli*. All restriction enzymes, T4 ligase, and Phusion High-Fidelity DNA polymerase were purchased from New England Biolabs (Beverly, MA, USA). The gel extraction kit and the plasmid purification kit were purchased from BioFlux (Shanghai, China). All chemicals, reagents, and resources used in this study are listed in Table S1.

### Cloning and strain construction

Empty vector pRSFDuet-1, pCDFuet-1, and pET28a were purchased from Novagen. Gene *Ec*ilvB together with *Ec*ilvN were amplified from the genomic DNA of *E. coli* strain MG1655. Gene *Bs*alsS was amplified from the genomic DNA of *B. subtilis* 168*.* The polymerase chain reaction (PCR) fragments were cloned into pRSFDuet-1 vector at *Bam*HI and *Xho*I restriction sites to construct plasmid pRSF-*Ec*ilvBN and pRSF-*Bs*alsS. Plasmid pET28a-*Asp*RedAm^Q240A^ and pET28a-IR77^A208N^ were synthesized by GenScript (Nanjing, Jiangsu, China). Plasmid pET28a-IRG02 and pET21b-*Bm*GDH [30] are gifts from S.-S. Gao, Chinse Academy of Sciences. Glucose dehydrogenase from *Bacillus megaterium* (*Bm*GDH) was cloned into pCDFDuet-1 vector to yield pCDF-*Bm*GDH. All primers used in this study are listed in Table S2. Plasmids used in this study are listed in Table S3, and all strains are listed in Table S4.

### Protein expression and purification

Protein expression procedures: 1% fresh overnight culture was inoculated into 100 ml of Terrific broth (2.4% yeast extract, 1.2% tryptone, 1.25% K_2_HPO_4_, 0.23% KH_2_PO_4_, 4% glycerol) with corresponding antibiotics. The *E. coli* culture was first incubated at 37 °C and 250 rpm. When the optical density of cell culture reached 0.8 to 1.0, isopropyl β-d-1-thiogalactopyranoside (IPTG) was added to a final concentration of 0.4 mM, and the cell culture was further cultivated at 20 °C and 250 rpm for 16 to 18 h to induce the expression of desired proteins. The optical density of cell culture was measured by BioTek Synergy H1 Reader (BioTek, USA).

Protein purified procedure: *E. coli* cells were harvested at 7,000 rpm for 5 min and the pellets were resuspended in the buffer containing 1.25% K_2_HPO_4_, 0.23% KH_2_PO_4_, 2.93% NaCl, 0.07% imidazole, and 1 mM phenylmethylsulfonyl fluoride. The cell lysis was performed by ultrasonication (3 s ON, 3 s OFF, 70 cycles), and the supernatant was collected by centrifugation at 4 °C, 10,000 rpm for 10 min. After filtering the supernatant by 0.45-μm filter, the cell lysate was loaded into gravity flow columns charged with 5 ml of Ni-NTA agarose and eluted by the buffer containing 500 mM NaCl and 20 to 200 mM imidazole. The concentration of purified protein was determined by the Bradford protein assay kit. The purity of proteins was analyzed by sodium dodecyl sulfate–polyacrylamide gel electrophoresis (SDS-PAGE). Further removing salt ions and imidazole was carried out by dialysis.

### Biotransformation procedure

For the whole-cell biocatalyst, cells were harvested at a speed of 7,000 rpm for 5 min. Cell pellets were washed once with distilled water and resuspended to a final concentration of 30 or 60 g/l cell dry weight (CDW) in potassium phosphate (KP) buffer (pH 7.0, 100 mM). Biotransformation was conducted with either whole-cell catalysts or purified enzymes. The whole-cell biotransformation was typically performed in a 1-ml reaction mixture, the purified enzyme-catalyzed reaction was performed in a 0.5-ml reaction mixture, and the scale-up preparation was performed in a 30-ml reaction mixture.

For the carboligase-mediated condensation reaction, 1 ml of reaction mixture consists of 5 to 30 mM aromatic aldehyde, 10 to 60 mM pyruvate (the ratio of aldehyde to pyruvate was kept at 1:2), 20 g/l d-glucose, 10 g/l CDW whole-cell biocatalysts, 0.5 mM dithiothreitol (DTT), 60 mM KCl, 5 mM MgCl_2_·6H_2_O, and 0.1 mM thiamine pyrophosphate (ThDP), in 1 ml of KP buffer (pH 7.0, 100 mM). Reactions were incubated at 30 °C with a shaking speed of 250 rpm for 24 h. For purified enzyme-mediated reaction, a 0.5-ml reaction mixture was prepared in a similar way with an equivalent of enzyme. For the scale-up preparation of α-hydroxyketones, 30 ml of reaction mixtures was prepared in a similar way to 1 ml of the reaction mixture as mentioned above with 20 mM aromatic aldehyde and 40 mM pyruvate. Substrates **a** and **b** were performed with 30 mM, and substrates **h**, **j**, **k**, and **p** were performed with 10 mM.

### IRED-mediated *N*-alkylamination of α-hydroxyketone

PAC (**1a**) was used as the model substrate to screen suitable IREDs. The biotransformation contains 20 g/l d-glucose, 5 mM PAC, the corresponding amines donors, and 20 g/l CDW whole-cell biocatalysts coexpressing IRED and *Bm*GDH, in 1 ml of KP buffer (pH 7.0, 100 mM). Reaction mixtures were incubated at 30 °C with a shaking speed of 250 rpm for 24 h. For IRG02-catalyzed reductive *N*-alkylamination of PAC analogs, the reaction mixture contains 5 mM PAC-class α-hydroxyketones, 20 g/l d-glucose, 20 g/l CDW whole-cell catalysts with IRG02 and *Bm*GDH, and the appropriate ratio of amine donors, in 1 ml of KP buffer (pH 7.0, 100 mM). The reaction mixtures were incubated at 30 °C with a shaking speed of 250 rpm for 24 h.

### One-pot biocatalysis procedure

For the one-pot concurrent synthesis of Ephedra-type alkaloids, the reaction mixture comprises 5 to 30 mM aldehyde, 10 to 60 mM pyruvate, the appropriate ratio of amine donors, 20 g/l d-glucose, 20 g/l CDW whole-cell catalysts (expressing *Bs*AlsS, IRG02, and *Bm*GDH), 0.5 mM DTT, 60 mM KCl, 5 mM MgCl_2_·6H_2_O, and 0.1 mM ThDP, in 1 ml of KP buffer (pH 7.0, 100 mM). The reaction mixtures were incubated at 30 °C with a shaking speed of 250 rpm for 24 h. For the one-pot sequential step, the reaction mixtures were carried out in a similar way to the one-pot concurrent step without the supplementation of amine donors. The reaction mixtures were incubated at 30 °C with a shaking speed of 250 rpm for 4 h to perform the *Bs*AlsS-mediated carboligation. Subsequently, an appropriate ratio of amine donor was added to make up to 1 ml of reaction volume, and the IRED-mediated reductive *N*-alkylamination was continued for another 20 h.

### HPLC and MS/MS analysis

For product analysis, 50 μl of the sample was diluted with appropriate amounts of 10% acetonitrile (ACN) solution and centrifuged at 14,000 rpm for 10 min to remove the cell pellet. The supernatant was analyzed by high-performance liquid chromatography (HPLC). HPLC analysis was performed using Shimadzu Prominence LC-20AD system with a C18 reversed-phase column (Shim-pack GIST C18-AQ, 5 μm, 4.6 × 150 mm). Different ratios of ACN and ultrapure water with 0.1% trifluoroacetic acid (TFA) were used as the mobile phase. The flow rate was set at 1 ml/min, and the column temperature was maintained at 40 °C. Both standards and samples were determined using the detection wavelength at 210 nm. Tandem mass spectrometry (MS/MS) analysis was performed on the TripleTOF 5600+ System. Ions with an intensity higher than 2.0 × 10^4^ counts were fragmented by MS/MS fragmentation with the resolution set to 60,000, AGC (automatic gain control) target to 3.0 × 10^4^, maximum injection time to 50 ms, and HCD (higher-energy C trap dissociation) collision energy (%) to 30. Ions were excluded from further fragmentation if they occurred more than once in 60 s.

### Docking analysis

Docking procedures are executed using GNINA software. The AB chains of *Bs*AlsS, the AC chains of *Ec*IlvBN, and the AB chains of 2VJY are selected for superposition to identify the reactive pocket. Only one pocket is retained for downstream analyses. ThDP within the AB chains of *Bs*AlsS and the AC chains of *Ec*IlvBN is manually modified to hydroxyethylThDP (HEThDP). The coordinates of pyruvate near the ThDP of the AC chain in 2VJY serve as a reference point for the ligand 4-hydroxybenzaldehyde, and a box with a 4-Å radius around pyruvate is utilized for docking. Each docking iteration produces 100 poses, with an exhaustive sampling value set to 50. Poses are selected based on a combination of high-scoring ranks and appropriate reaction distances for binding conformations. For the docking results of *Bs*AlsS with 4-hydroxybenzaldehyde, the pose with the second-highest convolutional neural networks (CNN) score is chosen for analysis (reaction distance is 3.5 Å, CNN pose score = 0.9094, CNN affinity = 3.690). For the docking results of *Ec*IlvBN with 4-hydroxybenzaldehyde, the pose with the highest CNN score is selected for analysis (reaction distance is 4.2 Å, CNN pose score = 0.8442, CNN affinity = 3.985). Interactions are visualized using PyMOL software.

## Results and Discussion

### Identification of acetolactate synthase from *B. subtilis* for PAC production

Carboligation of substituted aromatic aldehydes and pyruvate generates α-hydroxyketones, which serve as intermediates to produce natural and synthetic Ephedra-type alkaloids. To increase the diversity at the phenyl ring, the pivotal goal is to identify a highly active carboligase with a broad substrate scope. The family of ThDP-dependent lyases catalyzes a broad range of C–C formation and cleavage reactions. PDCs and AHASs have been extensively explored for biomanufacturing PAC and its analogs [25,31–33]. Compared to the PDC-mediated carboligation that results in acetaldehyde as a by-product, AHASs have a clear advantage in their intrinsic carboligation without such by-product [25]. To date, *Ec*IlvBN from *E. coli* that contains a large subunit IlvB and a small regulatory subunit IlvN represents one of the most commonly used AHASs for PAC production [11,13]. Considering that *Bs*AlsS has been extensively applied to the production of branched-chain amino acid-derived chemicals including isobutanol [34,35], it is likely that *Bs*AlsS might have the carboligation activity for condensing benzaldehyde and pyruvate to generate L-PAC. However, catabolic acetolactate synthase (CALS) [36,37] such as *Bs*AlsS from *B. subtilis* is rarely studied for PAC production.

**Fig. 2.**
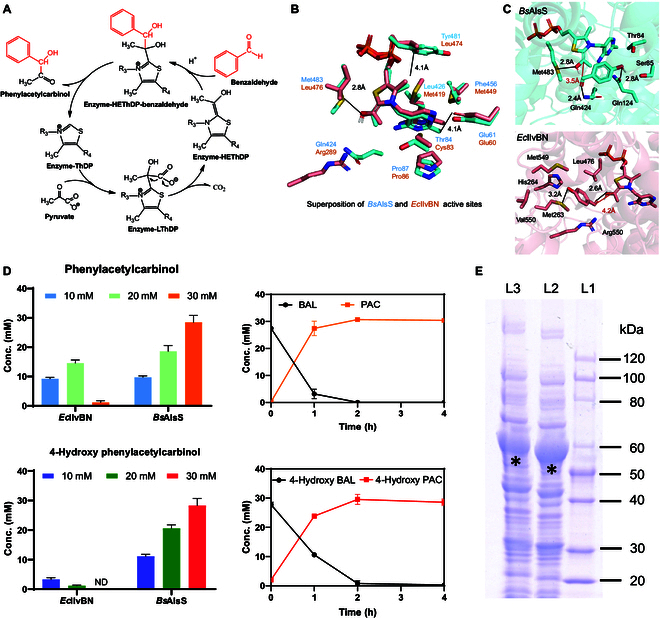
Identification of *Bs*AlsS as an effective carboligase for the synthesis of PAC-class α-hydroxyketones. (A) Common mechanism of the ThDP-dependent enzymatic reaction. (B) Superimposed active pockets of *Bs*AlsS and *Ec*IlvBN. Cyan represents *Bs*AlsS (PDB ID: 4RJJ); bright pink represents *Ec*IlvBN (PDB ID: 6LPI). (C) Docking results of *Bs*AlsS (top panel) and *Ec*IlvBN (bottom panel) with the substrate 4-hydroxybenzaldehyde. (D) Experimental validation of *Bs*AlsS-mediated carboligation for the synthesis of PAC and 4-hydroxy PAC. The left panel shows the titer under different substrate concentrations, and the right panel indicates time courses for the conversion of 30 mM substrates using *Bs*AlsS. Error bars indicate standard deviations from triplicate experiments. (E) SDS-PAGE analysis of biocatalysts (MR-*Bs*AlsS and MR-*Ec*IlvB).

As depicted in Fig. 2A, ThDP-dependent carboligases typically share the same catalytic cycle for the condensation of aromatic aldehydes with pyruvate [38,39]. The bound ThDP anion reacts with pyruvate to form lactylThDP (LThDP), which undergoes decarboxylation to form HEThDP. In the presence of benzaldehyde, the HEThDP intermediate can react with benzaldehyde to form a bound arylacetyl carbinol, which is further released as PAC. In this study, molecular docking was first applied to compare the active sites of *Bs*AlsS [Protein Data Bank (PDB) ID: 4RJJ] with *Ec*IlvBN (PDB ID: 6LPI). Notably, both *Bs*AlsS and the catalytic unit of *Ec*IlvBN share a similar substrate binding pocket (Fig. 2B), suggesting the potential implementability of *Bs*AlsS for condensing benzaldehyde with pyruvate. Additionally, we performed molecular docking simulations with another bulky substrate of 4-hydroxybenzaldehyde. As shown in Fig. 2C, aromatic rings of residues Tyr^481^ and Phe^456^ in *Bs*AlsS are likely to guide the substrate HEThDP into a more stable position. The Met residue at position 483 from *Bs*AlsS is smaller, conferring greater substrate tolerance. More interestingly, the residue Met^483^ in *Bs*AlsS may act as a hydrogen bond acceptor, forming bonds with the OH group on the substrate. These robust interactions may allow 4-hydroxybenzaldehyde to bind near the substrate HEThDP at an optimal reaction distance of 3.5 Å (Fig. 2C). In comparison, Leu^476^ in *Ec*IlvBN is a larger amino acid than Met^483^ in *Bs*AlsS, and this size difference can lead to steric hindrance, potentially affecting the binding of bulky aromatic substrates.

To experimentally validate the activity of *Bs*AlsS, both benzaldehyde (BAL; **a**) and 4-hydroxy benzaldehyde (4-HBAL; **b**) were tested as the substrates. As shown in Fig. 2D, the holoenzyme *Ec*IlvBN converted 20 mM BAL **a** to 13 mM PAC **1a** (~65% conversion). 4-HBAL **b** (10 mM) gave 8 mM 4-hydroxy PAC **1b** by *Ec*IlvBN, but the activity of *Ec*IlvBN was completely abolished at 20 mM 4-HBAL **b**. Encouragingly, *Bs*AlsS exhibited a high catalytic activity toward substrates **a** and **b**, and both **a** and **b** were fully converted to products **1a** and **1b** within 4 h. As *Bs*AlsS is a FAD-independent enzyme, we noticed that the cell pellet of *Bs*AlsS-expressing *E. coli* gave a different color when compared to that of *Ec*IlvBN-expressing *E. coli* (Fig. S1). Further validation of the whole-cell biocatalysts by SDS-PAGE confirmed the successful overexpression of MR-*Bs*AlsS and MR-*Ec*IlvBN (Fig. 2E). Furthermore, we conducted a comparative analysis of the biocatalytic efficiency between the whole-cell biocatalyst and the purified *Bs*AlsS. The results revealed that the whole-cell biocatalyst exhibited a comparable activity to that of the purified enzyme (Fig. S3). For substrate **a**, we observed a slightly higher biocatalytic efficiency of the whole-cell biocatalyst compared to that of the purified *Bs*AlsS. However, a lower conversion of substrate **b** in whole-cell biocatalysis compared to that of the purified *Bs*AlsS was observed (Fig. S3). We reasoned that substrate **b** might exhibit a reduced permeability across the cell membrane barrier in the whole-cell biocatalysis due to the presence of an additional hydroxy group. Nevertheless, considering that whole-cell biocatalysis could substantially reduce the operating cost when compared to purified enzymes, it is more feasible for the future scale-up of bioproduction processes.

### Broad substrate scope of *Bs*AlsS for synthesis of diverse α-hydroxyketones

**Fig. 3.**
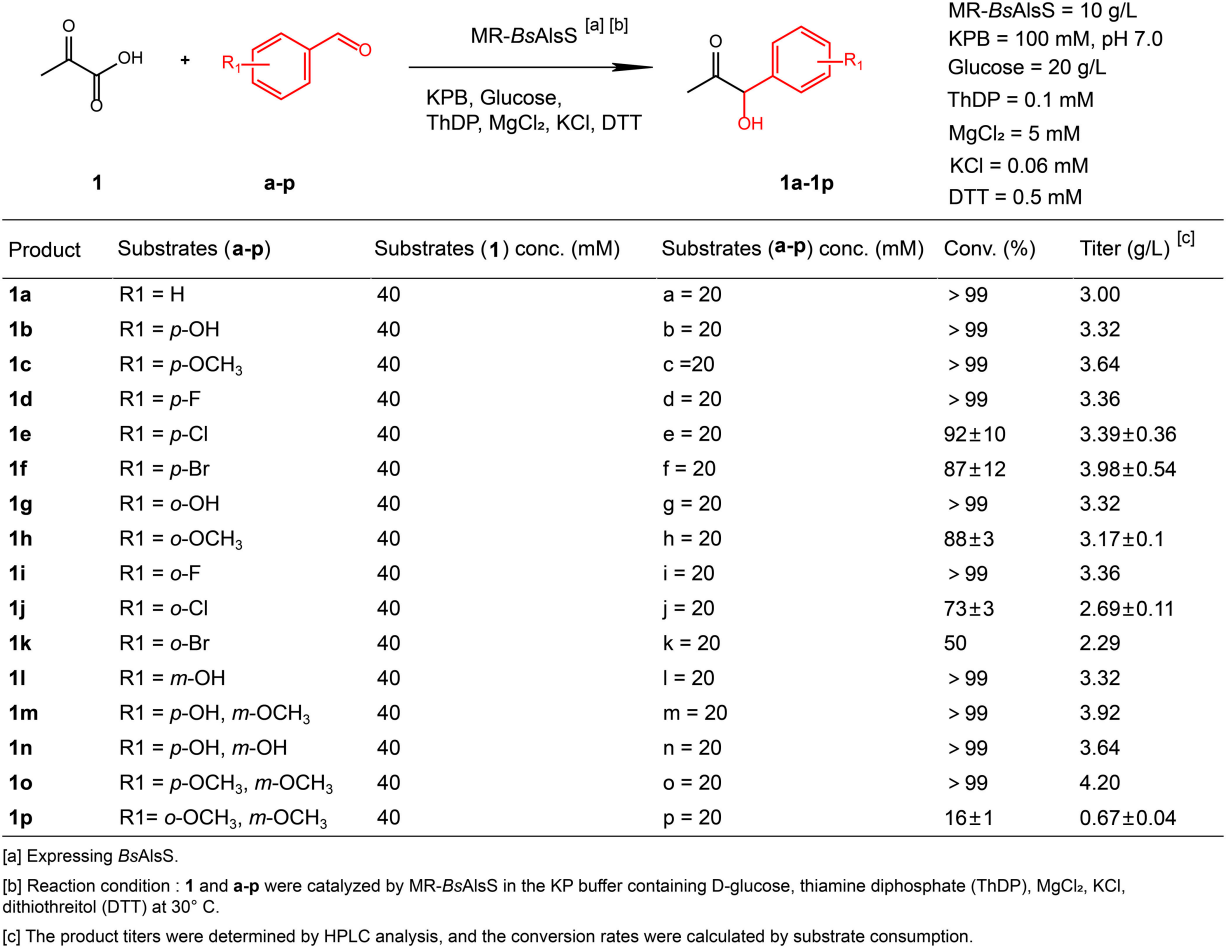
The summary of phenylacetylcarbinol (PAC) and its analogs.

After obtaining the encouraging results from *Bs*AlsS, we attempted to further assess its catalytic activity on various substituted aromatic aldehydes. As listed in Fig. 3, a set of substrates **c** to **p** with different substitutions of hydroxy, methoxy, and halogen at *ortho*, *meta*, and *para* positions of the phenyl ring were selected. All reactions proceeded with ~100% conversions under 5 mM **c** to **p** (data not shown). Even at the concentration of 20 mM **c** to **p**, the majority of products **1c** to **1p** were obtained at good yields, as calculated by liquid chromatography analysis (Fig. S4) and structurally validated by mass spectrum analysis (Fig. S12). For instance, substrates **c**, **d**, **g**, **i**, **l**, **m**, **n**, and **o** afforded products **1c**, **1d**, **1g**, **1i**, **1l**, **1m**, **1n**, and **1o** with excellent >99% conversions. Substrates **e**, **f**, **h**, **k**, and **j** gave products **1e**, **1f**, **1h**, **1k**, and **1j** with efficiencies ranging from 50 to 92%. Substrate **p** was poorly transformed when the concentration was over 10 mM, and 20 mM substrate **p** only gave **1p** at 16% conversion, indicating that the combined *ortho*- and *meta*-methoxy functionalization might hinder the substrate binding to *Bs*AlsS.

Some trends could be observed from the conversion profile: aromatic aldehydes substituted with hydroxyl group in the *ortho*, *meta*, and *para* position (**1b**, **1g**, **1l**) provided high conversions; halogen modifications showed that fluorine-substituted substrates (**1d**, **1i** ) are more active than those with chlorine and bromine modifications (**1e**, **1f, 1j**, **1k**), probably because the smaller size of side group [40,41] can make the substrates easily interact with the active site of *Bs*AlsS. The *para* functionalization provided higher conversions than the *ortho* modification (**1b** to **1f** versus **1g** to **1k**). Excellent conversions were achieved by combining *para*- with *ortho*-modifications (2 hydroxyls **1n**, 2 methoxys **1o**, or hydroxyl with methoxy **1p**). Overall, *Bs*AlsS exhibited relatively good activities in generating a series of PAC class α-hydroxyketones with modifications of halogen, hydroxyl, and methoxy groups. Therefore, *Bs*AlsS could serve as a robust biocatalyst for providing diverse substituted α-hydroxyketones, which can be further functionalized into synthetic Ephedra-type alkaloids.

### Screening IREDs for reductive *N*-alkylamination of α-hydroxyketones

**Fig. 4:**
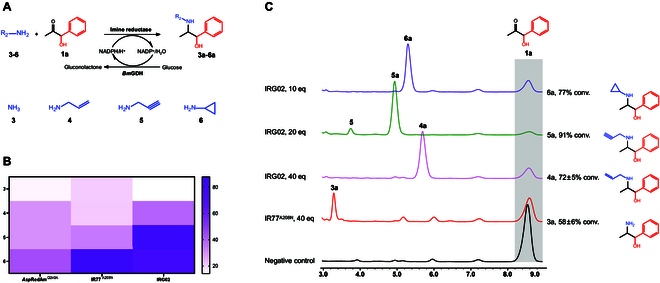
.Screening IREDs for effective reductive amination of PAC to Ephedra-type alkaloids. (A) Schematic diagram of reductive amination of PAC with amine donors 3 to 6. Substrate 1a was tested with ammonia 3, allylamine 4, propargylamine 5, and cyclopropylamine 6. (B) Heatmap of conversions of different IREDs. Three IREDs, including *Asp*RedAm^Q240A^, IR77^A208N^, and IRG02, were investigated. (C) Representative liquid chromatography results and conversions of IRED-mediated formation of natural and synthetic Ephedra-type alkaloids 3a to 6a. Error bars indicate standard deviations from triplicate experiments.

To date, the biological method for *N*-alkylation is limited to SAM-dependent PNMTs [27]. Chemical approaches are required to functionalize the *N*-group of nor(pseudo)ephedrine with other *N*-alkylations besides *N*-methylation. Recently, asymmetric *N*-alkylation of ketones has gained great interest by using IREDs [42,43]. The implementation of IREDs for the reductive *N*-alkylamination of α-hydroxyketones simplifies the traditional TA-mediated transamination followed by chemical modifications into a single-step enzymatic reaction (Fig. 4A). The diversity of *N*-alkylation products generated by IREDs can be easily adjusted by altering amine partners.

In this study, a set of IREDs was selected for testing: AspRedAm^Q240A^ from *Aspergillus oryzae* was chosen for its broad-scope carbonyl compounds with a variety of primary and secondary amines [44]; IR77^A208N^ from *Ensifer adhaerens* [45] and IRG02 from *S. albidoflavus* [30] were selected because of their excellent capacities for bulky substrates. The relative activity of IREDs toward the representative PAC (**1a**) was determined using a set of amine partners (**3** to **6**). As shown in Fig. S5, we confirmed the successful expression of IRED together with *Bm*GDH in *E. coli*. Based on the activity chart of different IREDs (Fig. 4B), IRG02 exhibited excellent activity toward amine donors **4** to **6**: Substrate **1a** was converted to secondary amine products **4a** to **6a** at 37%, 84%, and 77%. IRG02 showed higher specific activities with propargylamine **5** and cyclopropylamine **6** over that of ammonia **3** and allylamine **4**. However, we found that IR77^A208N^ exhibited a higher activity with ammonia **3** when compared to that of IRG02. In comparison, AspRedAm^Q240A^ was less reactive toward tested amine partners **3** to **6** (Figs. S7 to S10). The assay revealed that IREDs such as IRG02 and IR77^A208N^ would facilitate the reductive *N*-alkylamination of PAC with good conversions. Three new bio-modifications (allyl **4a**, propargyl **5a**, and cyclopropyl **6a**) at the *N*-group were established for the first time for creating synthetic Ephedra-type alkaloids, which were structurally confirmed by mass spectrum analysis (Fig. S12).

To further improve the reductive *N*-alkylamination of PAC, we attempted to optimize the ratio of amine equivalents for improved conversions. As shown in Fig. 4C, different conversions by IRG02 were achieved: Substrates **1a** and **3** (40 equivalents) gave product **3a** with 58% conversion; substrates **1a** and **4** (40 equivalents) gave product **4a** with 72% conversion; substrates **1a** and **5** (20 equivalents) gave product **5a** with 91% conversion; substrates **1a** and **6** (10 equivalents) delivered product **6a** with 77% conversion. In addition, IR77^A208N^-mediated reductive amination achieved 58% conversion for the synthesis of product **3a**.

**Fig. 5.**
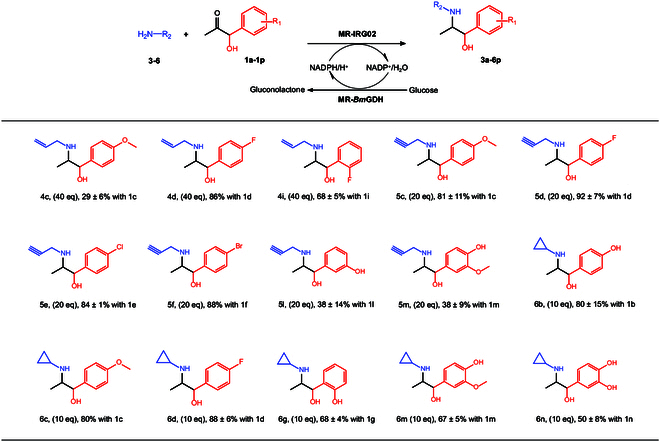
Expanding the *N*-functionalization on diverse PAC analogs. The whole-cell catalyst of MR-IRG02-*Bm*GDH was used to diversify Ephedra-type alkaloids with different PAC analogs and amine donors. The scope of amine donors: allylamine 3, propargylamine 4, and cyclopropylamine 5. The scope of PAC analogs was randomly chosen from 1a to 1p. The conversions were roughly estimated by substrate consumption. Error bars indicate standard deviations from triplicate experiments.

Based on the PAC-mediated reductive amination protocol, a series of biotransformation was performed for transferring PAC-class α-hydroxyketones (**1b** to **1p**) with different amine partners (**4** to **6**) using IRG02. When allylamine **4** was used as an amine partner, IRG02 showed good activities toward **1c**, **1d**, and **1i** and generated alkenyl-containing derivatives **4c**, **4d**, and **4i** with 28 to 86% conversions (Fig. 5 and Fig. S11). Propargylamine **5** afforded products **5c** to **5f**, **5l**, and **5m** with 38 to 92% conversions (Fig. 5 and Fig. S11). Substrate **6** gave products **6b**, **6c**, **6d**, **6g**, **6m**, and **6n** with 50 to 88% conversions (Fig. 5 and Fig. S11). In addition, representative synthetic Ephedra-type alkaloids were also confirmed by mass spectrum analysis (Fig. S12). Overall, 18 synthetic Ephedra-type alkaloids were successfully produced, and there is still plenty of room for future exploration of new combinations with different α-hydroxyketones and amine partners.

### One-pot biocatalytic synthesis of Ephedra-type alkaloids

**Fig. 6.**
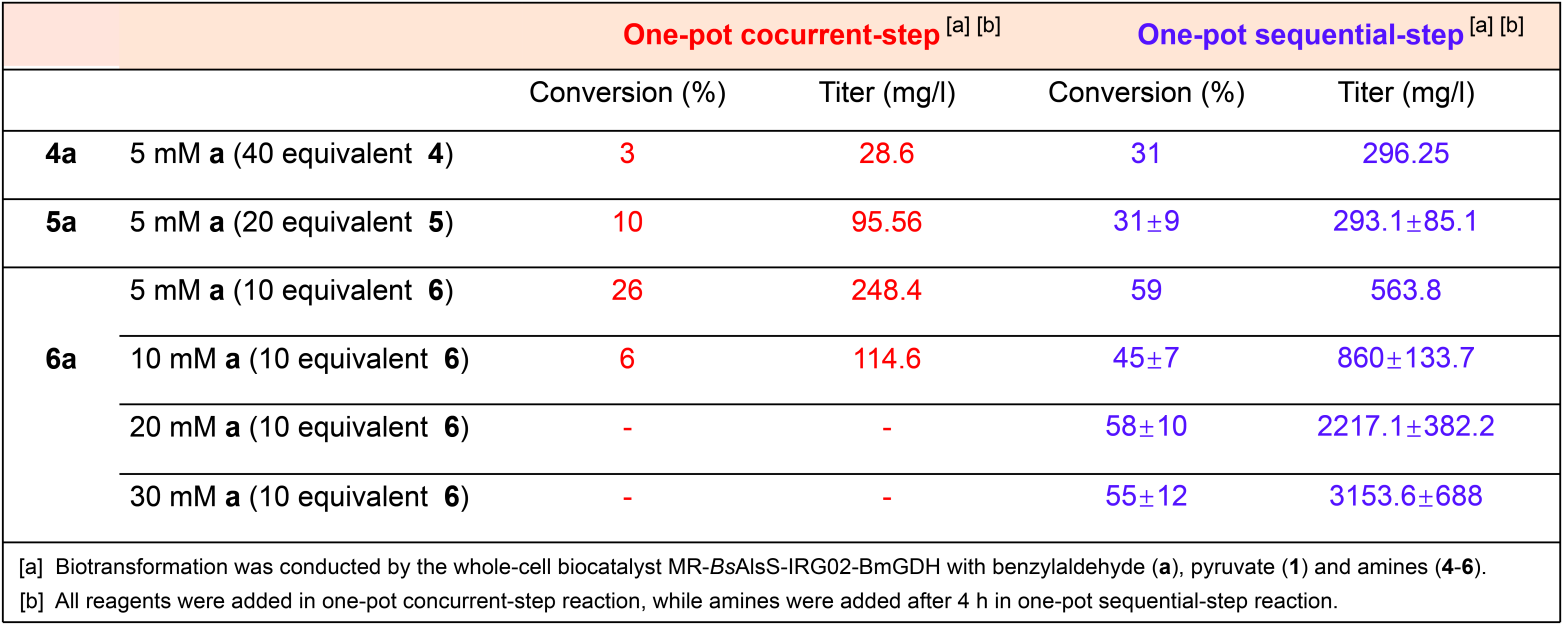
The summary of products **4a** to **6a** under different catalytic manners.

Upon confirming the substrate scope of *Bs*AlsS and IRG02, we next attempted to explore the one-pot cascaded biocatalysis for the synthesis of Ephedra-type alkaloids using a 2-step enzymatic assembly line. As shown from the SDS-PAGE result (Fig. S6.1), we successfully expressed *Bs*AlsS, IRG02, and *Bm*GDH in a single *E. coli*. However, during the one-pot reaction, IRG02 exhibited a clear preference for the reductive amination of aromatic aldehydes over α-hydroxyketones (Fig. S6.2), favoring the formation of by-products. One-pot concurrent conversion of BAL **a**, pyruvate **1**, and cyclopropylamine **6** resulted in approximately 74% of the substrate **a** into a by-product, whereas the target product **6a** was only obtained at 26 ± 1% (Fig. 6). Subsequently, the one-pot sequential strategy was introduced to temporally separate 2 enzymatic reactions: Amine donors were added after the completion of *Bs*AlsS-mediated carboligation. As shown in Table 2, 5 mM **a** gave 2.95 mM product **6a**, reaching approximately 59% conversion. Additionally, we also expanded the one-pot sequential reaction for the synthesis of **4a** and **5a**, both of which reached ~31% conversion.

## Conclusion

To replace the traditional TA-mediated transamination followed by chemical *N*-alkylamination, we adopted the IRED-mediated reductive *N*-alkylamination for the one-step functionalization of PAC. IRG02 from *S. albidoflavus* was identified as a good candidate for the reductive alkylamination of diverse α-hydroxyketones with different amine partners. Overall, 18 non-natural Ephedra-type alkaloids were generated by IRG02, and some of these new-to-nature compounds were first-time fabricated by biological approaches, which might serve as potential candidates for sympathomimetic drugs [18]. Notably, the *N*-alkylamination by propargylamine (**5**) is of particular interest to chemists, as further diversity at the alkynyl group could be easily created by using click chemistry [46]. In addition, these new-to-nature compounds might be used for the synthesis of synthetic tetrahydroisoquinoline (THIQ) [47] for improved antitumor, antiparasitic, and neurological activities. In the future, the structural diversity of synthetic Ephedra-type alkaloids will be further expanded by engineered CALSs and IREDs with better activities [48,49]. In summary, we envision that more synthetic Ephedra-type alkaloids could be created through the integration of knowledge and tools from chemistry and biology [50,51].

## Supplementary Materials

Supplementary 1 Figs. S1 to S12 Tables S1 to S4 References
