# Supplementary material for: Biosynthesis of Diverse Ephedra-Type Alkaloids via a Newly Identified Enzymatic Cascade
Source: Biodes Res. 2024 Sep 3;6:0048. doi: 10.34133/bdr.0048 (PMC11371322; doi:10.34133/bdr.0048)
Supplement: Supplementary 1 — Figs. S1 to S12 Tables S1 to S4 References [file bdr.0048.f1.docx]

**Supplementary material**

Biosynthesis of diverse Ephedra-type alkaloids via a newly identified enzymatic cascade

Peiling Wu^a^, Ding Luo ^b^, Yuezhou Wang ^a^, Xiaoxu Shang ^a^, Binju Wang ^b^, Xianming Deng ^a^, Jifeng Yuan*^ac^

^a^ State Key Laboratory of Cellular Stress Biology, School of Life Sciences, Faculty of Medicine and Life Sciences, Xiamen University, Fujian 361102, China

^b^ College of Chemistry and Chemical Engineering, Xiamen University, Fujian 361005, China

^c^ Shenzhen Research Institute of Xiamen University, Shenzhen 518057, China

* Corresponding author Email address: [jfyuan@xmu.edu.cn](mailto:jfyuan@xmu.edu.cn)

**Section 1 List of Supplementary Tables**

| **Table S1. List of equipment, reagents and resources.** | | |
| --- | --- | --- |
| Equipment | | Resource |
| Shimadzu Prominence LC-20AD liquid chromatography system | | Shimadzu |
| UV detector | | Shimadzu |
| C18 reversed phase column | | Shim-pack GIST C18-AQ, 5μm, 4.6x150mm |
| TripleTOF 5600 mass spectrometer | | SCIEX |
| BioTek Synergy H1 microplate reader | | BioTek |
| Software | | Resource |
| LabSolutions | | Shimadzu |
| SCIEX OS software | | SCIEX |
| Analyst TF software | | SCIEX |
| Microsoft PowerPoint | | Microsoft |
| Snapgene | | https://www.snapgene.com/ |
| GraphPad Prism | | https://www.graphpad.com/ |
| Adobe Illustrator 2020 | | Adobe Systems |
| Reagent | | Source |
| High-Fidelity Phusion DNA polymerase | | New England Biolabs |
| *Bam*HI-HF | | New England Biolabs |
| *Xho*I | | New England Biolabs |
| *Nde*I | | New England Biolabs |
| T4 DNA ligase | | New England Biolabs |
| Agarose | | Sigma-Aldrich |
| Gel extraction kit | | BioFlux |
| PCR purification kit | | BioFlux |
| DNA extraction kit | | BioFlux |
| Tryptone | | Sangon Biotech |
| Yeast extract | | Sangon Biotech |
| Kanamycin | | INALCO |
| Ampicillin | | INALCO |
| Streptomycin | | INALCO |
| Phenylmethylsulfonyl fluorid (PMSF) | | Sangon Biotech |
| Imidazole | | Solarbio |
| ProteinRuler^®^ II | | TransGen Biotech |
| Bradford Protein Assay Kit | | Beyotime |
| Protein gels | | ACE biotechnology |
| TureColor Pre-stained Protein Marker | | Sangon Biotech |
| Ni-NTA Agarose | | Sangon Biotech |
| Protein dialysis bag | | Biosharp |
| HPLC-grade acetonitrile (ACN) | | ANPEL Laboratory Technologies |
| Benzaldehyde (**a**) | | Aladdin |
| 4-Hydroxybenzaldehyde (**b**) | | Aladdin |
| 4-Methoxybenzaldehyde (**c**) | | Aladdin |
| 4-Fluorobenzaldehyde (**d**) | | Aladdin |
| 4-Chlorobenzaldehyde (**e**) | | Aladdin |
| 4-Bromobenzaldehyde (**f**) | | Aladdin |
| Salicylaldehyde (**g**) | | Aladdin |
| 2-Methoxybenzaldehyde (**h**) | | Aladdin |
| 2-Fluorobenzaldehyde (**i**) | | Aladdin |
| 2-Chlorobenzaldehyde (**j**) | | Aladdin |
| 2-Bromobenzaldehyde (**k**) | | Aladdin |
| 3-Hydroxybenzaldehyde (**l**) | | Aladdin |
| Vanillin (**m**) | | Aladdin |
| 3,4-Dihydroxybenzaldehyde (**n**) | | Aladdin |
| 3,4-Dimethoxybenzaldehyde (**o**) | | Aladdin |
| 2,5-Dimethoxybenzaldehyde (**p**) | | Aladdin |
| Ammonia (**3**) | Macklin | |
| Allylamine hydrochloride (**4**) | Macklin | |
| Propargylamine (**5**) | Macklin | |
| Cyclopropylamine (**6**) | Macklin | |
| L-Phenylacetyl carbinol (L-PAC) | Toronto Research Chemicals | |

**Table S2. List of primer used in this study.**

| Primers | Sequence |
| --- | --- |
| IlvBN_BsaI_fwd1 | TTGGTCTCGGATCCGATGGCAAGTTCGGGCACAAC |
| IlvBN_BsaI_rev2 | TTGGTCTCCTCGAGTTACTGAAAAAACACCGCGATC |
| BsAlsS_BsaI_fwd1 | TTGGTCTCGGATCCGATGACAAAAGCAACAAAAG |
| BsAlsS_BsaI_rev2 | TTCGTCTCCTCGAGCTAGAGAGCTTTCGTTTTCATG |
| BmGDH_BsaI_fwd1 | TTGGTCTCGGATCCGATGTATAAAGATCTGGAAGG |
| BmGDH_BsaI_rev2 | TTGGTCTCCTCGAGTTAGCCACGACCTGCCTGAAAG |

**Table S3. List of plasmids and strains used in this study.**

| Name | Description | Reference |
| --- | --- | --- |
| pETDuet-1 | For protein expression under T7 promoter, Ap^R^ | Invitrogen |
| pCDFDuet-1 | For protein expression under T7 promoter, Sm^R^ | Invitrogen |
| pRSFDuet-1 | For protein expression under T7 promoter, Km^R^ | Invitrogen |
| pET28a | For protein expression under T7 promoter; Km^R^ | Invitrogen |
| pRSF-*Ec*ilvBN | pRSFDuet-1 derived with gene *Ec*ilvBN | This study |
| pRSF-*Bs*alsS | pRSFDuet-1 derived with gene *Bs*alsS | This study |
| pET-*Bs*alsS | pETDuet-1 derived with gene *Bs*alsS | This study |
| pET28a-IRG02 | pET28a derived with gene IRG02 | Ref. ^1^ |
| pCDF-*Bm*GDH | pCDFDuet-1 derived with gene *Bm*GDH | This study |
| pET28a-AspRedAm^Q240A^ | pET28a derived with gene *Asp*RedAm ^Q240A^ | Ref. ^2^ |
| pET28a-IR77^A208N^ | pET28a derived with gene IR77^A208N^ | Ref. ^3^ |

**Table S4. List of strains used in this study.**

| Name | Description |
| --- | --- |
| MG1655-RARE (MR) | For protein expression |
| MR-*Bs*AlsS | MR derived with plasmid pRSF-*Bs*alsS |
| MR-*Ec*IlvBN | MR derived with plasmid pRSF-*Ec*ilvBN |
| MR-IR77^A208N^-*Bm*GDH | MR derived with plasmid pET28a-IR77^A208N^ and pCDF-*Bm*GDH |
| MR-*Asp*RedAm^Q240A^-*Bm*GDH | MR derived with plasmid pET28a-*Asp*RedAm ^Q240A^ and pCDF-*Bm*GDH |
| MR-IRG02-*Bm*GDH | MR derived with plasmid pET28a-IRG02 and pCDF-*Bm*GDH |
| MR-*Bs*AlsS-IRG02-*Bm*GDH | MR derived with plasmid pET-*Bs*alsS, pET28a-IRG02 and pCDF-*Bm*GDH |

**Section 2 List of Supplementary Figures**


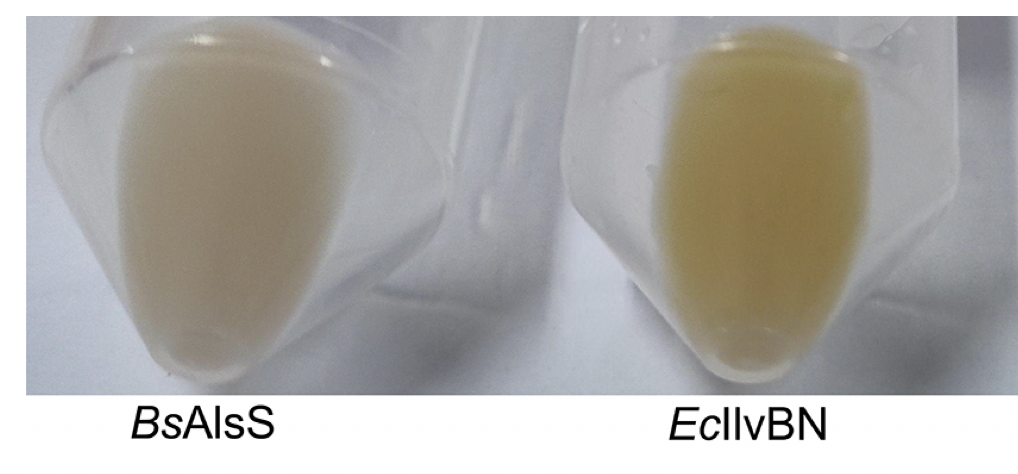


**Figure S1.** *E. coli* cells expressing the *Bs*AlsS and *Ec*IlvBN. MR-*Bs*AlsS is off-white. MR-*Ec*IlvBN appears yellow.


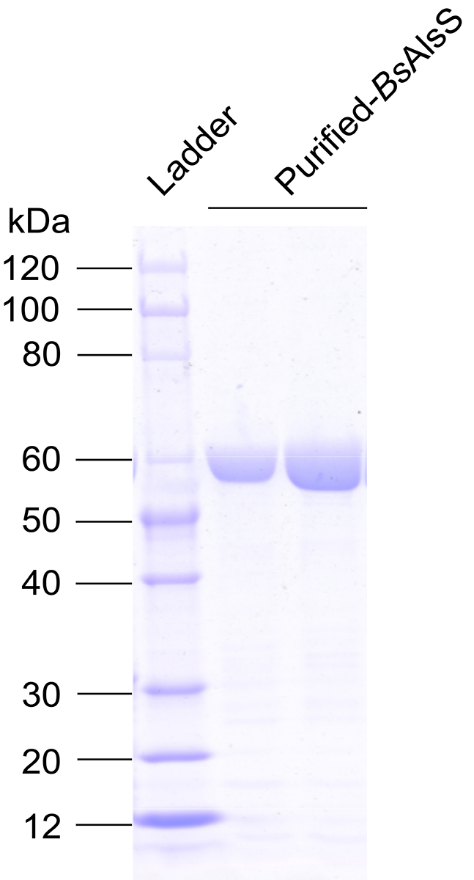


**Figure S2**. Purified *Bs*AlsS enzymes analyzed by 10% SDS-PAGE.

**
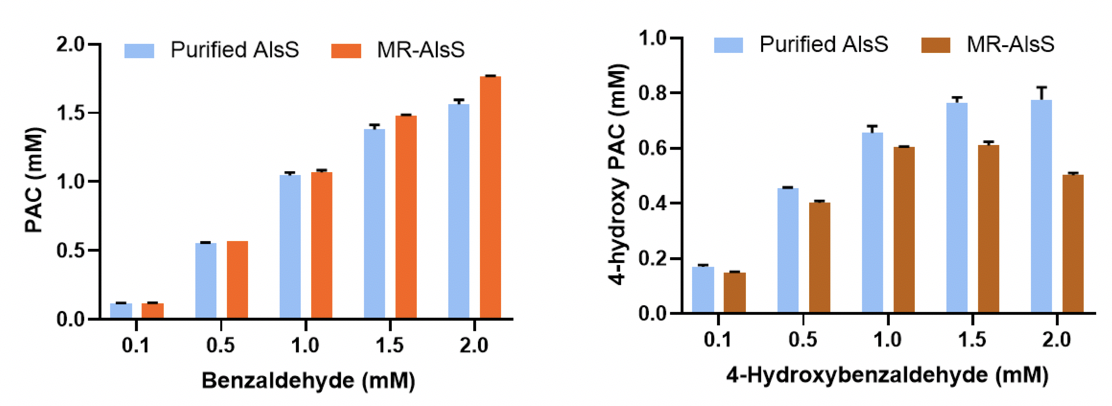
**

**Figure S3**. Comparison of biocatalytic efficiency of purified-*Bs*AlsS and the whole-cell biocatalysis by MR-*Bs*AlsS. The catalytic efficiency of 0.5 mg/ml CDW whole-cell biocatalyst (MR-*Bs*AlsS) and 0.5 mg/ml purified *Bs*AlsS was compared.


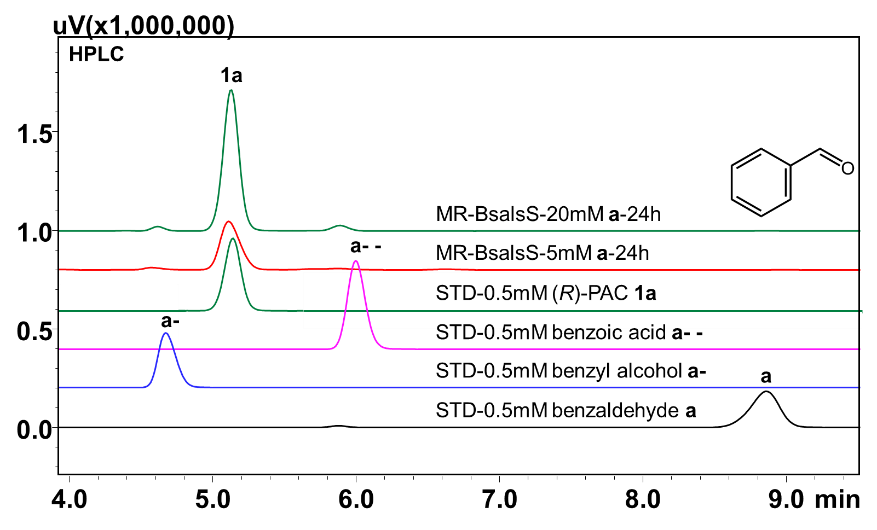


**Figure S4.1** HPLC analysis of *Bs*AlsS-catalyzed benzaldehyde **a** and pyruvate **1** reaction, the standards of target product 1-hydroxy-1-phenylpropan-2-one **1a** and byproduct **a-**, **a- -**.


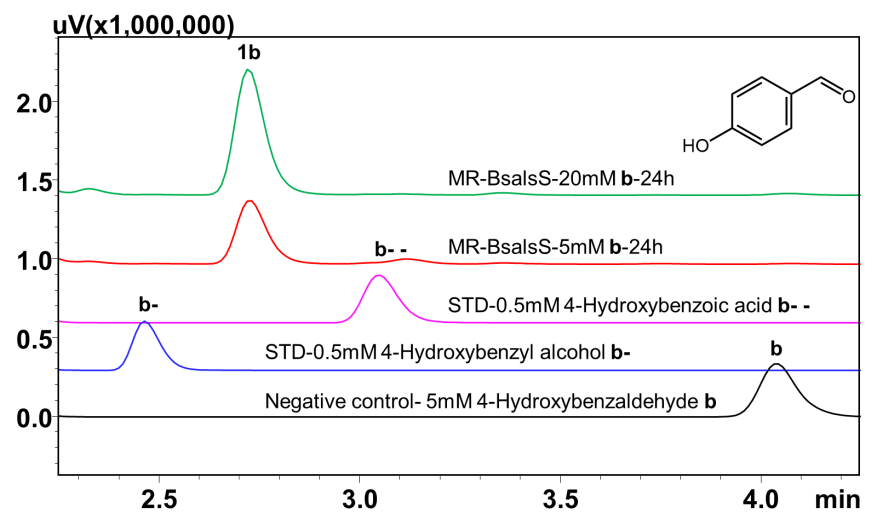


**Figure S4.2** HPLC analysis of *Bs*AlsS-catalyzed 4-hydroxybenzaldehyde **b** and pyruvate **1** reaction, the target product 1-hydroxy-1-(4-hydroxyphenyl)propan-2-one **1b** and byproduct **b-, b- -**.


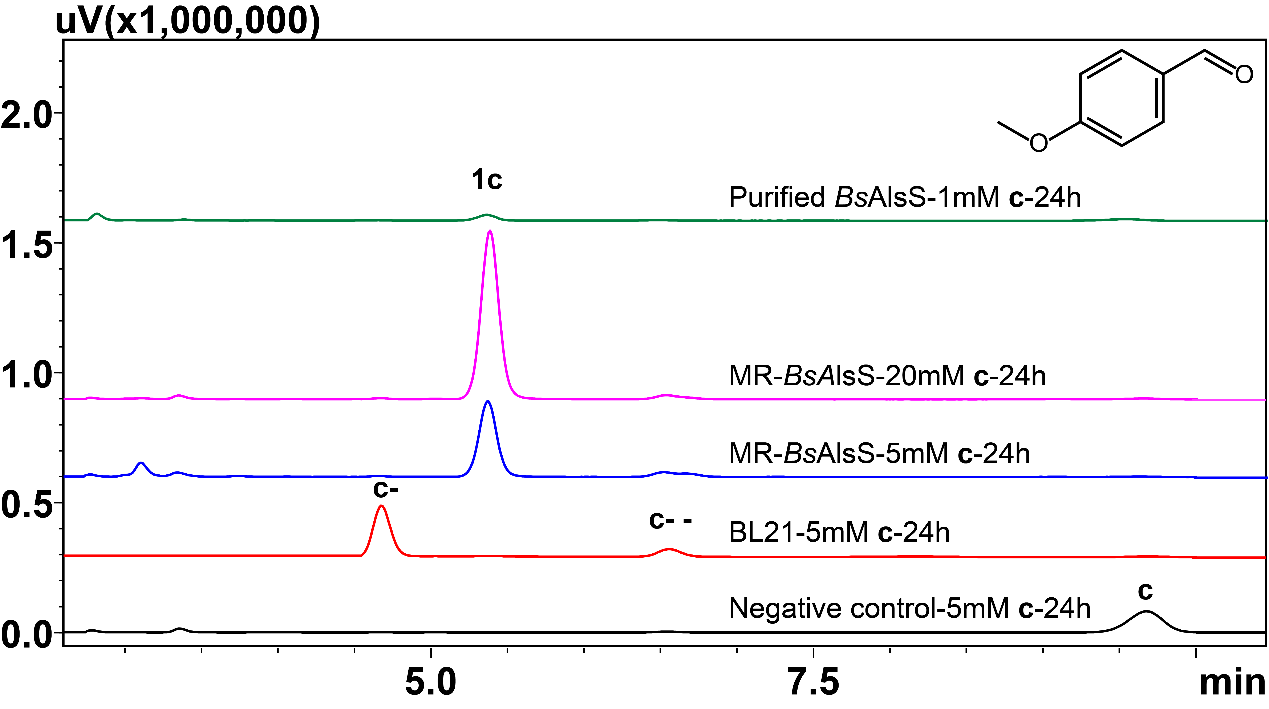


**Figure S4.3** HPLC analysis of *Bs*AlsS-catalyzed 4-methoxybenzaldehyde **c** and pyruvate **1** reaction, the target product 1-hydroxy-1-(4-methoxyphenyl)propan-2-one **1c**.

**
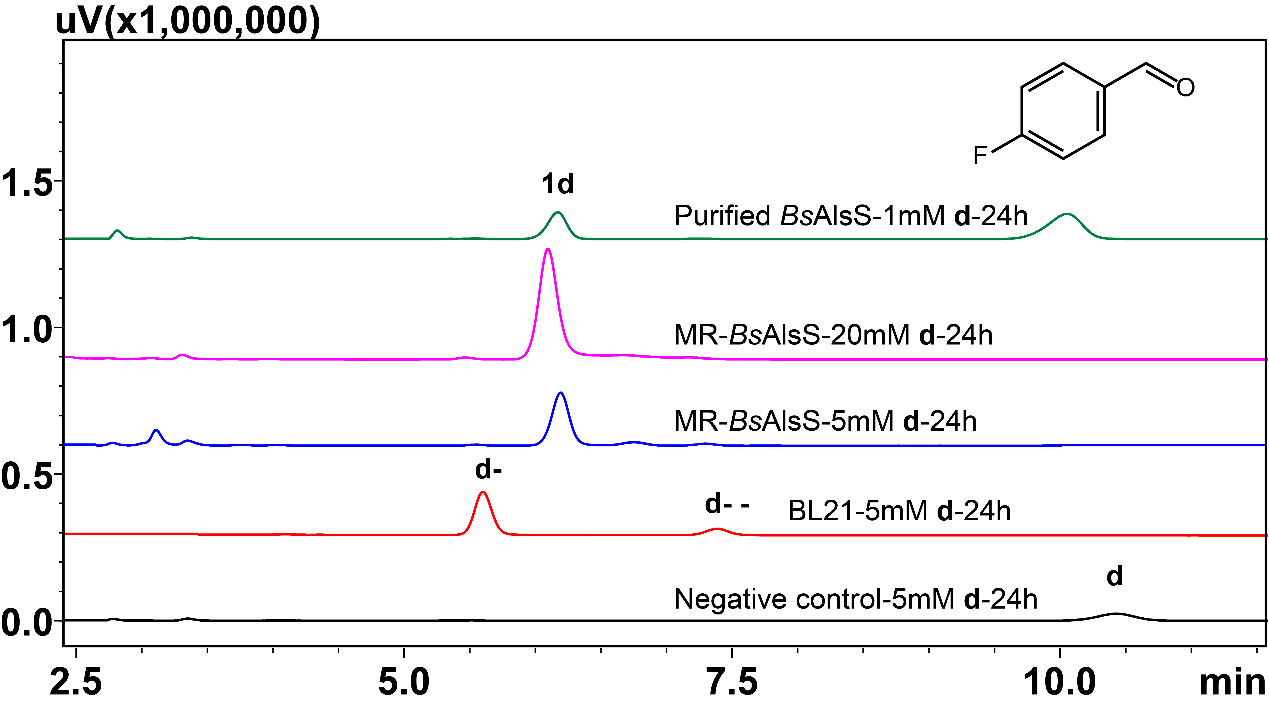
**

**Figure S4.4** HPLC analysis of *Bs*AlsS-catalyzed 4-fluorobenzaldehyde **d** and pyruvate **1** reaction, the target product 1-(4-fluorophenyl)-1-hydroxypropan-2-one **1d**.


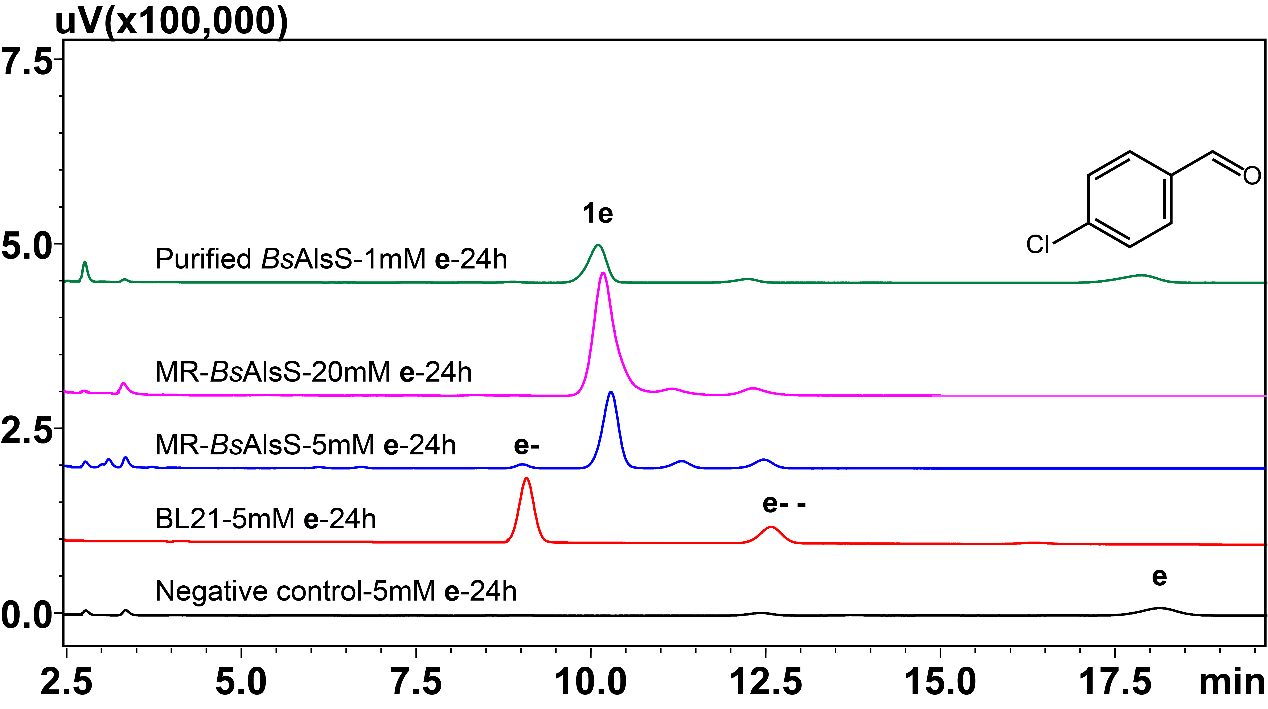


**Figure S4.5** HPLC analysis of *Bs*AlsS-catalyzed 4-chlorobenzaldehyde **e** and pyruvate **1** reaction, the target product 1-(4-chlorophenyl)-1-hydroxypropan-2-one **1e**.


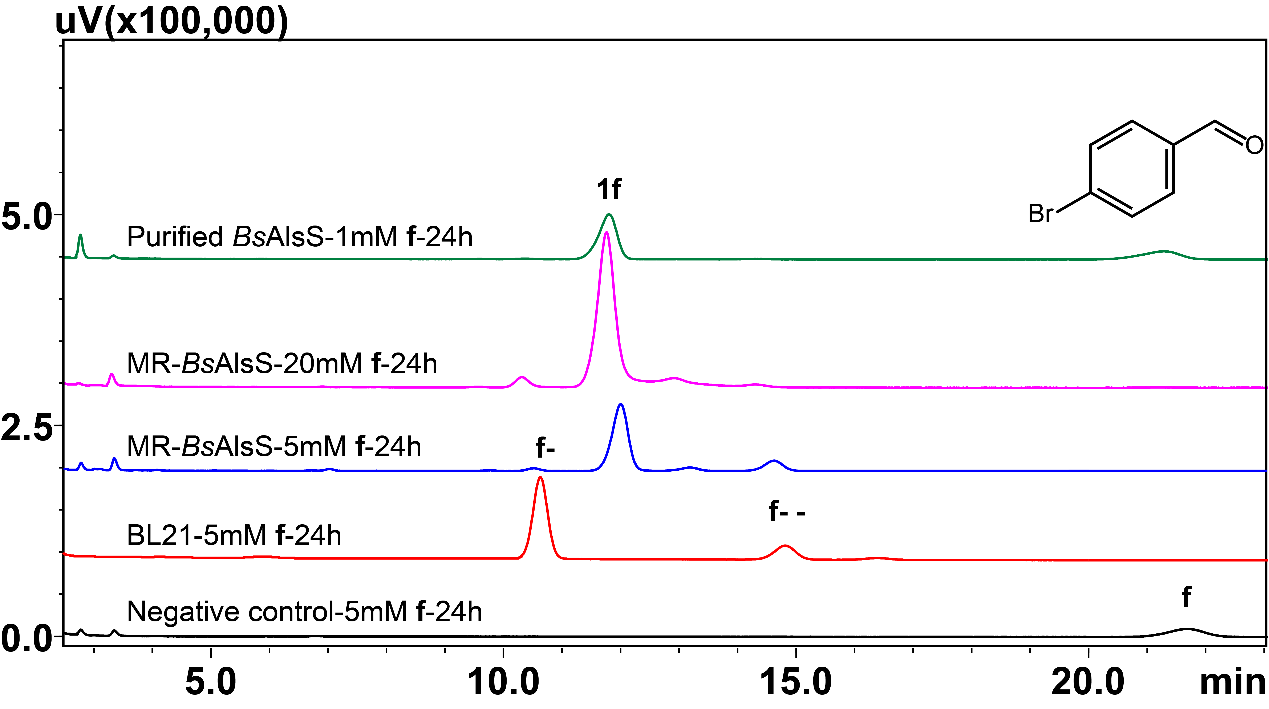


**Figure S4.6** HPLC analysis of *Bs*AlsS-catalyzed 4-bromobenzaldehyde **f** and pyruvate **1** reaction, the target product 1-(4-bromophenyl)-1-hydroxypropan-2-one **1f**.


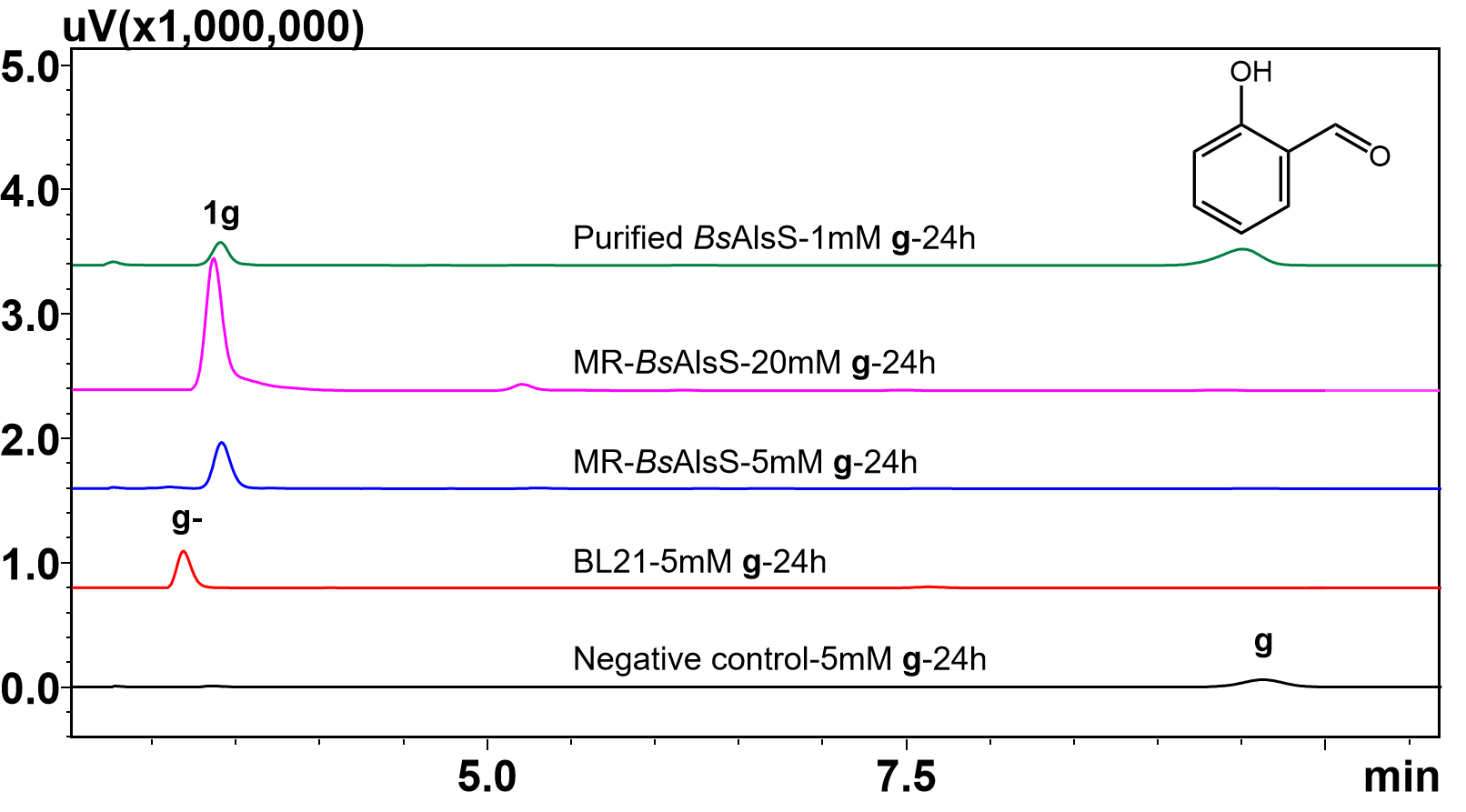


**Figure S4.7** HPLC analysis of *Bs*AlsS-catalyzed 2-hydroxybenzaldehyde **g** and pyruvate **1** reaction, the target product 1-hydroxy-1-(2-hydroxyphenyl)propan-2-one **1g**.


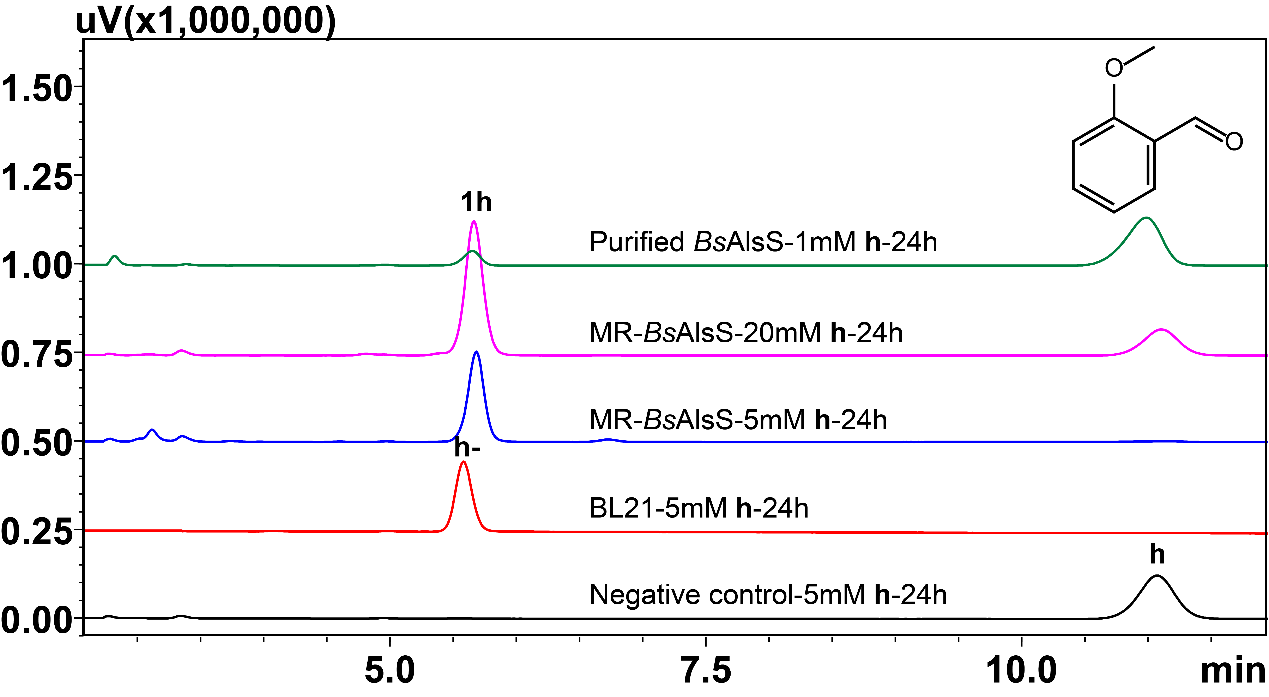


**Figure S4.8** HPLC analysis of *Bs*AlsS-catalyzed 2-methoxybenzaldehyde **h** and pyruvate **1** reaction, the target product 1-hydroxy-1-(2-methoxyphenyl)propan-2-one **1h**.


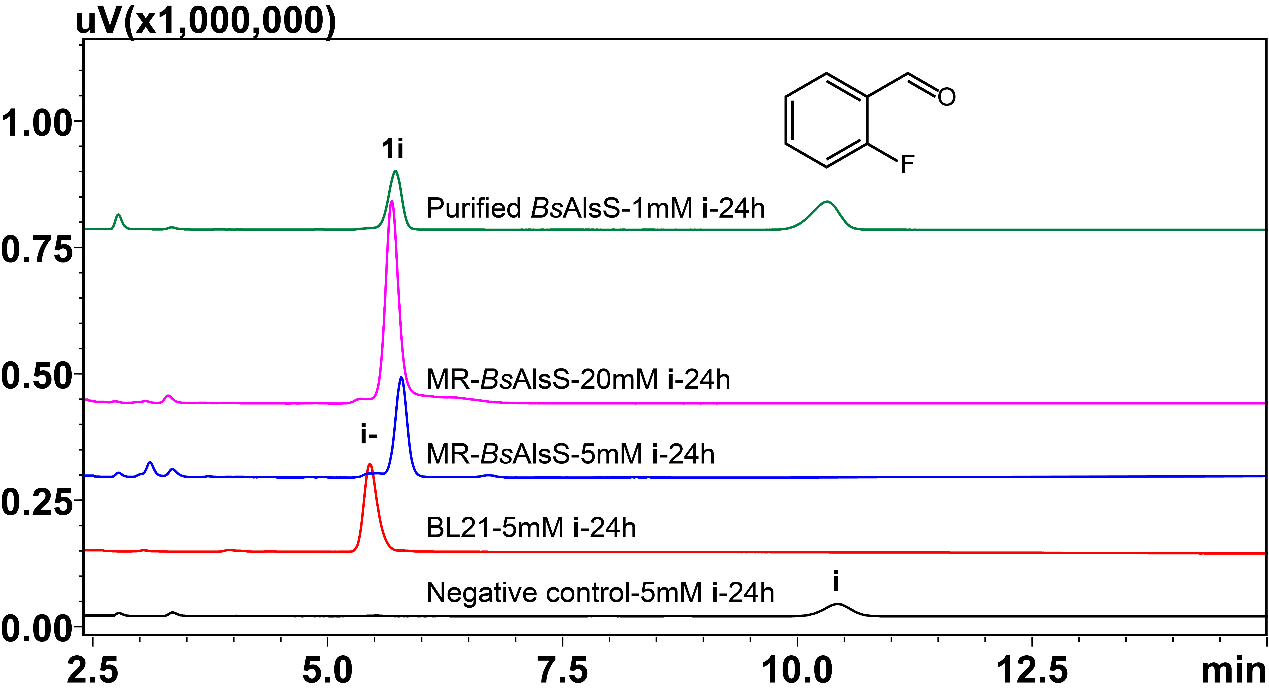


**Figure S4.9** HPLC analysis of *Bs*AlsS-catalyzed 2-methoxybenzaldehyde **i** and pyruvate **1** reaction, the target product 1-hydroxy-1-(2-methoxyphenyl)propan-2-one **1i**.


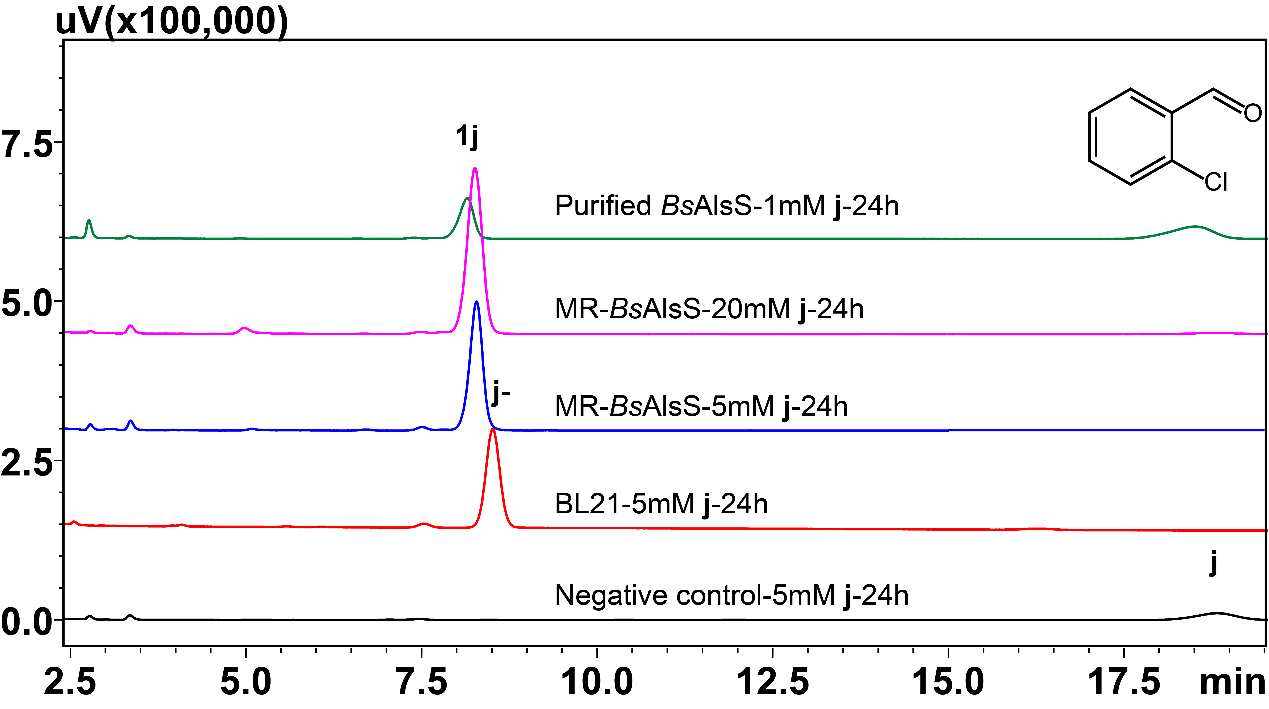


**Figure S4.10** HPLC analysis of *Bs*AlsS-catalyzed 2-Methoxybenzaldehyde **j** and pyruvate **1** reaction, the target product 1-hydroxy-1-(2-methoxyphenyl)propan-2-one **1j**.


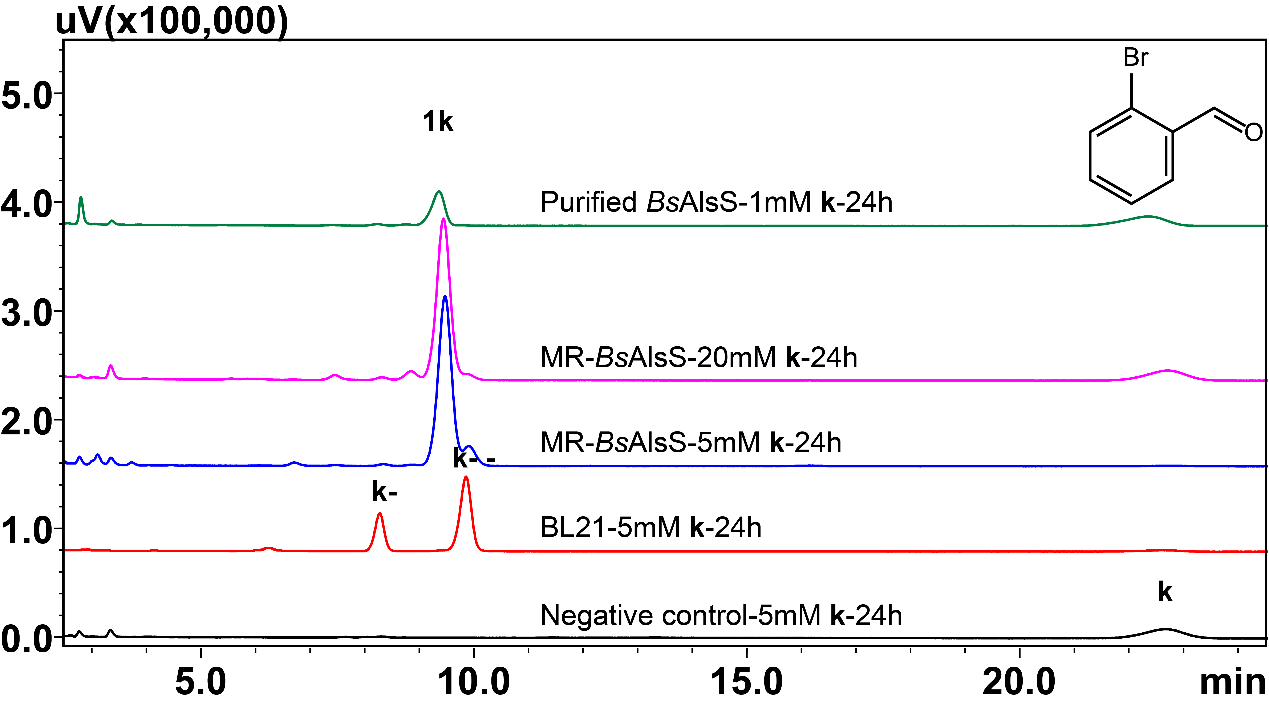


**Figure S4.11** HPLC analysis of *Bs*AlsS-catalyzed 2-Bromobenzaldehyde **k** and pyruvate **1** reaction, the target product 1-(2-bromophenyl)-1-hydroxypropan-2-one **1k**.


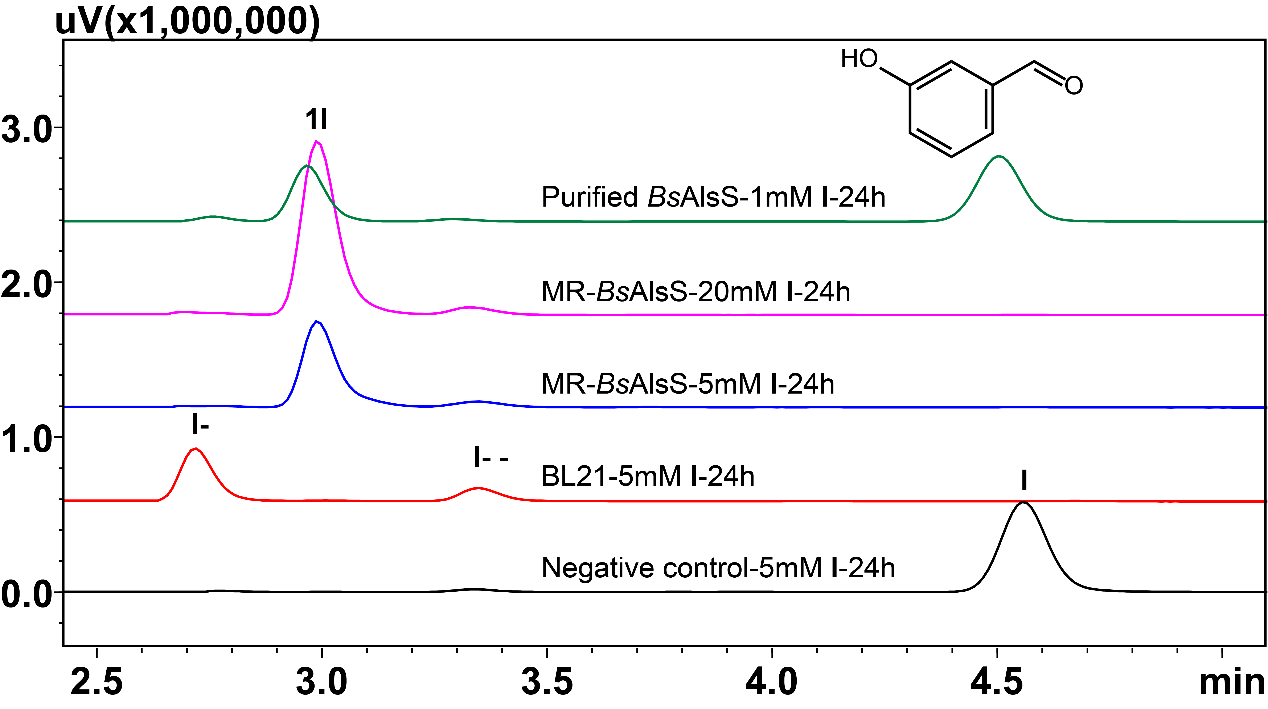


**Figure S4.12** HPLC analysis of *Bs*AlsS-catalyzed 3-hydroxybenzaldehyde **l** and pyruvate **1** reaction, the target product 1-hydroxy-1-(3-hydroxyphenyl)propan-2-one **1l**.


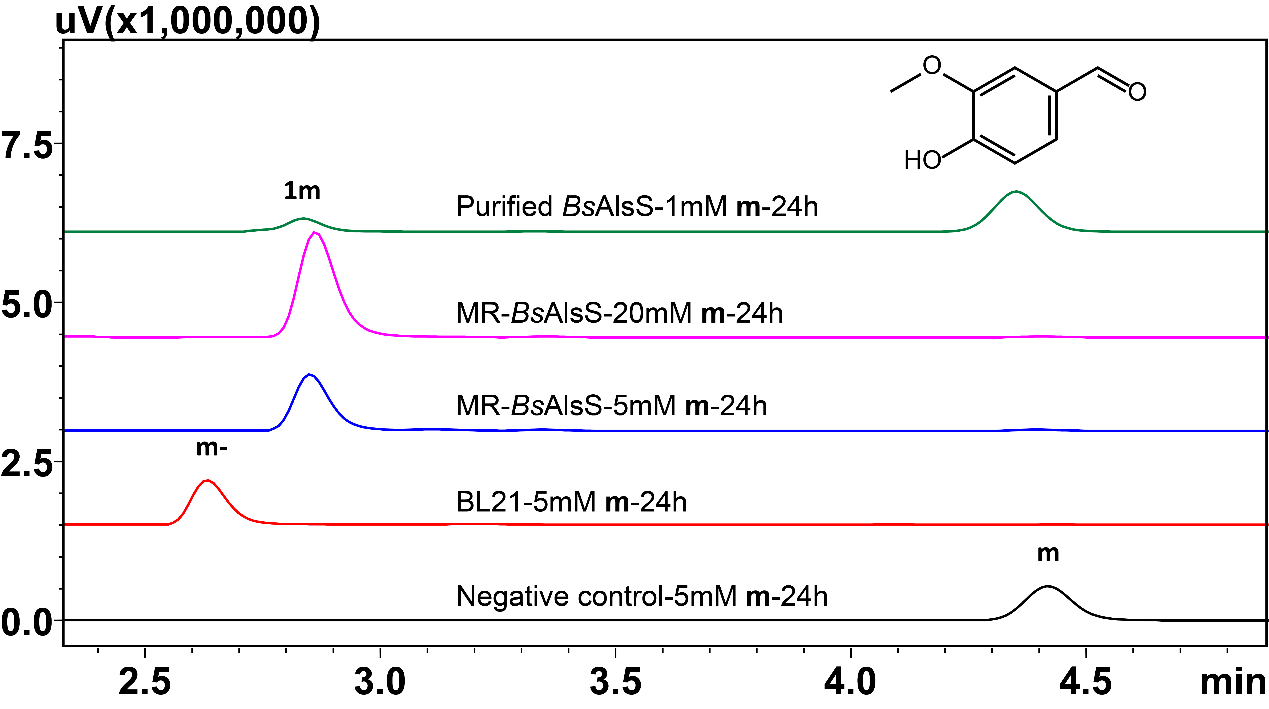


**Figure S4.13** HPLC analysis of *Bs*AlsS-catalyzed 3-methoxy-4-hydroxybenzaldehyde **m** and pyruvate **1** reaction, the target product 1-hydroxy-1-(4-hydroxy-3-methoxyphenyl)propan-2-one **1m**.

**
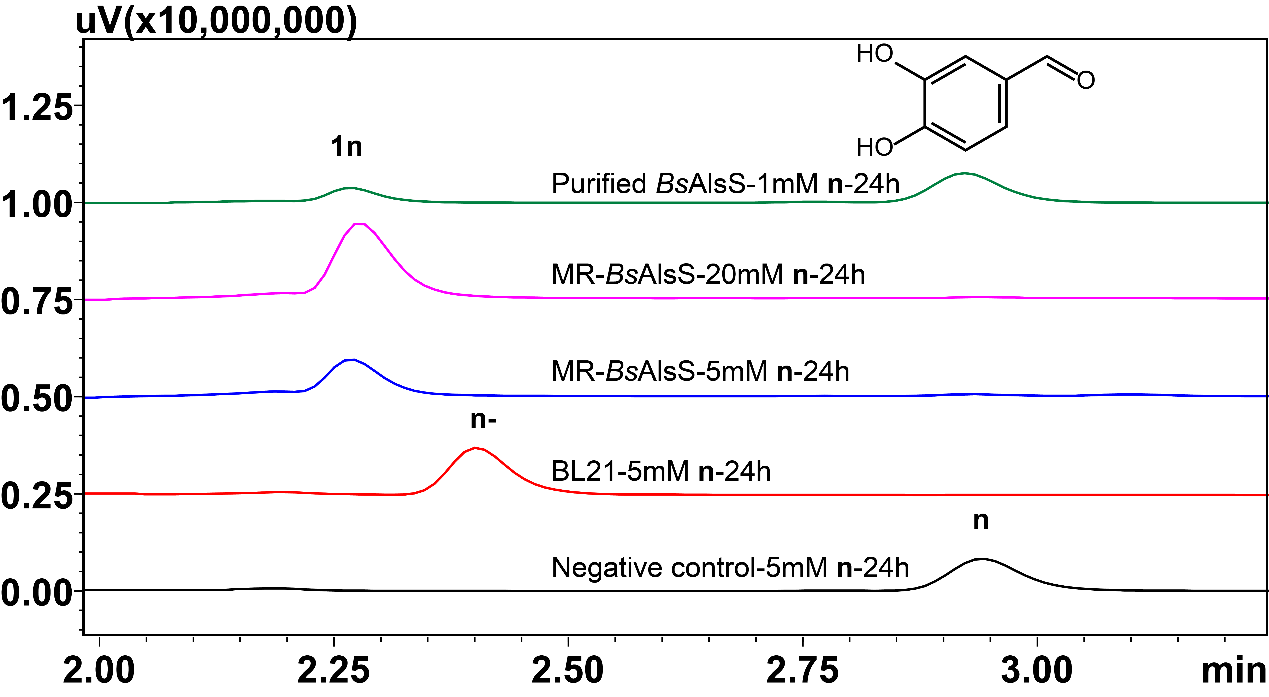
**

**Figure S4.14** HPLC analysis of *Bs*AlsS-catalyzed 3,4-Dihydroxybenzaldehyde **n** and pyruvate **1** reaction, the target product 1-(3,4-dihydroxyphenyl)-1-hydroxypropan-2-one **1n**.


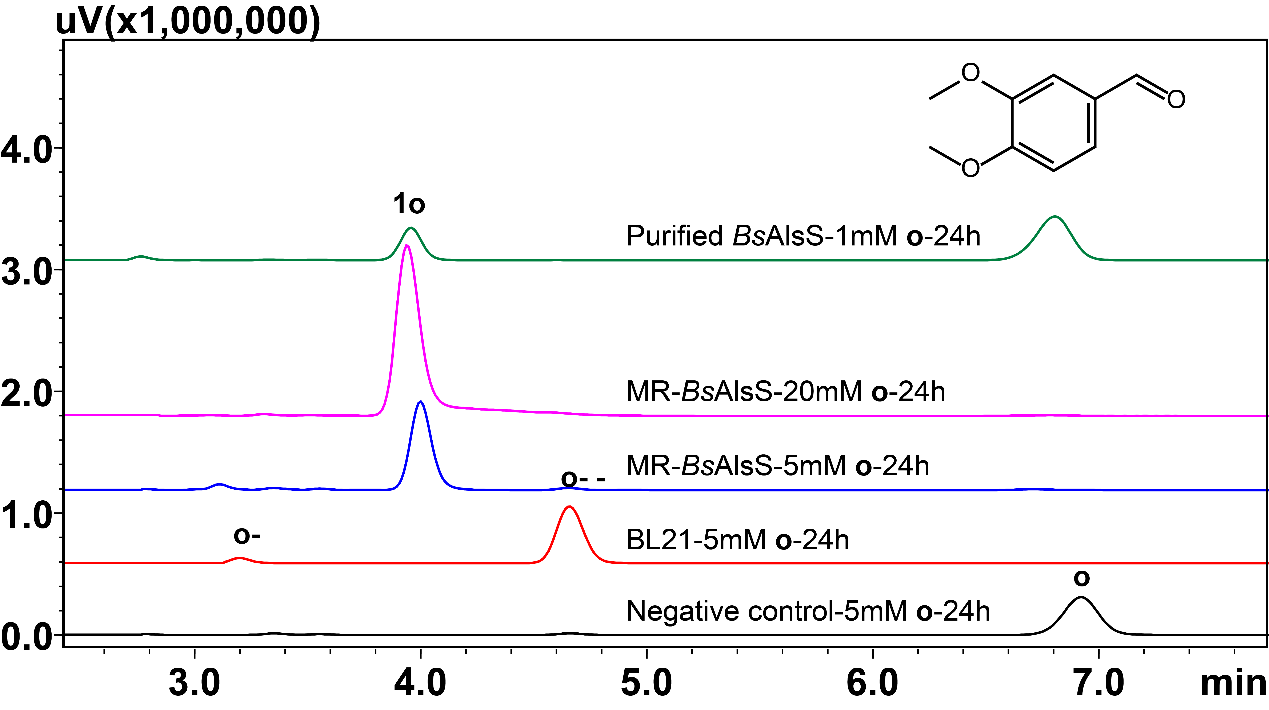


**Figure S4.15** HPLC analysis of *Bs*AlsS-catalyzed 3,4-dimethoxybenzaldehyde **o** and pyruvate **1** reaction, the target product 1-(3,4-dimethoxyphenyl)-1-hydroxypropan-2-one **1o**.


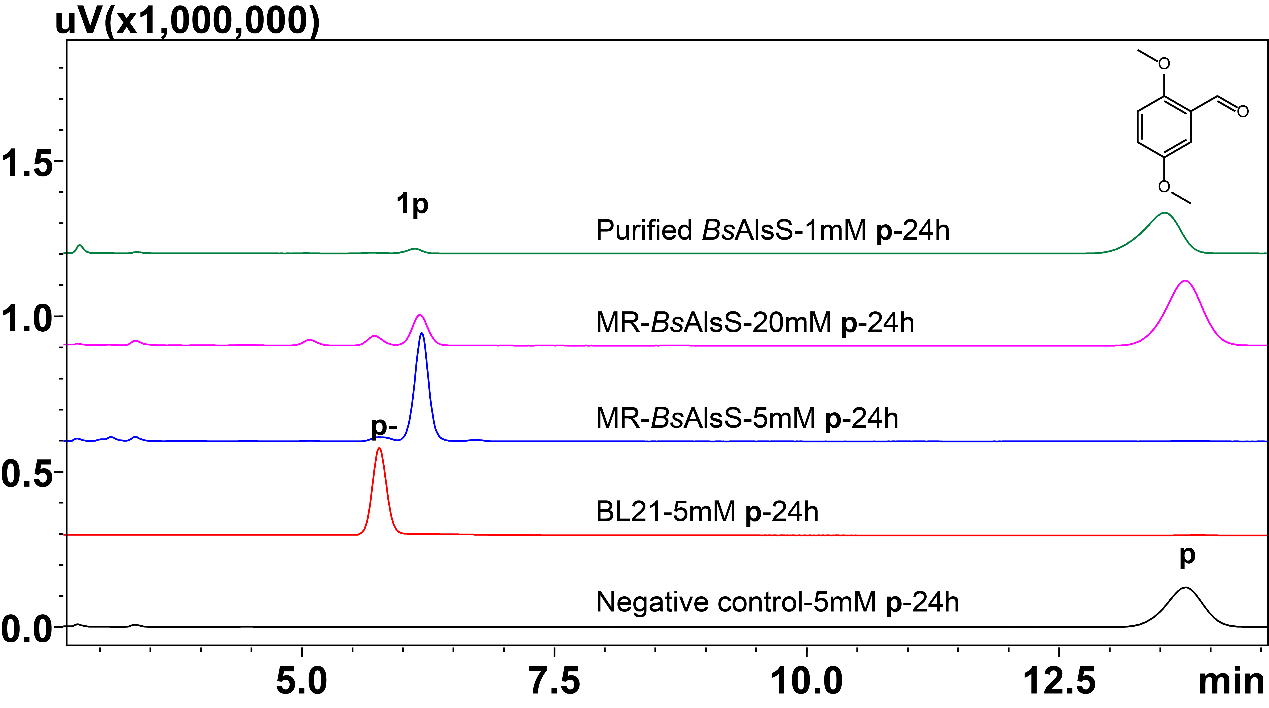


**Figure S4.16** HPLC analysis of *Bs*AlsS-catalyzed 3,4-dimethoxybenzaldehyde **p** and pyruvate **1** reaction, the target product 1-(3,4-dimethoxyphenyl)-1-hydroxypropan-2-one **1p**.

**
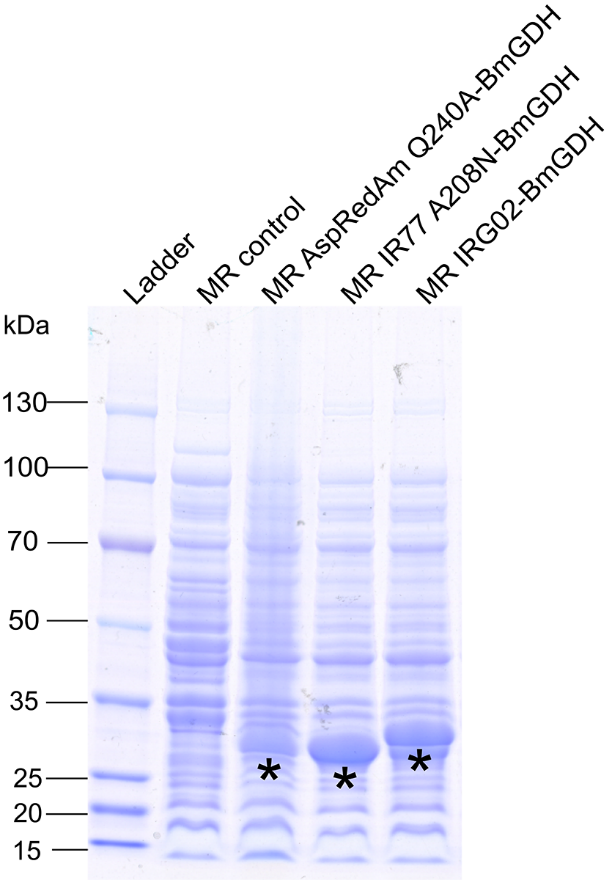
**

**Figure S5** SDS-PAGE analysis of the whole-cell catalysts coexpressing IREDs and *Bm*GDH. Lane 1 is the protein standard. Lane 2 is the negative control of empty MG1655 RARE, Lane 3 is the MR derivative with *Asp*RedAm ^Q240A^ and *Bm*GDH. Lane 4 is the MR derivative with IR77 ^A208N^ and *Bm*GDH, Lane 5 is the MR derivative with IRG02 and *Bm*GDH.

**
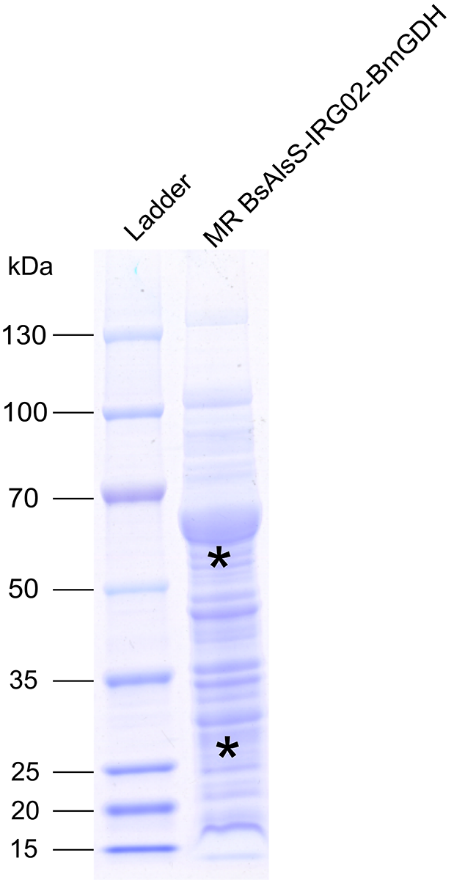
**

**Figure S6.1** SDS-PAGE analysis of the whole-cell biocatalyst with *Bs*AlsS, IRG02, and *Bm*GDH. Lane 1 is the protein standard. Lane 2 is the MR derivative with *Bs*AlsS, IRG02, and *Bm*GDH.


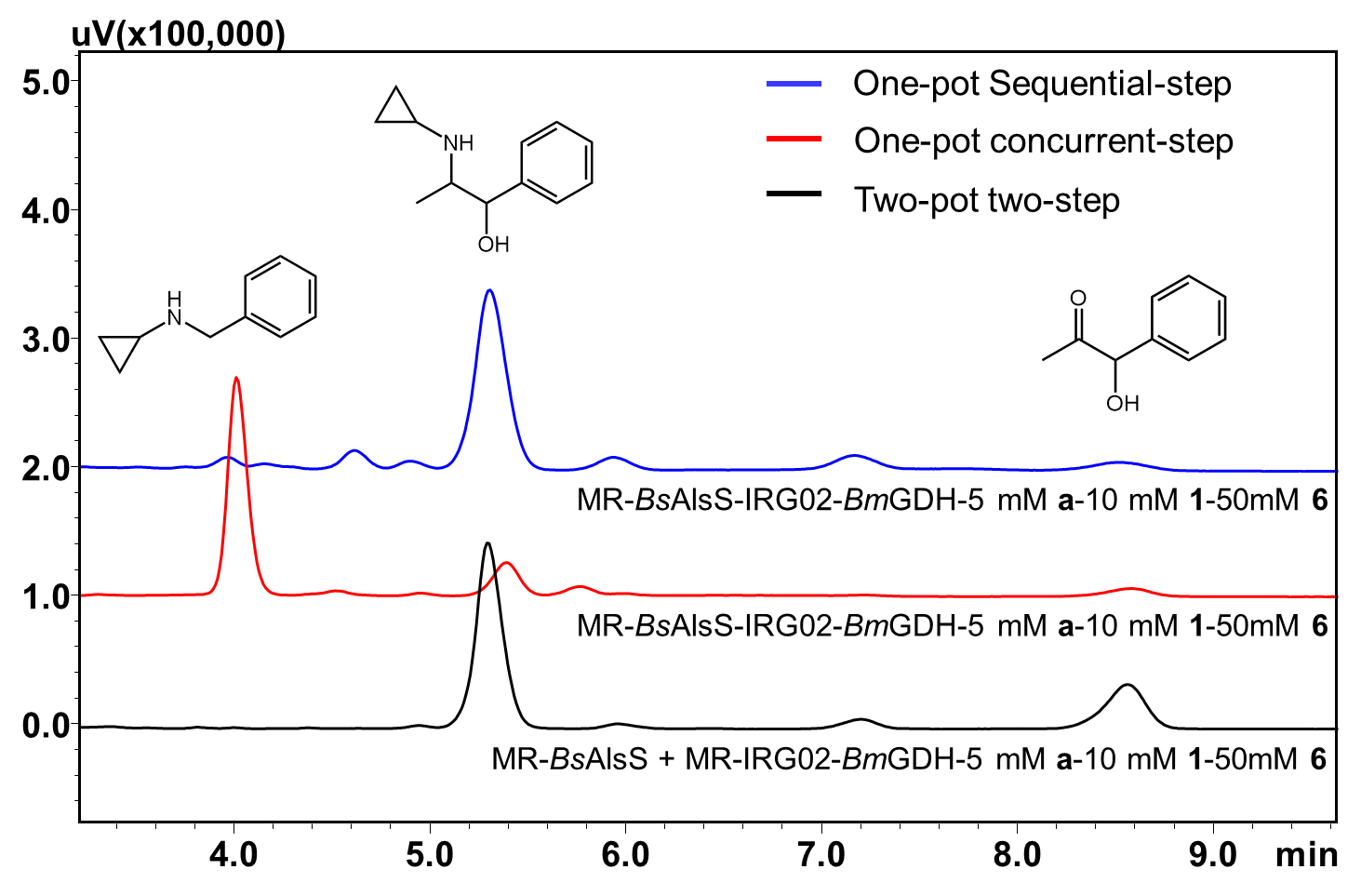


**Figure S6.2** HPLC analysis of three ways to catalyze **a**, **1** and **6**. Black line represents the catalytic result of two-pot two-step manner. Red line represents the catalytic result of one-pot concurrent-step. Blue line represents the catalytic result of one-pot sequential-step.

**
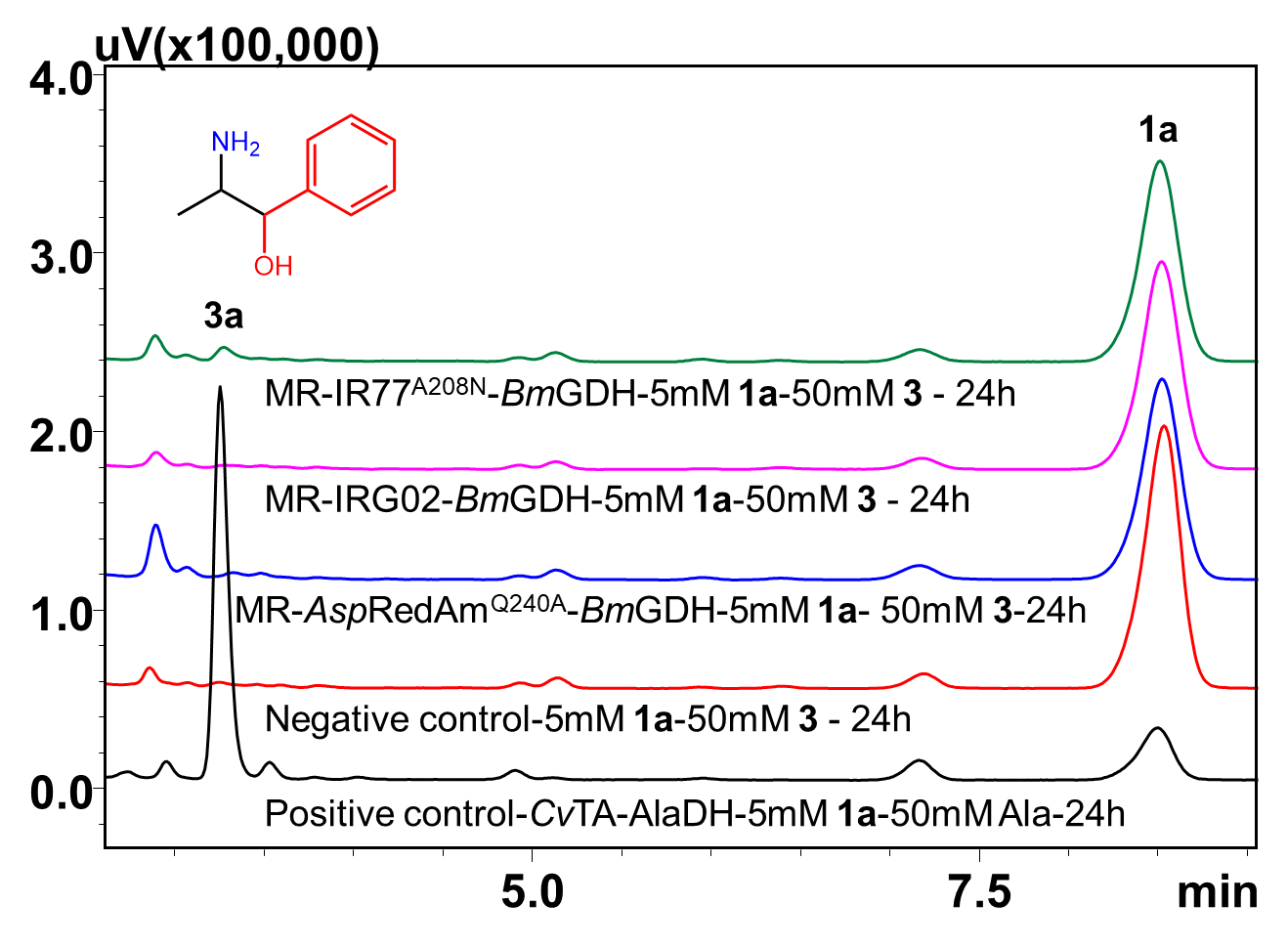
**

**Figure S7.1** IRED-catalysed reductive amination of **1a** with **3** (1:10 ratio), showing MR-*Cv*TA-AlaDH as the positive control, no *E. coli* cell as the negative control, MR-*Asp*RedAm^Q240A^-*Bm*GDH, MR-IRG02-*Bm*GDH, MR-IR77 ^A208N^-*Bm*GDH catalyzed samples.

**
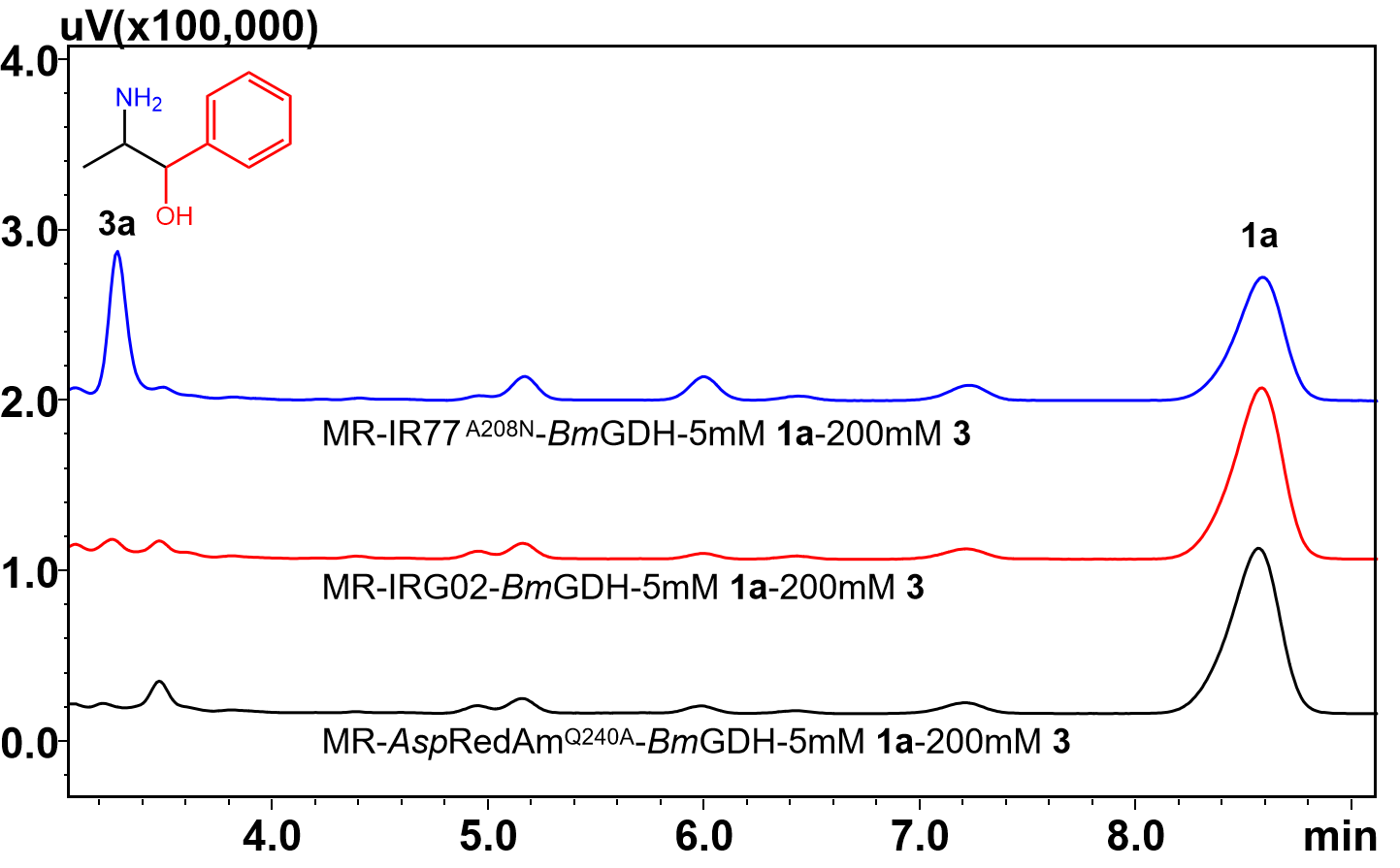
**

**Figure S7.2** The effect of the ratio of **1a** and **3** for MR-IR77^A208N^-*Bm*GDH**.**


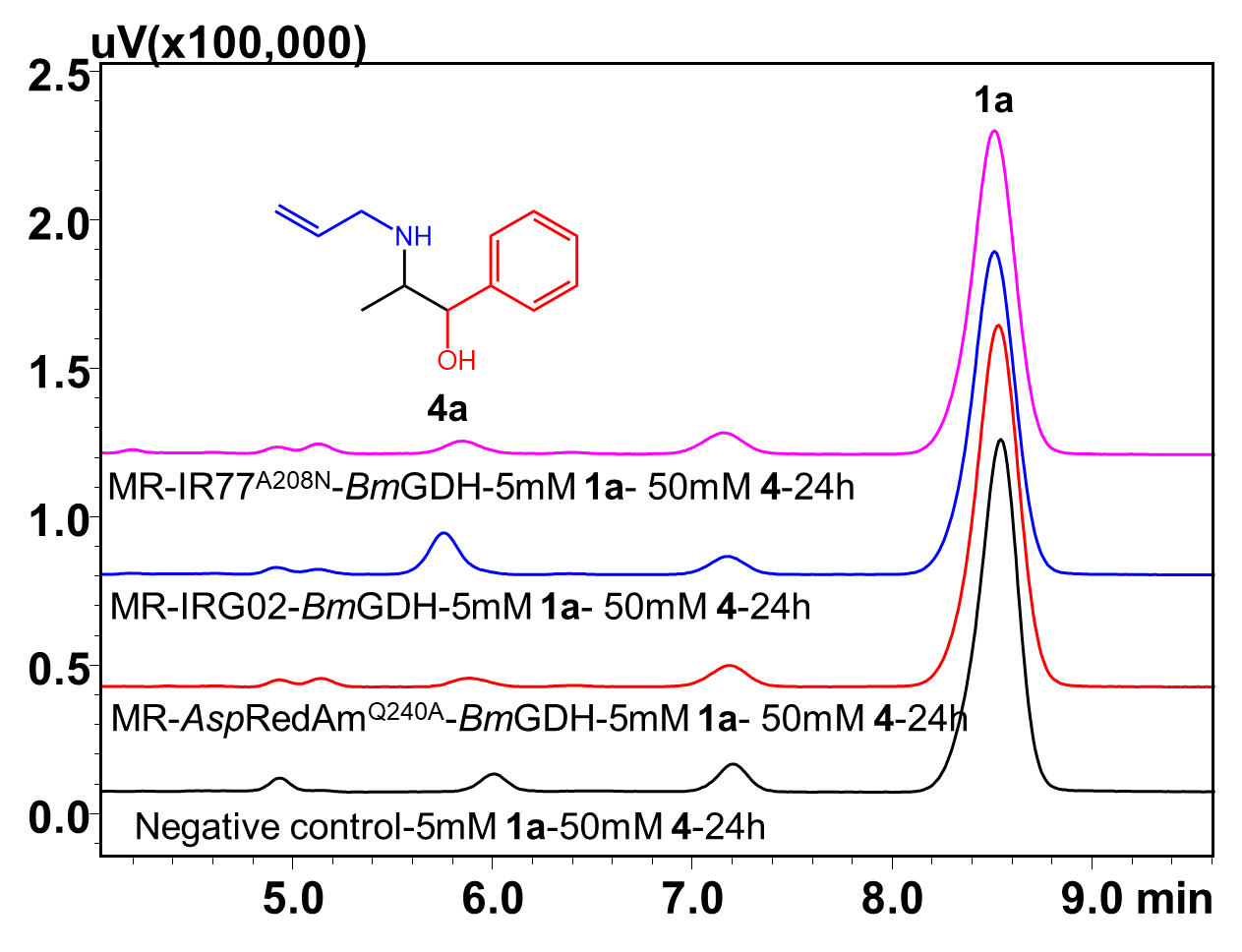


**Figure S8.1** IRED-catalysed reductive amination of **1a** with **4** (1:10 ratio), showing no *E. coli* cell as the negative control, MR-*Asp*RedAm^Q240A^-*Bm*GDH, MR-IRG02-*Bm*GDH, MR-IR77^A208N^-*Bm*GDH catalyzed samples.


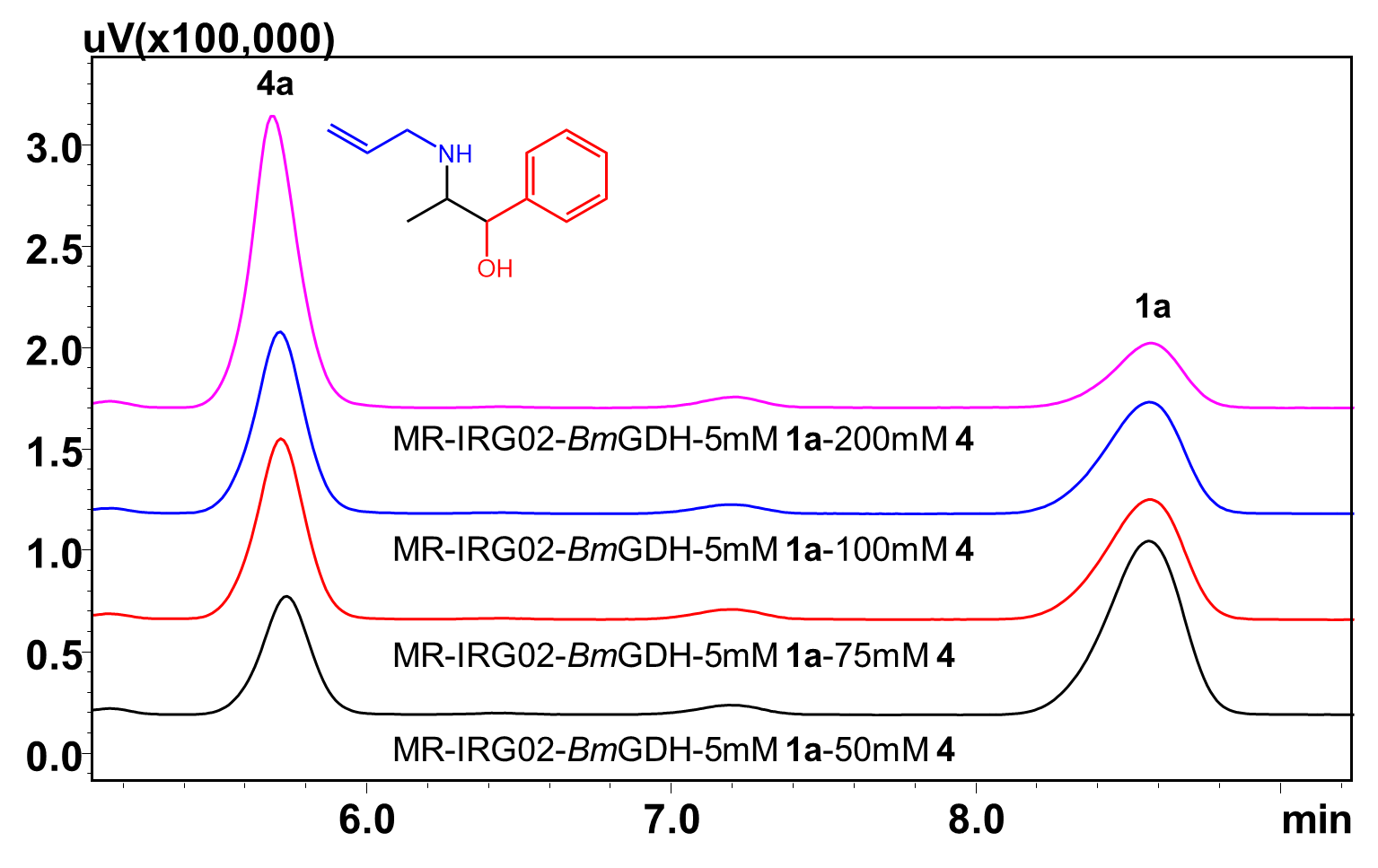


**Figure S8.2** The effect of the ratio of **1a** and **4** for MR-IRG02-*Bm*GDH mediated biocatalysis.


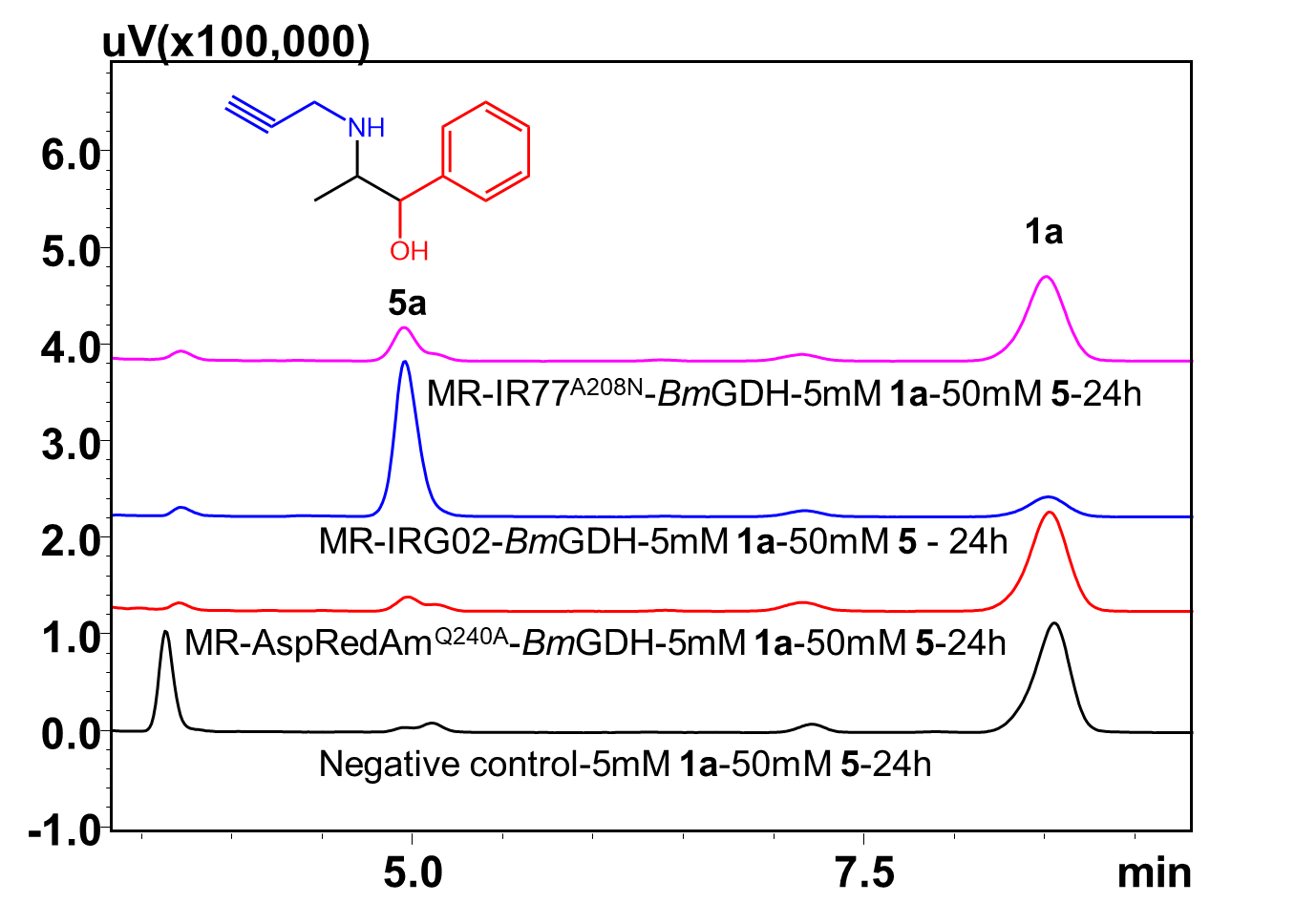


**Figure S9.1** IRED-catalysed reductive amination of **1a** with **5** (1:10 ratio), showing no *E. coli* cell as the negative control, MR-*Asp*RedAm^Q240A^-*Bm*GDH, MR-IRG02-*Bm*GDH, MR-IR77^A208N^-*Bm*GDH catalyzed samples.

**
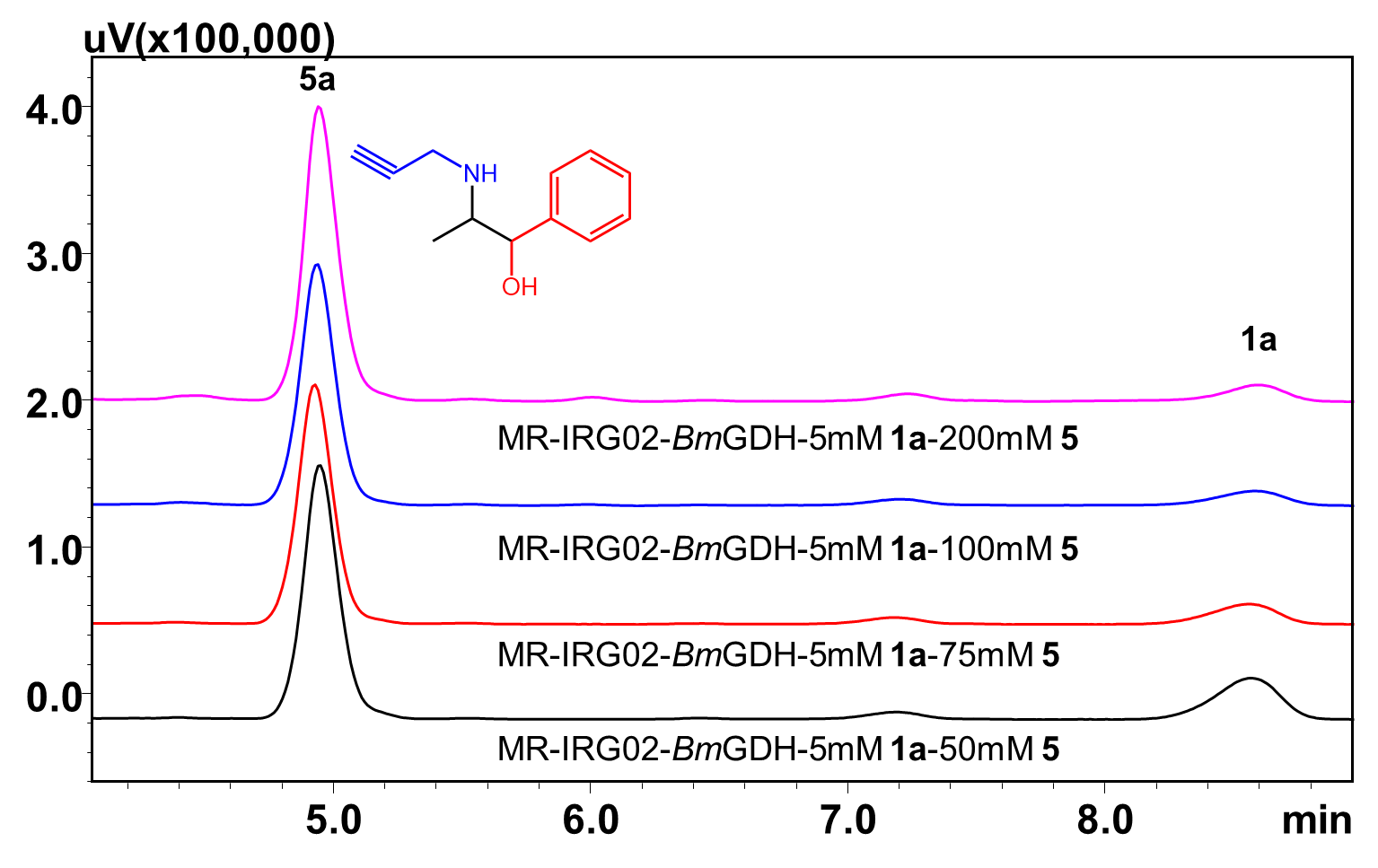
**

**Figure S9.2** The effect of the ratio of **1a** and **5** for MR-IRG02-*Bm*GDH mediated biocatalysis.


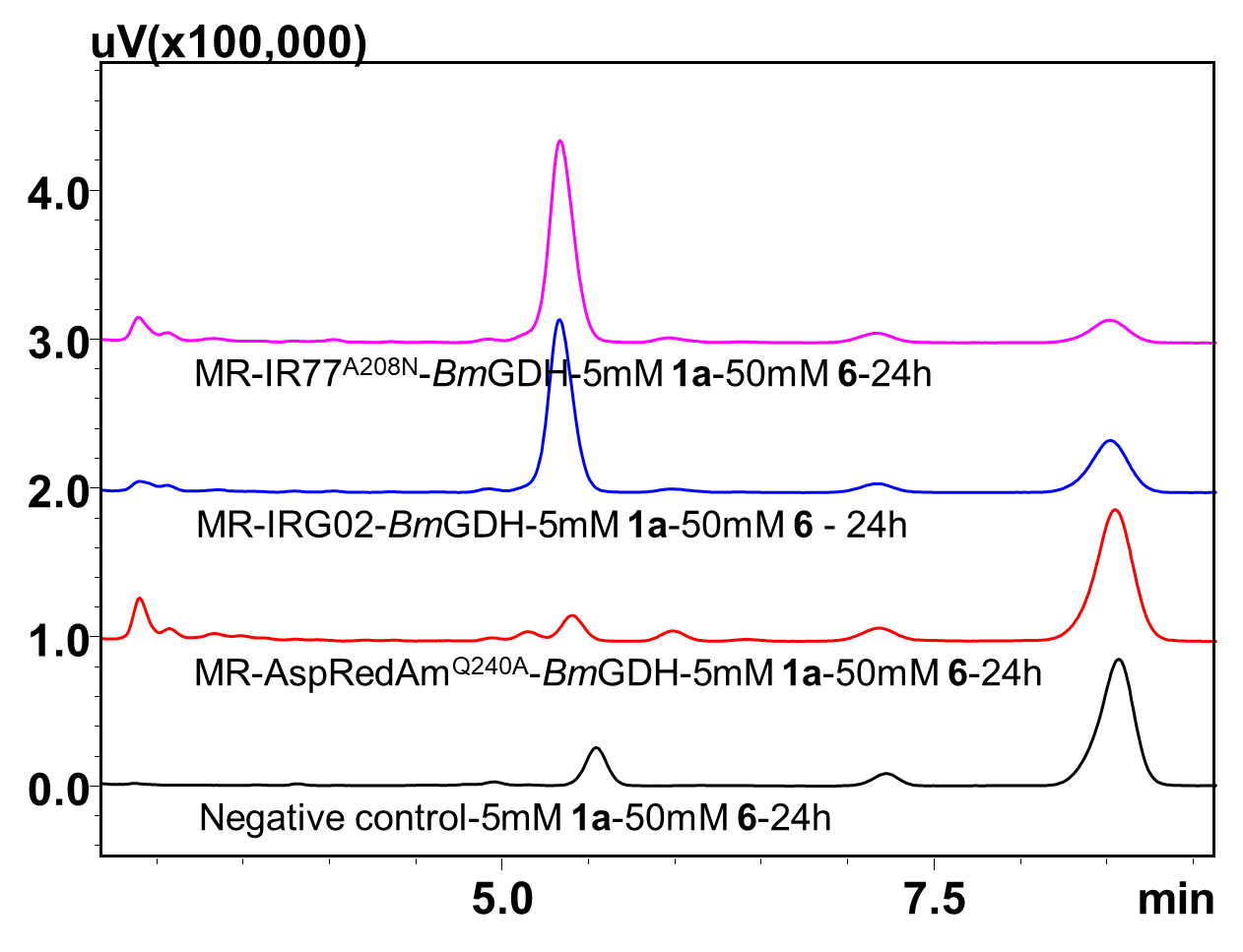


**Figure S10** IRED-catalysed reductive amination of **1a** with **6** (1:10 ratio), showing no *E. coli* cell as the negative control, MR-*Asp*RedAm^Q240A^-*Bm*GDH, MR-IRG02-*Bm*GDH, MR-IR77^A208N^-*Bm*GDH catalyzed samples.


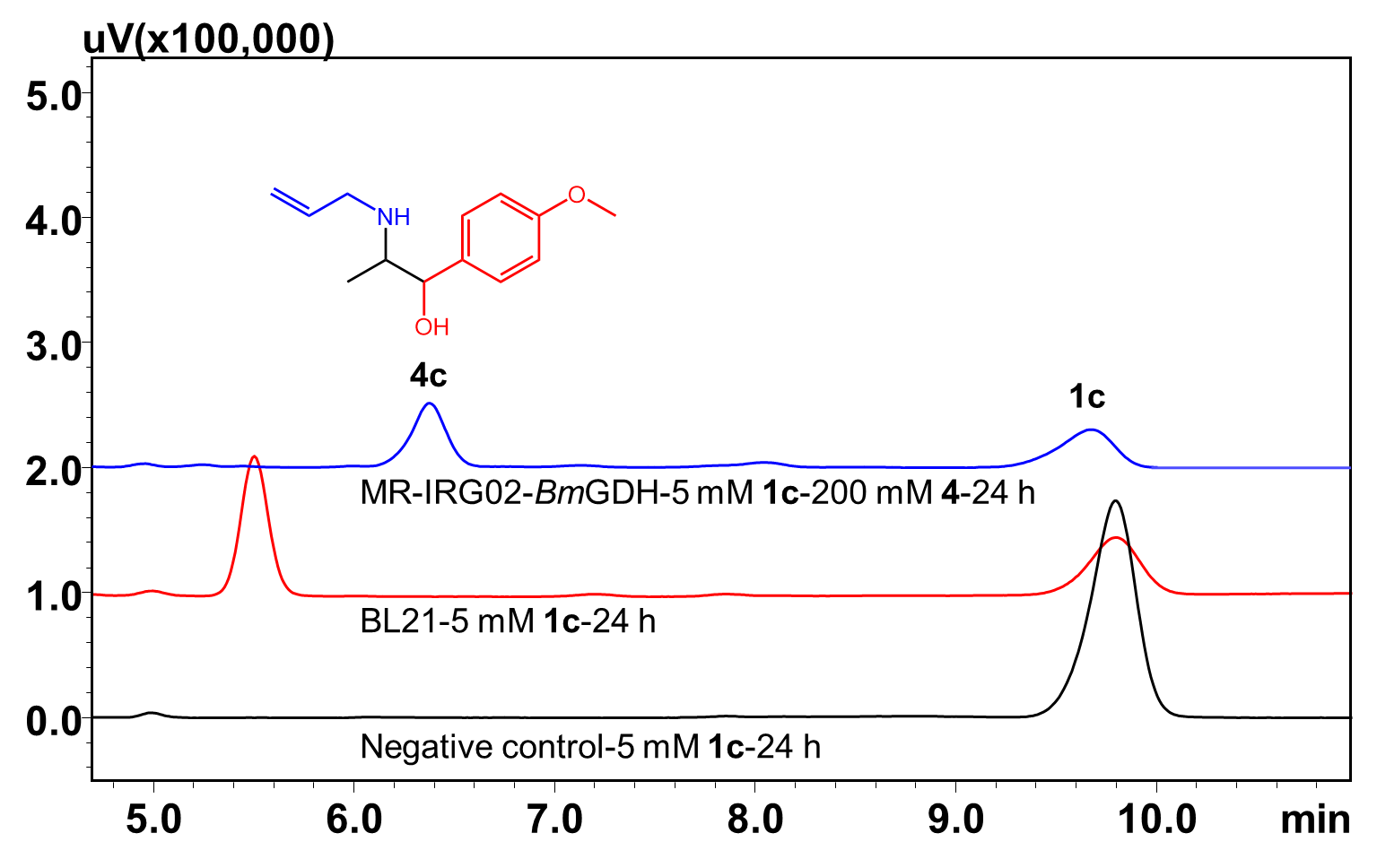


**Figure S11.1** HPLC analysis of MR-IRG02-*Bm*GDH catalyzed reductive amination of PAC analog **1c** with amine **4**. The negative control is the BL21 catalyzed reaction with possible by-product formation. The targeted product is **4c**.

**
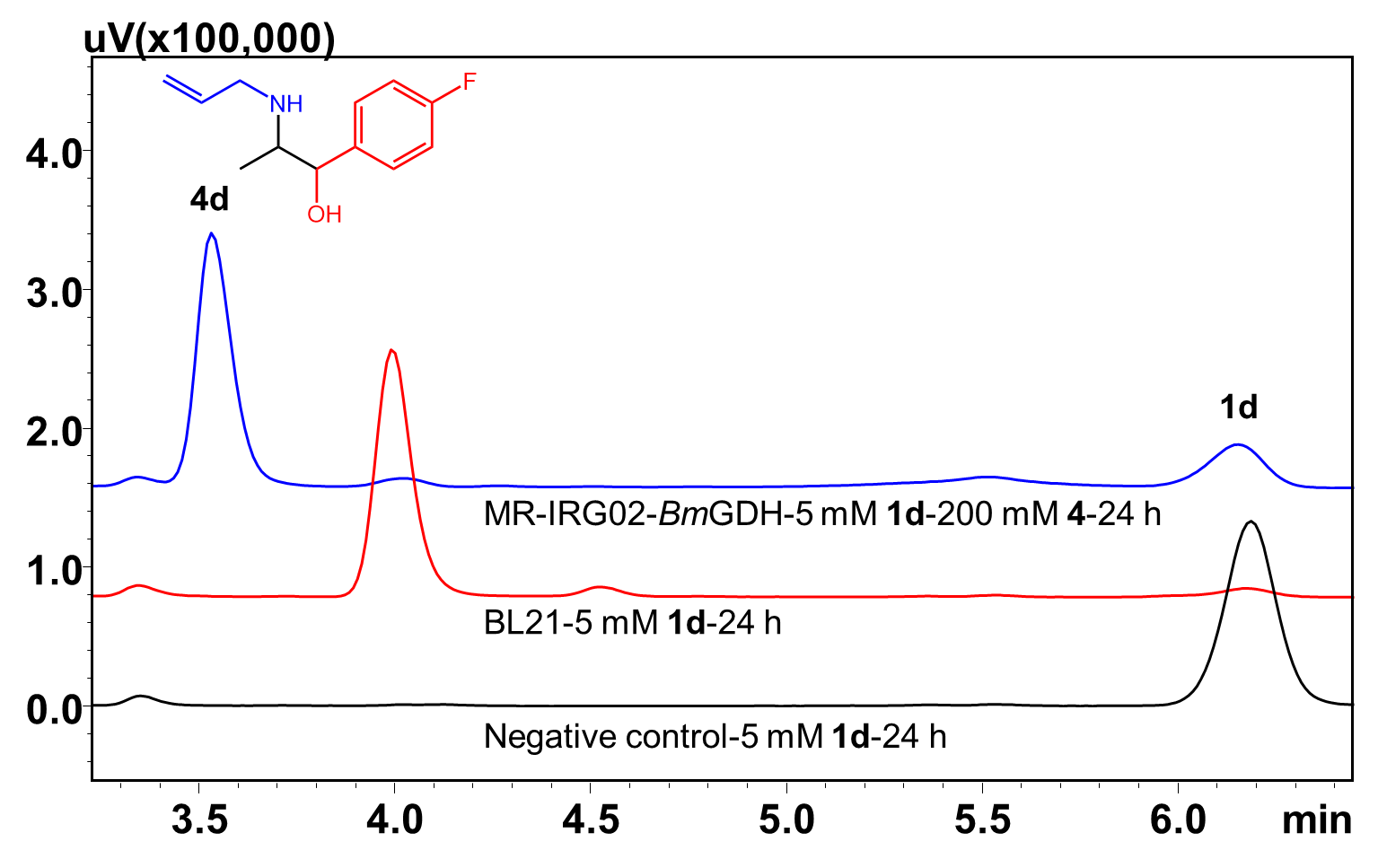
**

**Figure S11.2** HPLC analysis of MR-IRG02-*Bm*GDH catalyzed reductive amination of PAC analog **1d** with amine **4**. The negative control is the BL21 catalyzed reaction with possible by-product formation. The targeted product is **4d**.

**
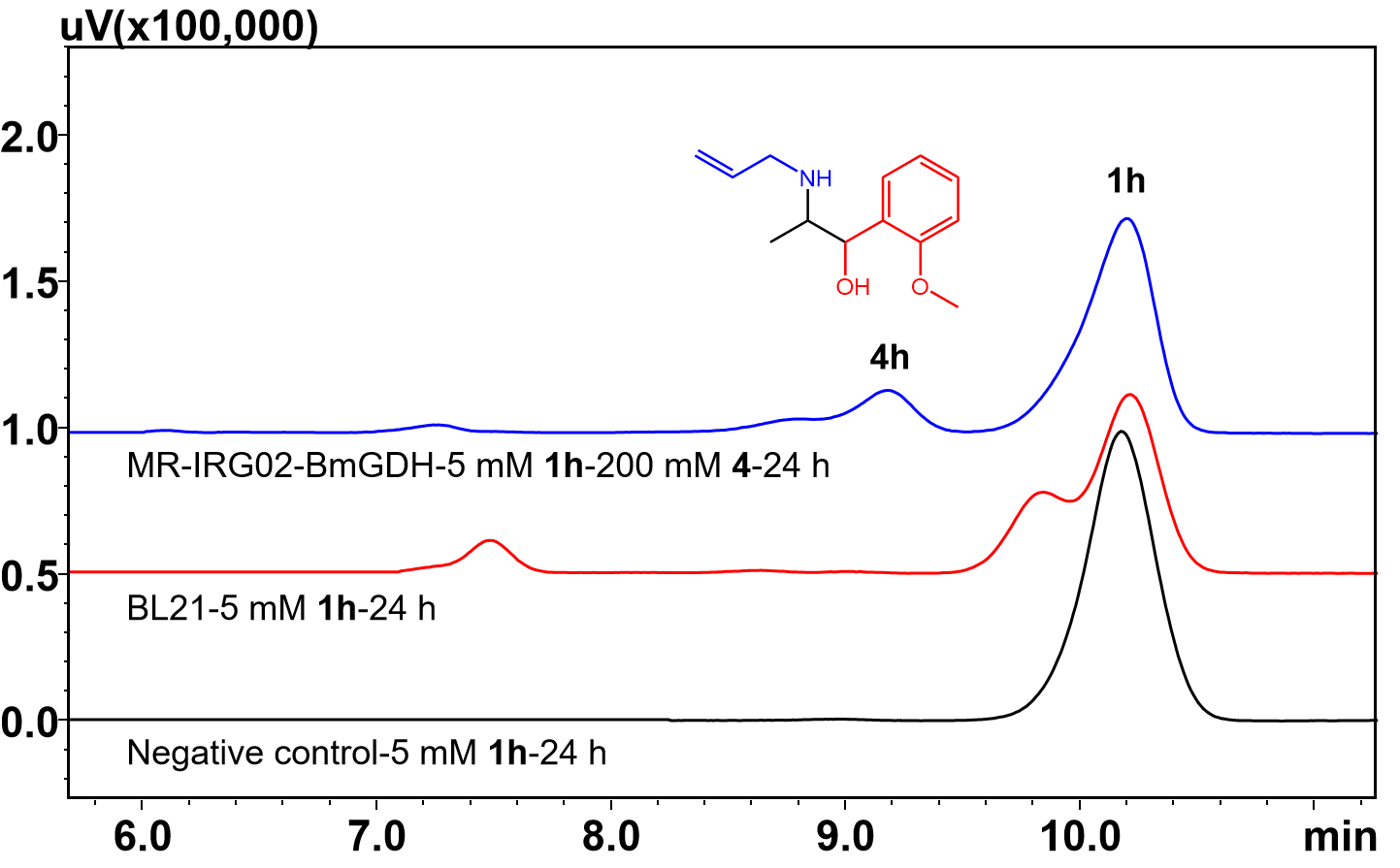
**

**Figure S11.3** HPLC analysis of MR-IRG02-*Bm*GDH catalyzed reductive amination of PAC analog **1h** with amine **4**. The negative control is the BL21 catalyzed reaction with possible by-product formation. The targeted product is **4h**.

**
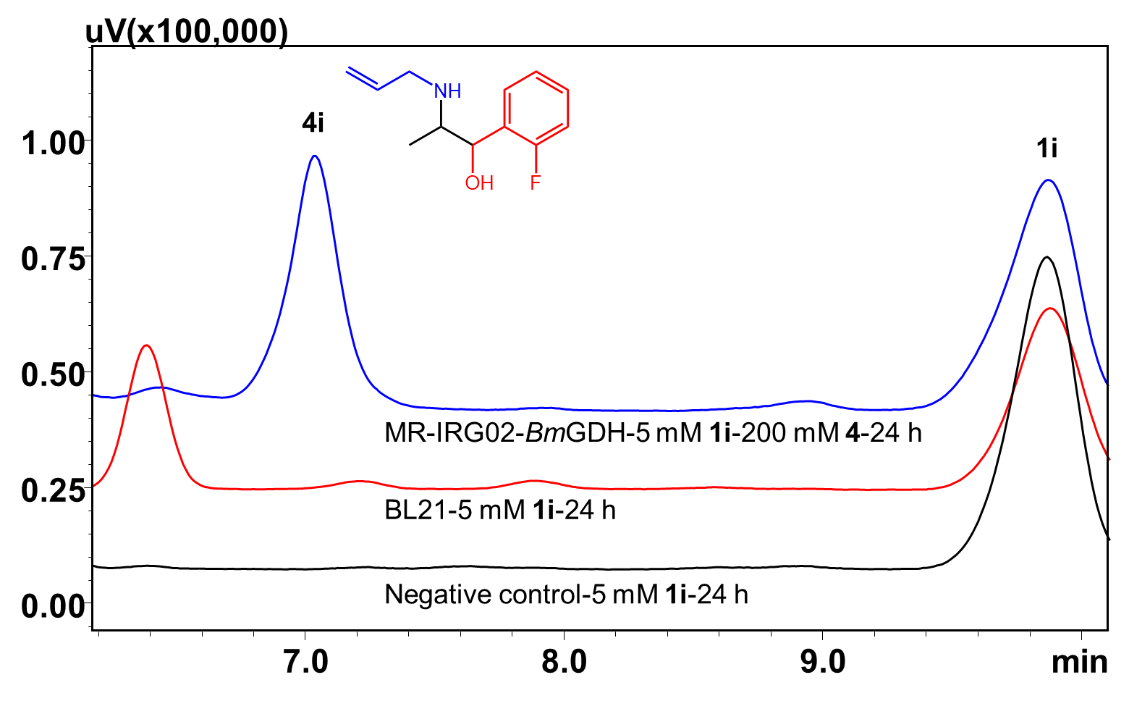
**

**Figure S11.4** HPLC analysis of MR-IRG02-*Bm*GDH catalyzed reductive amination of PAC analog **1i** with amine **4**. The negative control is the BL21 catalyzed reaction with possible by-product formation. The targeted product is **4i**.


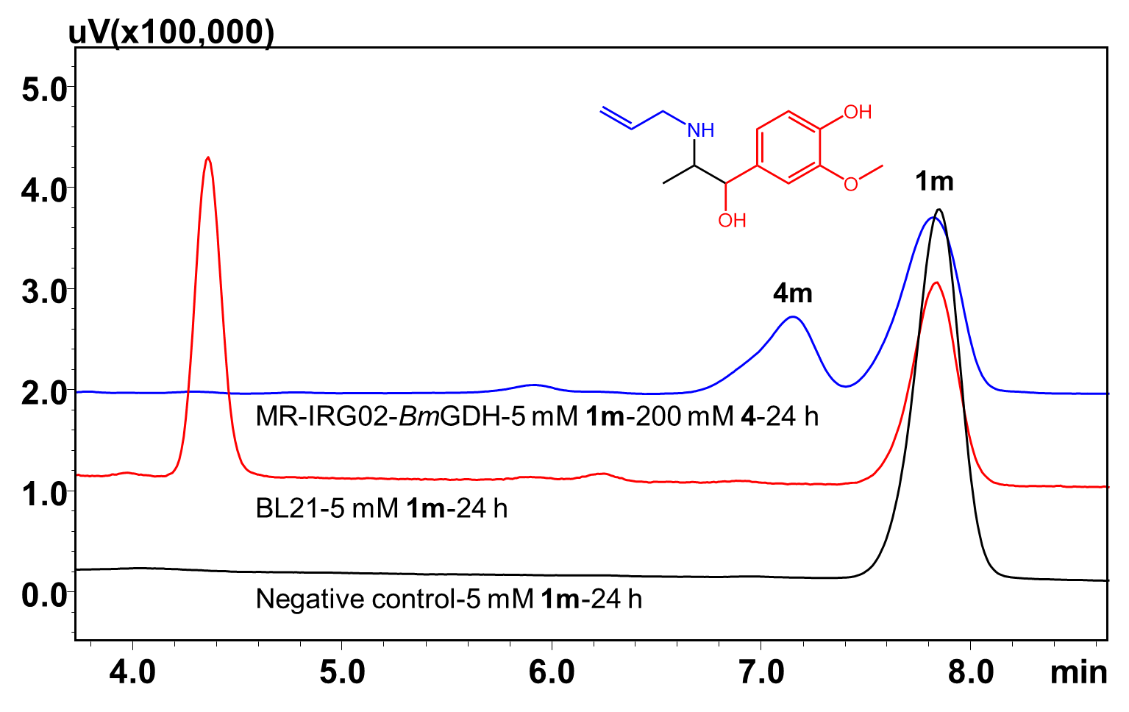


**Figure S11.5** HPLC analysis of MR-IRG02-*Bm*GDH catalyzed reductive amination of PAC analog **1m** with amine **4**. The negative control is the BL21 catalyzed reaction with possible by-product formation. The targeted product is **4m**.

**
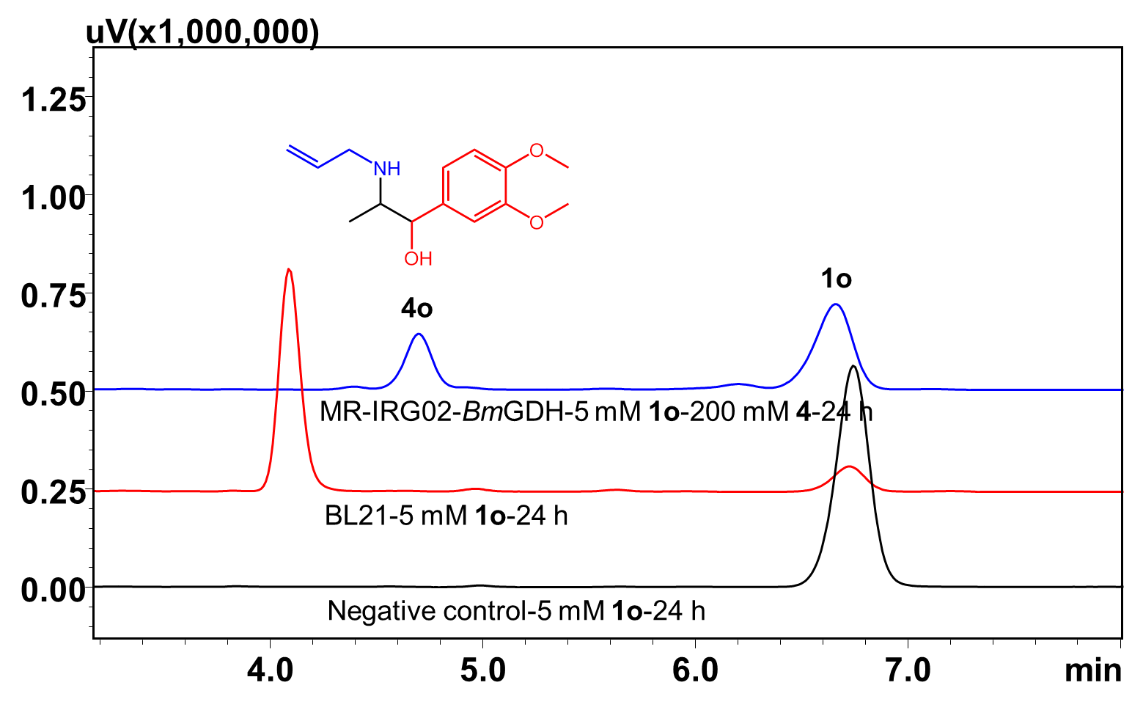
**

**Figure S11.6** HPLC analysis of MR-IRG02-*Bm*GDH catalyzed reductive amination of PAC analog **1o** with amine **4**. The negative control is the BL21 catalyzed reaction with possible by-product formation. The targeted product is **4o**.

**
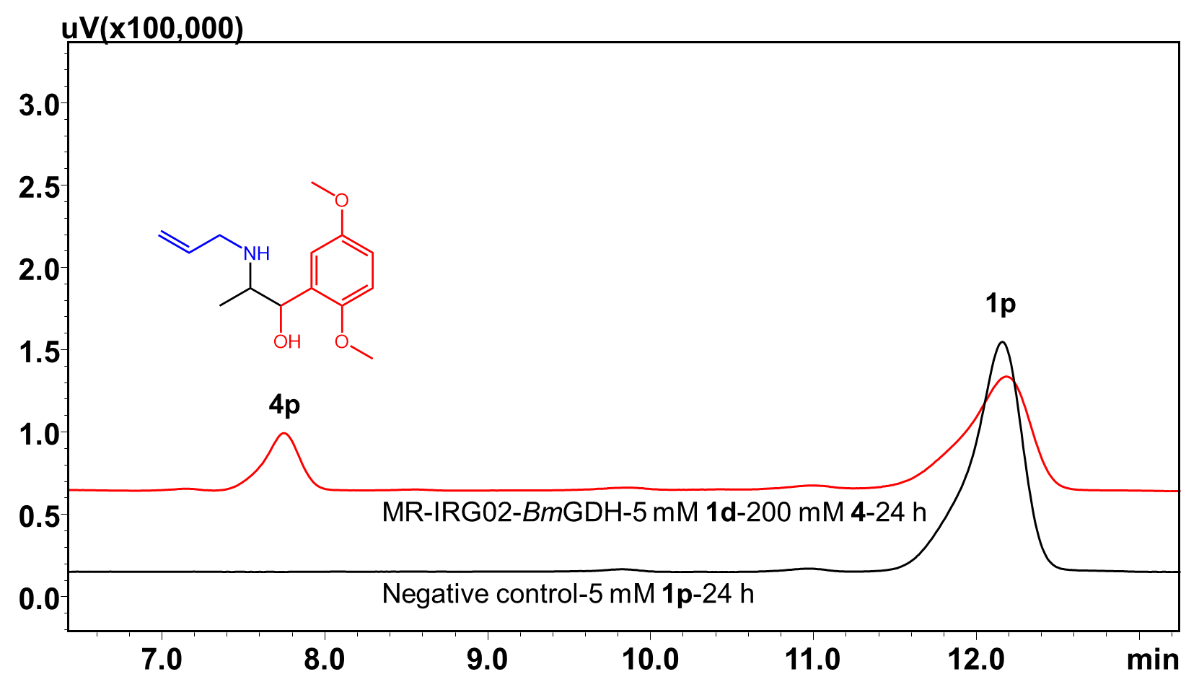
**

**Figure S11.7** HPLC analysis of MR-IRG02-*Bm*GDH catalyzed reductive amination of PAC analog **1p** with amine **4**. The negative control is the BL21 catalyzed reaction with possible by-product formation. The targeted product is **4p**.


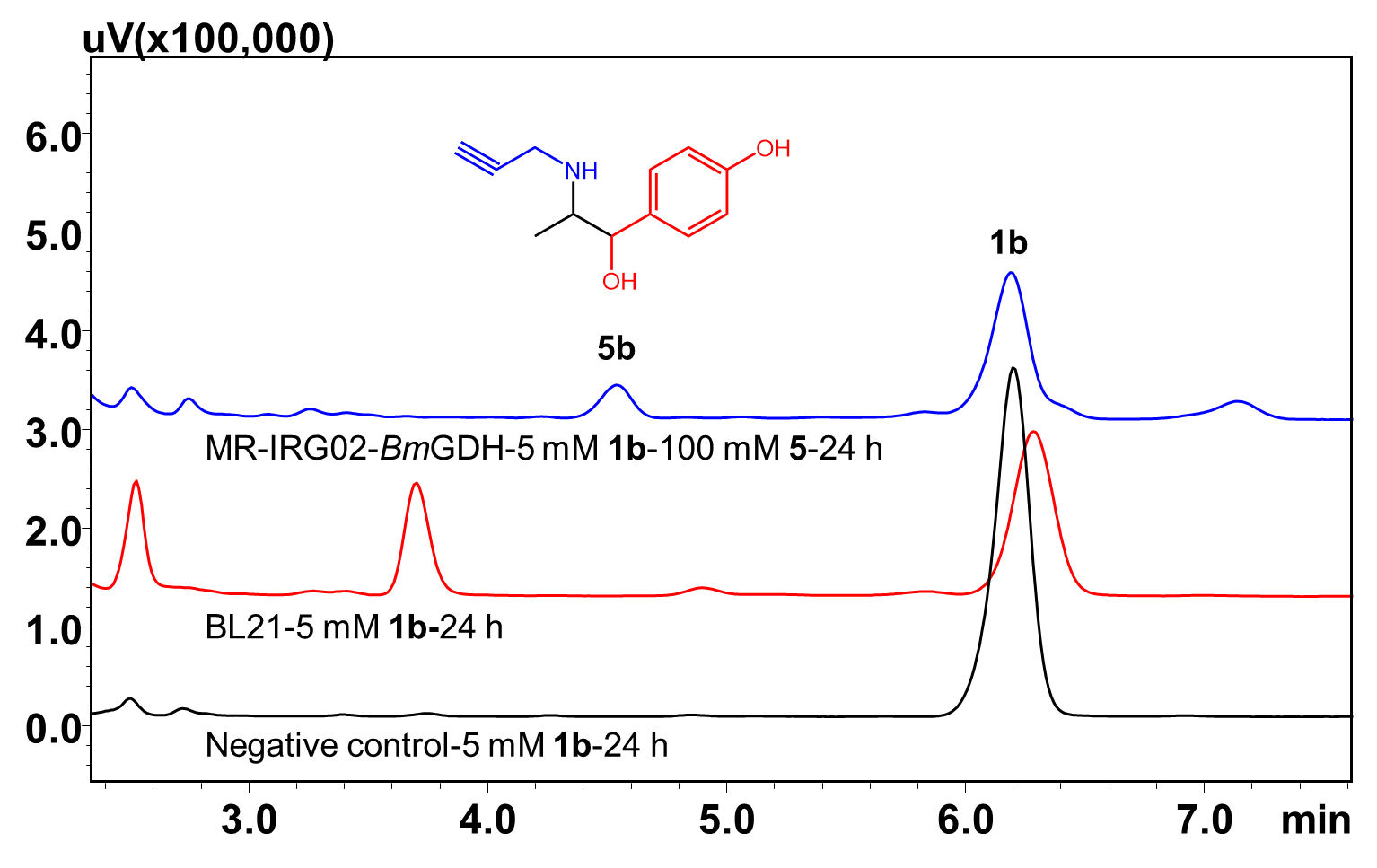


**Figure S11.8** HPLC analysis of MR-IRG02-*Bm*GDH catalyzed reductive amination of PAC analog **1b** with amine **5**. The negative control is the BL21 catalyzed reaction with possible by-product formation. The targeted product is **5b**.


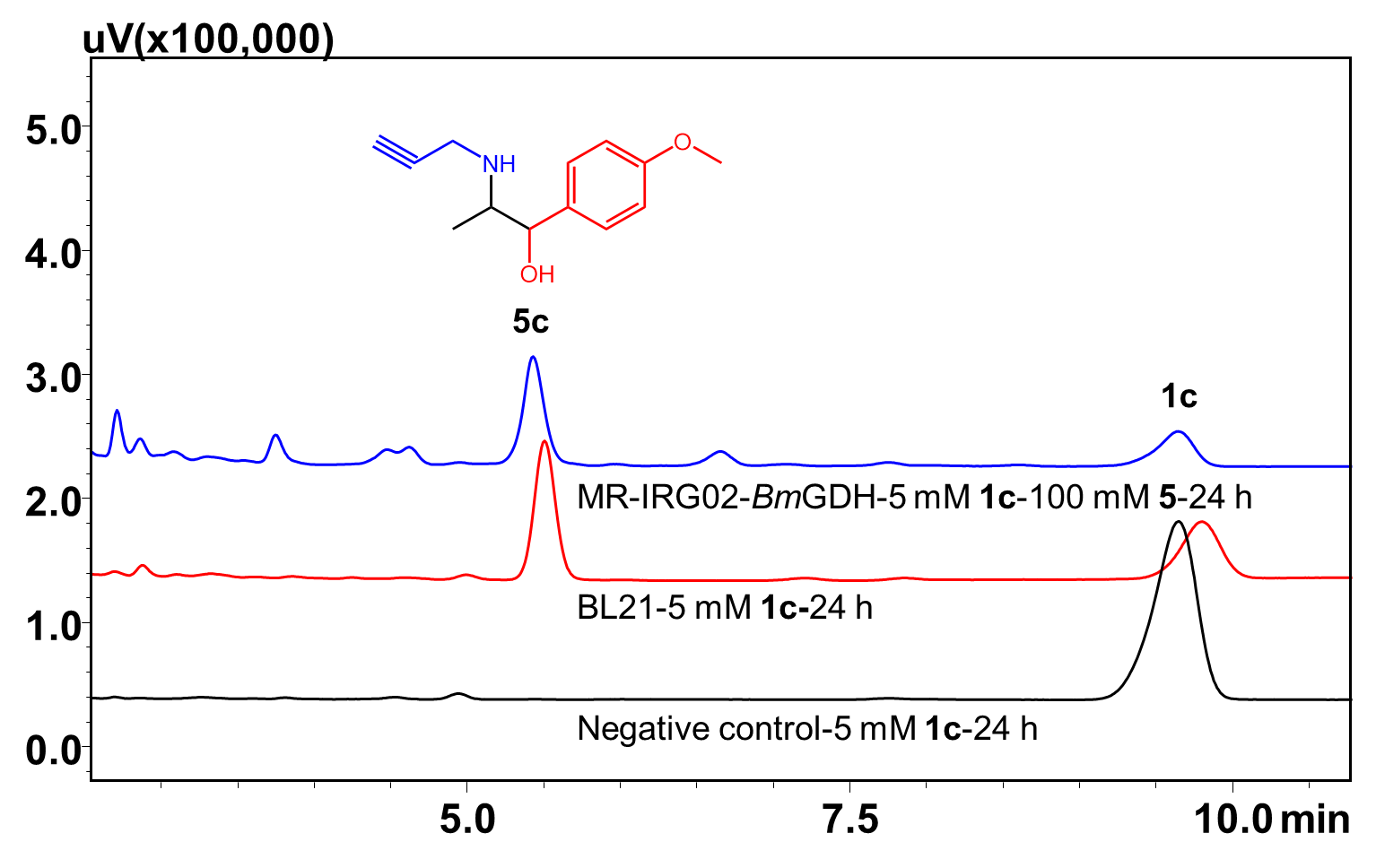


**Figure S11.9** HPLC analysis of MR-IRG02-*Bm*GDH catalyzed reductive amination of PAC analog **1c** with amine **5**. The negative control is the BL21 catalyzed reaction with possible by-product formation. The targeted product is **5c**.


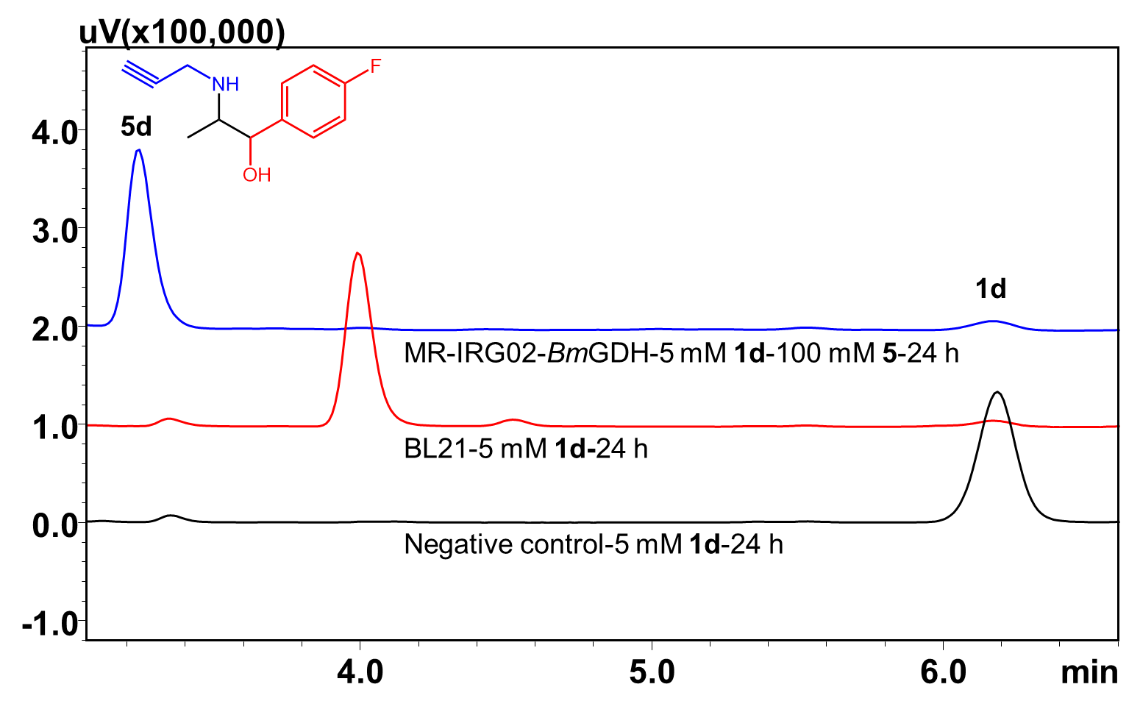


**Figure S11.10** HPLC analysis of MR-IRG02-*Bm*GDH catalyzed reductive amination of PAC analog **1d** with amine **5**. The negative control is the BL21 catalyzed reaction with possible by-product formation. The targeted product is **5d**.


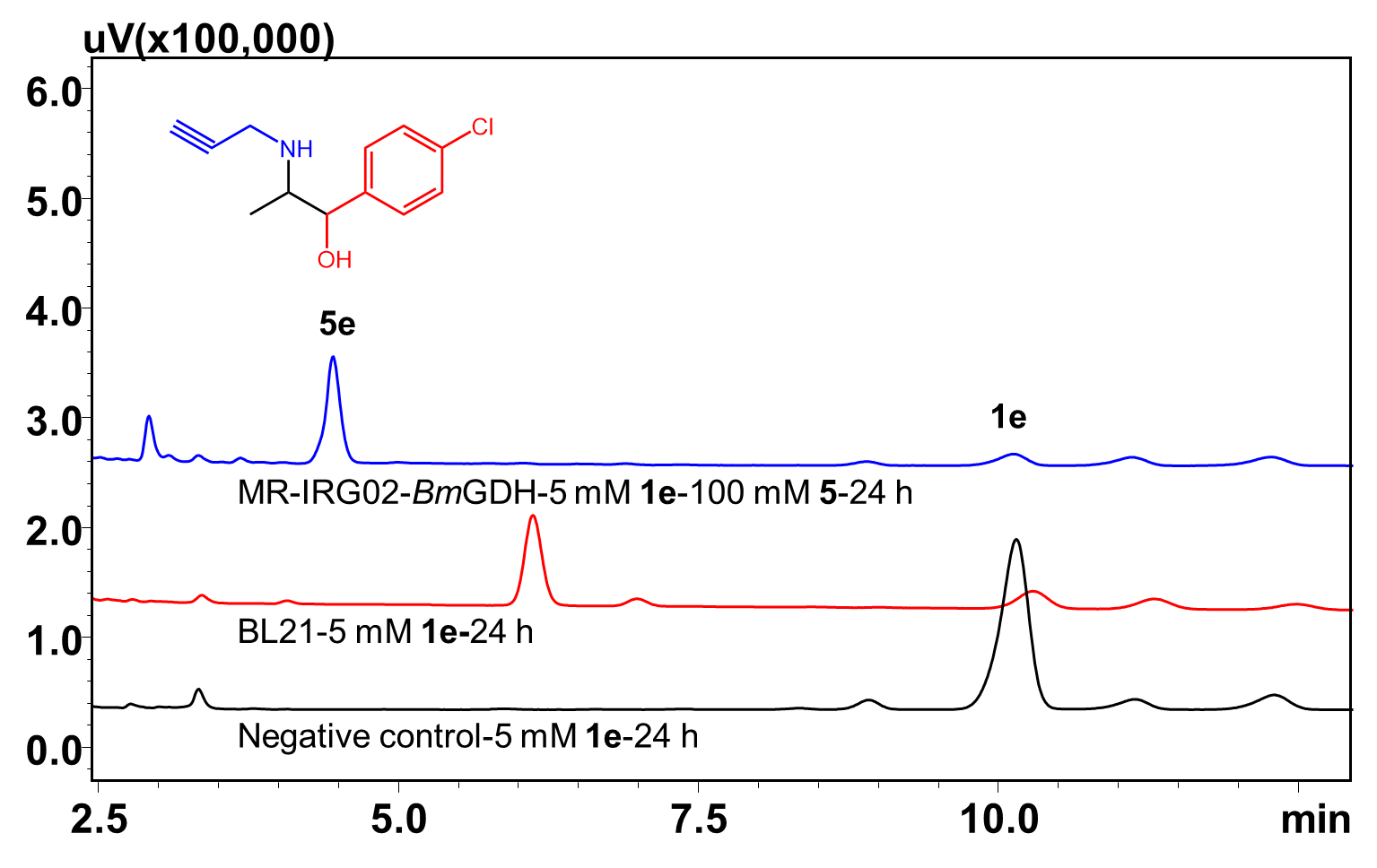


**Figure S11.11** HPLC analysis of MR-IRG02-*Bm*GDH catalyzed reductive amination of PAC analog **1e** with amine **5**. The negative control is the BL21 catalyzed reaction with possible by-product formation. The targeted is **5e**.


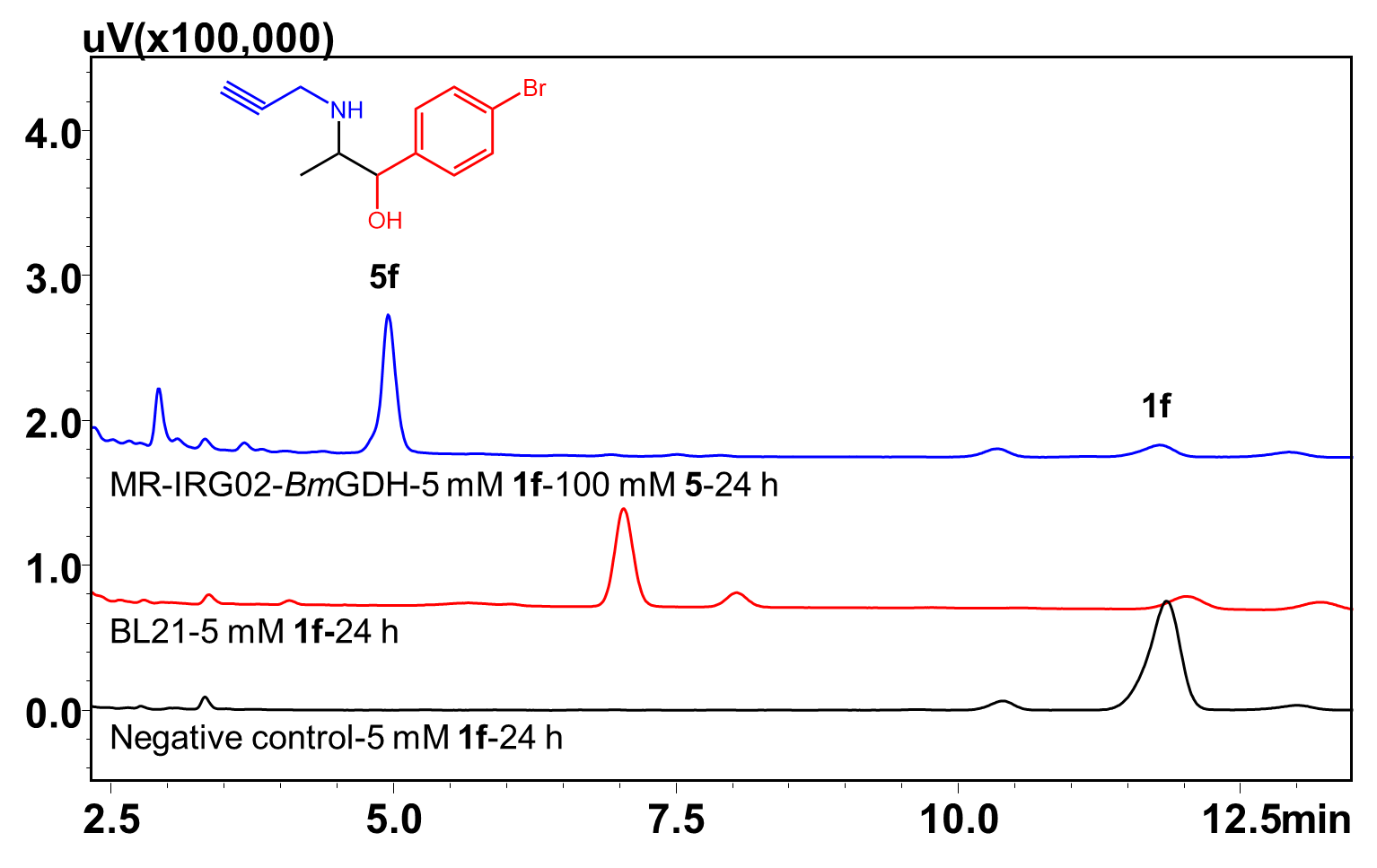


**Figure S11.12** HPLC analysis of MR-IRG02-*Bm*GDH catalyzed reductive amination of PAC analog **1f** with amine **5**. The negative control is the BL21 catalyzed reaction with possible by-product formation. The targeted product is **5f**.


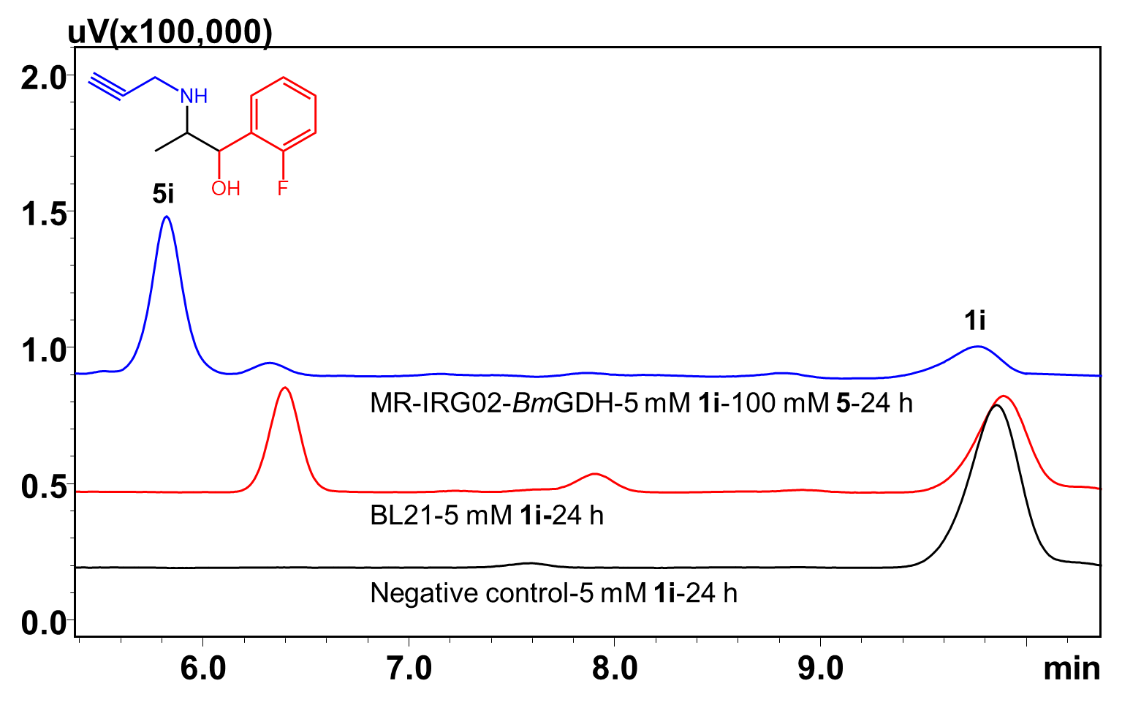


**Figure S11.13** HPLC analysis of MR-IRG02-*Bm*GDH catalyzed reductive amination of PAC analog **1i** with amine **5**. The negative control is the BL21 catalyzed reaction with possible by-product formation. The targeted product is **5i**.


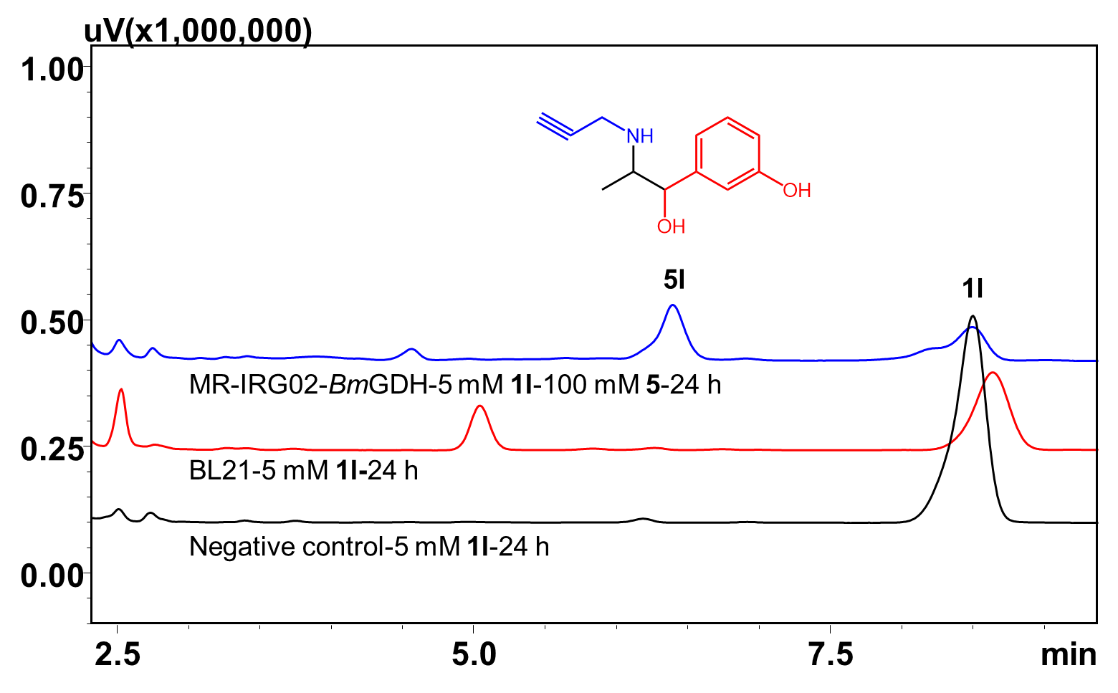


**Figure S11.14** HPLC analysis of MR-IRG02-*Bm*GDH catalyzed reductive amination of PAC analog **1l** with amine **5**. The negative control is the BL21 catalyzed reaction with possible by-product formation. The targeted product is **5l**.


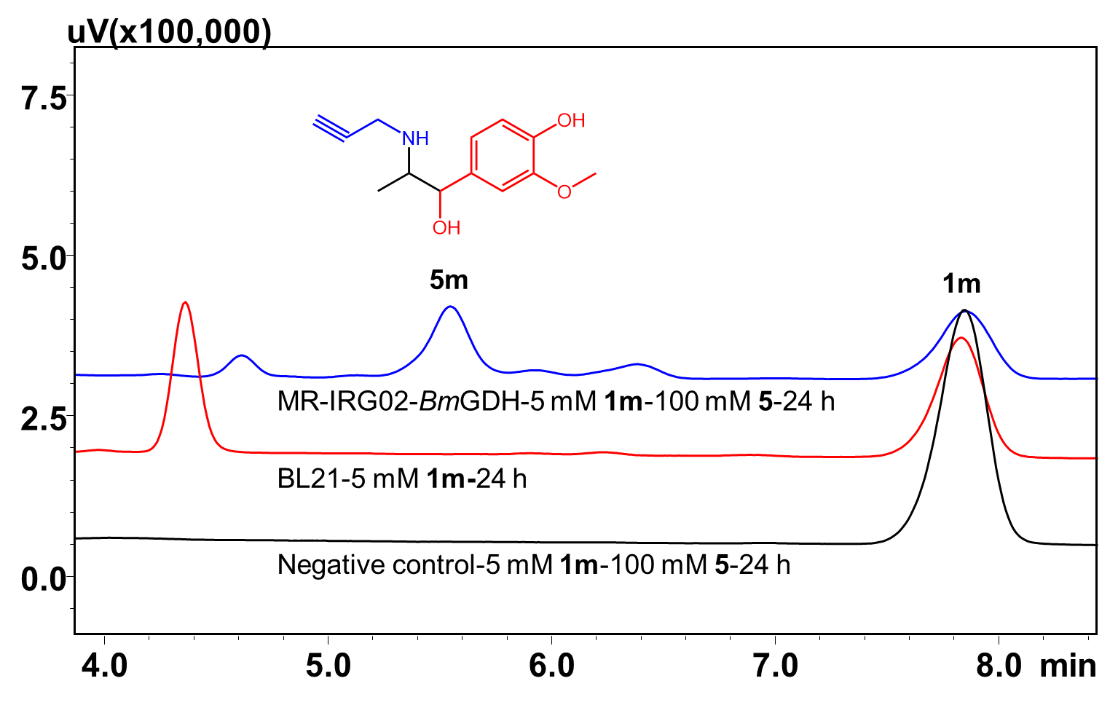


**Figure S11.15** HPLC analysis of MR-IRG02-*Bm*GDH catalyzed reductive amination of PAC analog **1m** with amine **5**. The negative control is the BL21 catalyzed reaction with possible by-product formation. The targeted product is **5m**.


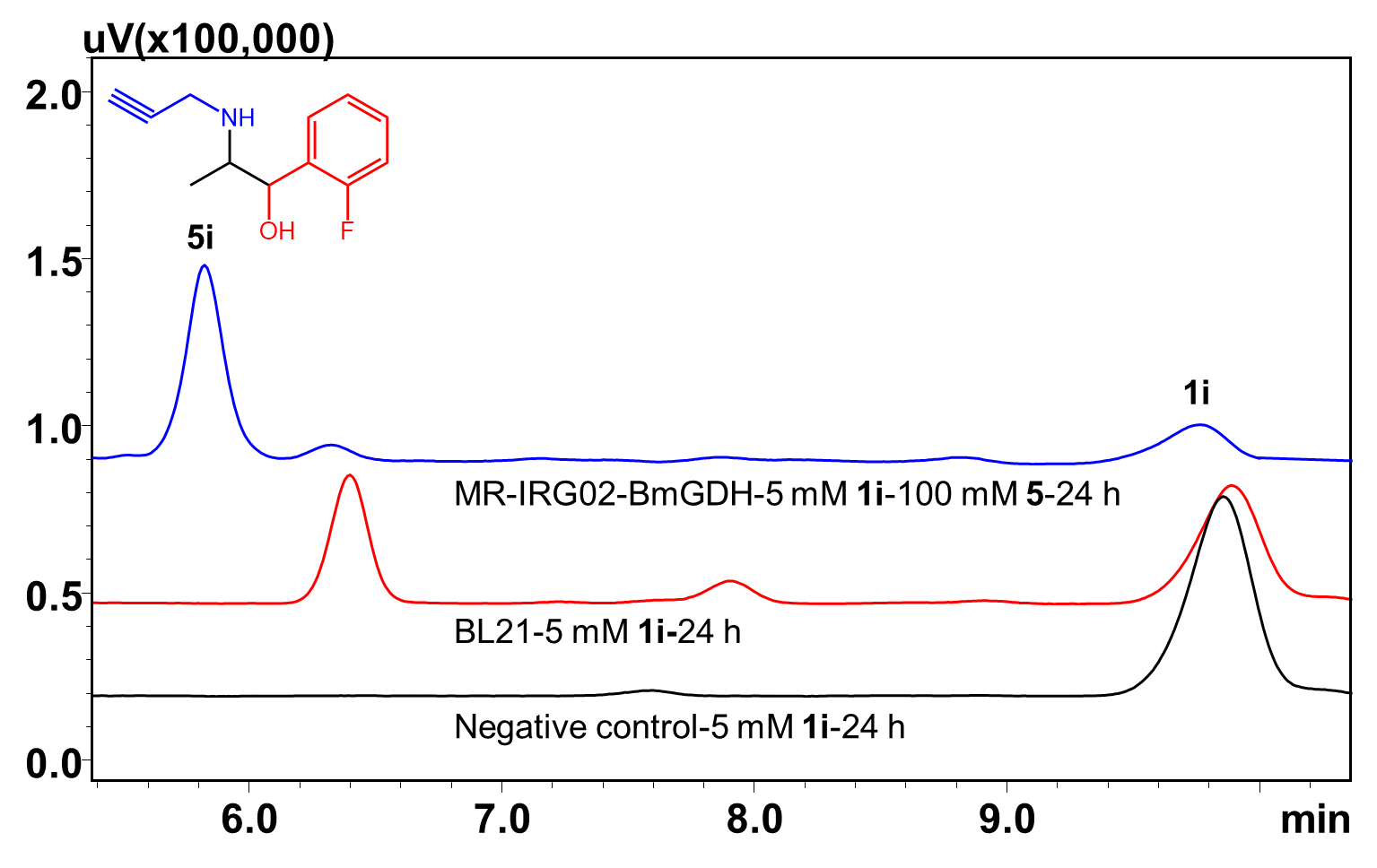


**Figure S11.16** HPLC analysis of MR-IRG02-*Bm*GDH catalyzed reductive amination of PAC analog **1i** with amine **5**. The negative control is the BL21 catalyzed reaction with possible by-product formation. The targeted product is **5i**.


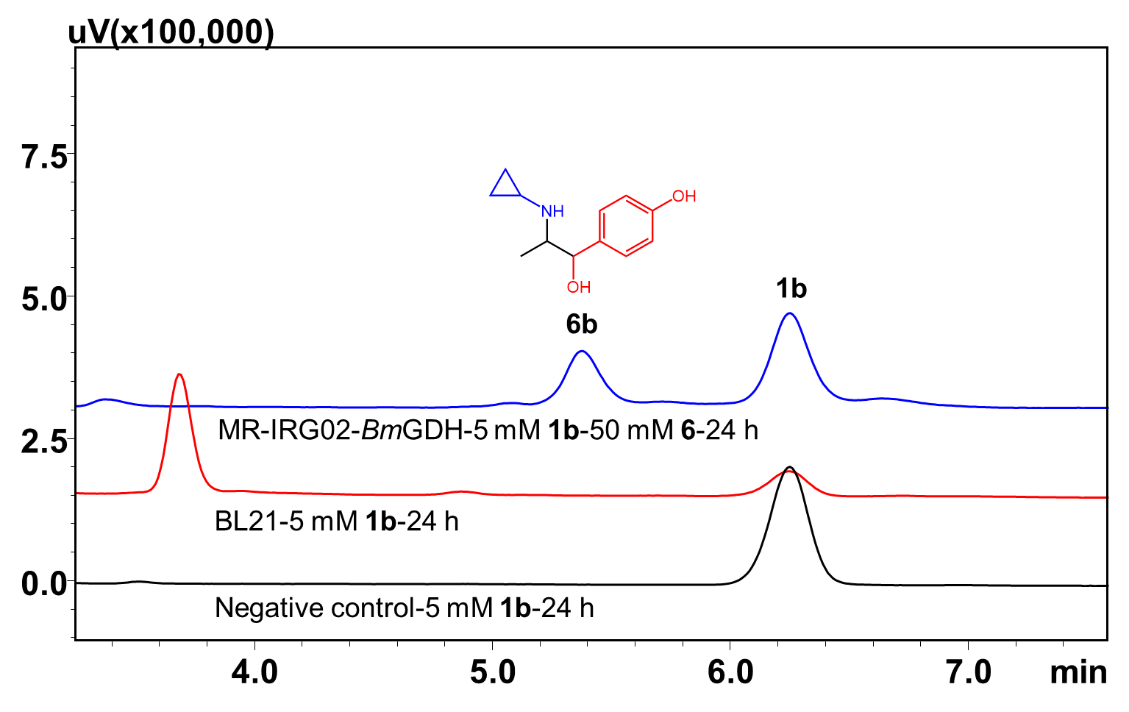


**Figure S11.17** HPLC analysis of MR-IRG02-*Bm*GDH catalyzed reductive amination of PAC analog **1b** with amine **6**. The negative control is the BL21 catalyzed reaction with possible by-product formation. The targeted product is **6b**.


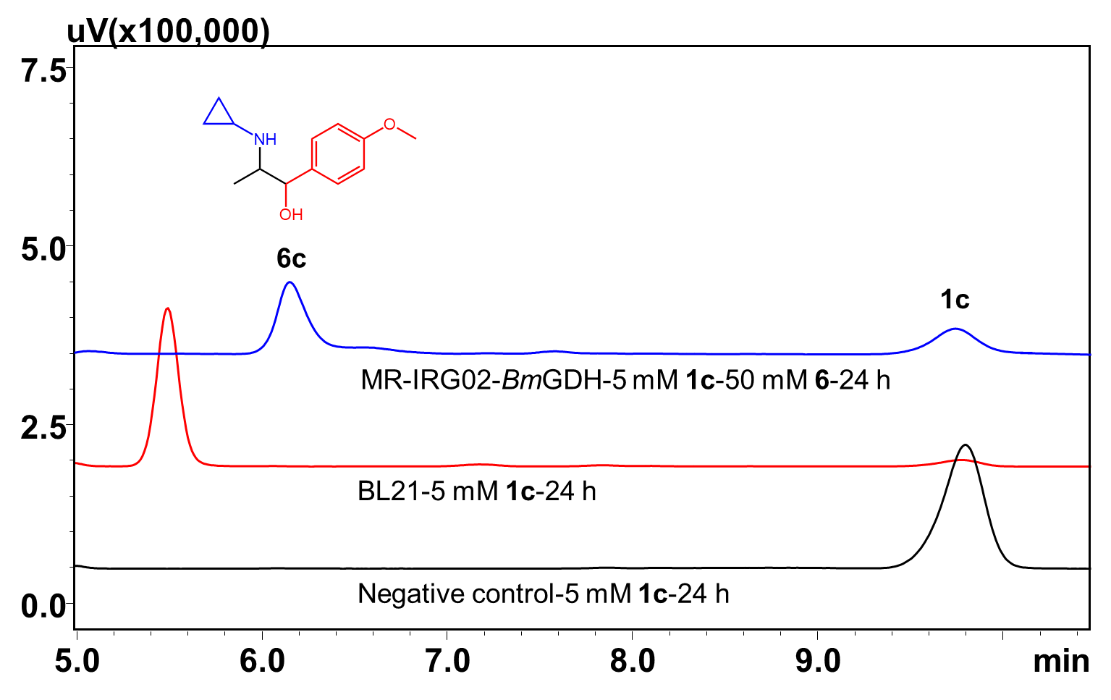


**Figure S11.18** HPLC analysis of MR-IRG02-*Bm*GDH catalyzed reductive amination of PAC analog **1c** with amine **6**. The negative control is the BL21 catalyzed reaction with possible by-product formation. The targeted product is **6c**.


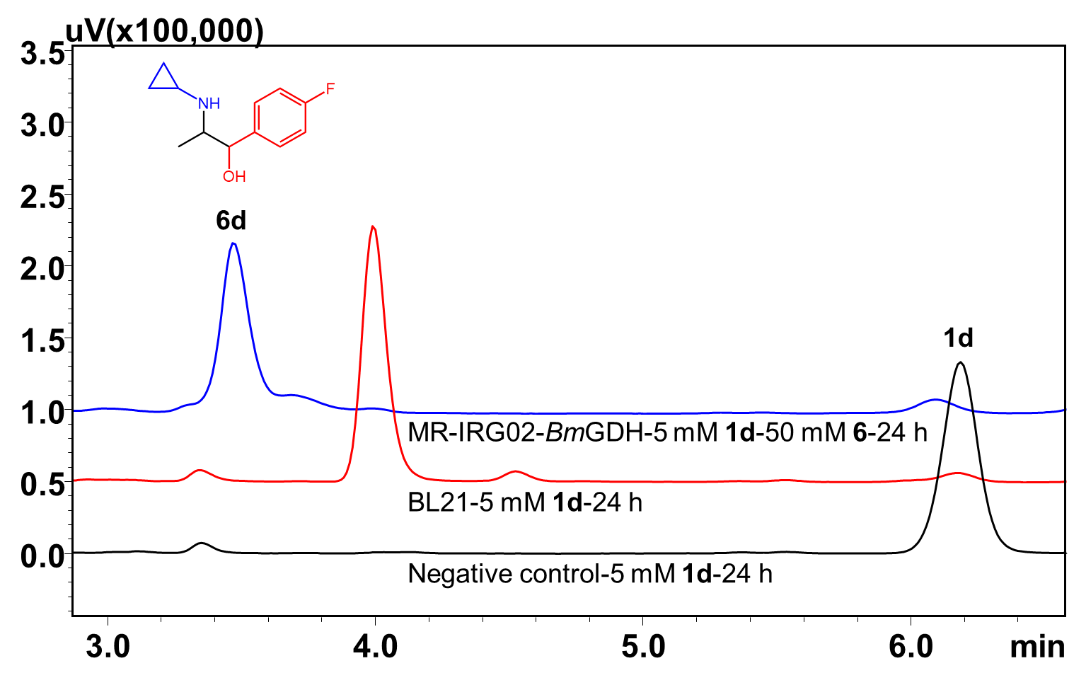


**Figure S11.19** HPLC analysis of MR-IRG02-*Bm*GDH catalyzed reductive amination of PAC analog **1d** with amine **6**. The negative control is the BL21 catalyzed reaction with possible by-product formation. The targeted product is **6d**.


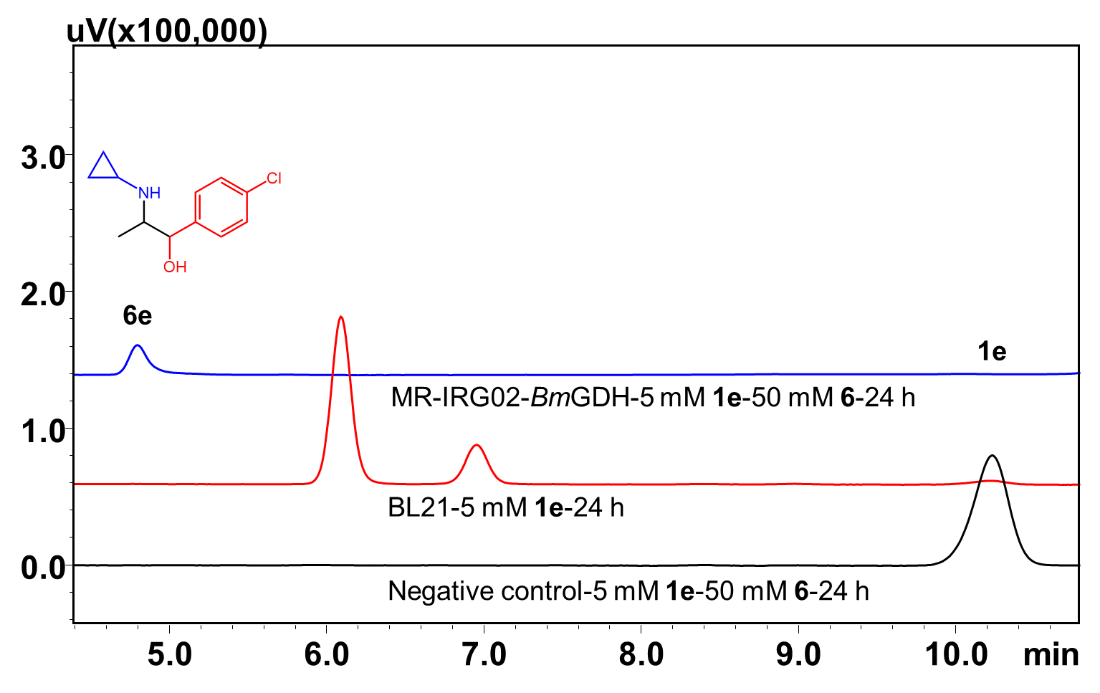


**Figure S11.20** HPLC analysis of MR-IRG02-*Bm*GDH catalyzed reductive amination of PAC analog **1e** with amine **6**. The negative control is the BL21 catalyzed reaction with possible by-product formation. The targeted product is **6e**.


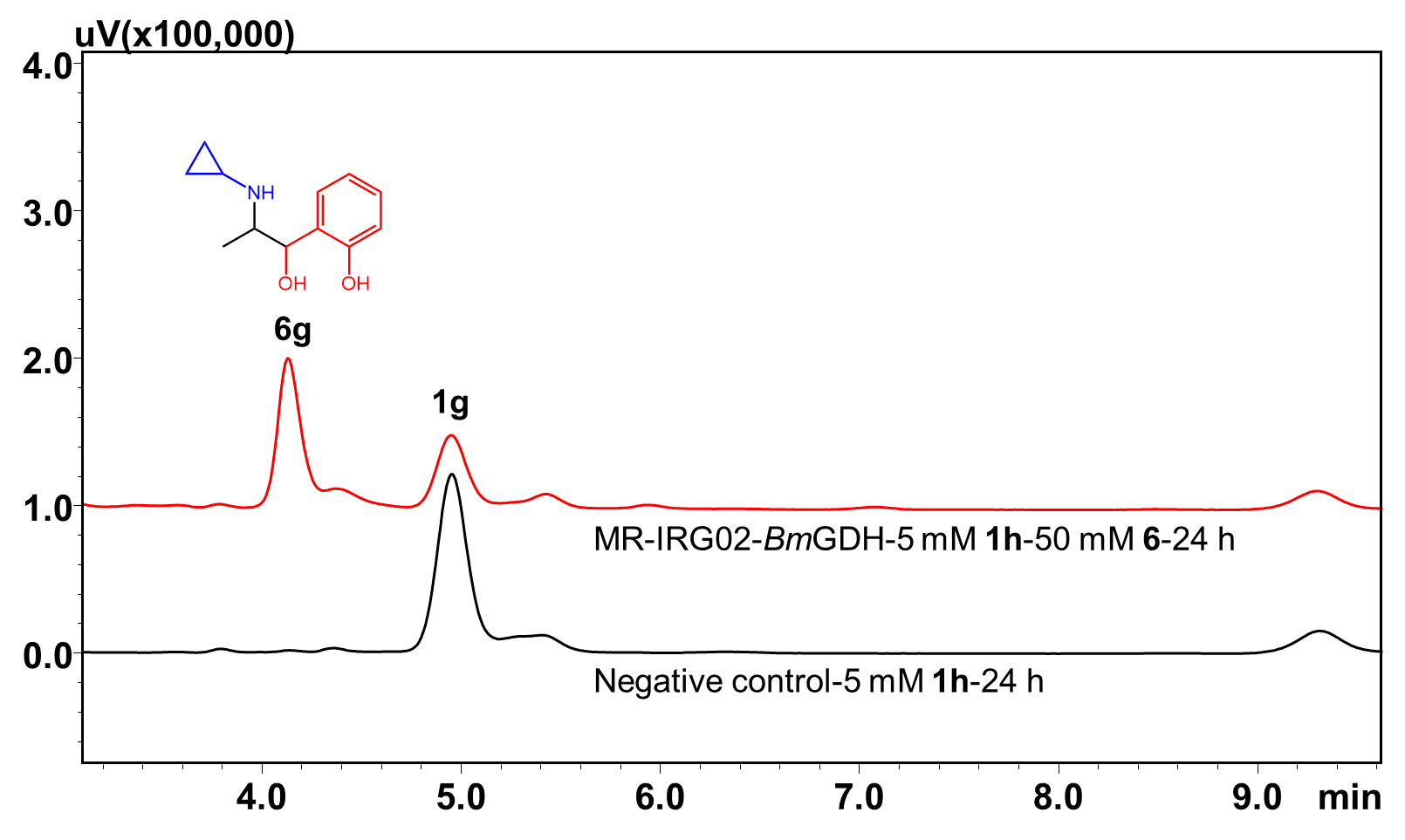


**Figure S11.21** HPLC analysis of MR-IRG02-*Bm*GDH catalyzed reductive amination of PAC analog **1g** with amine **6**. The negative control is the BL21 catalyzed reaction with possible by-product formation. The targeted product is **6g**.


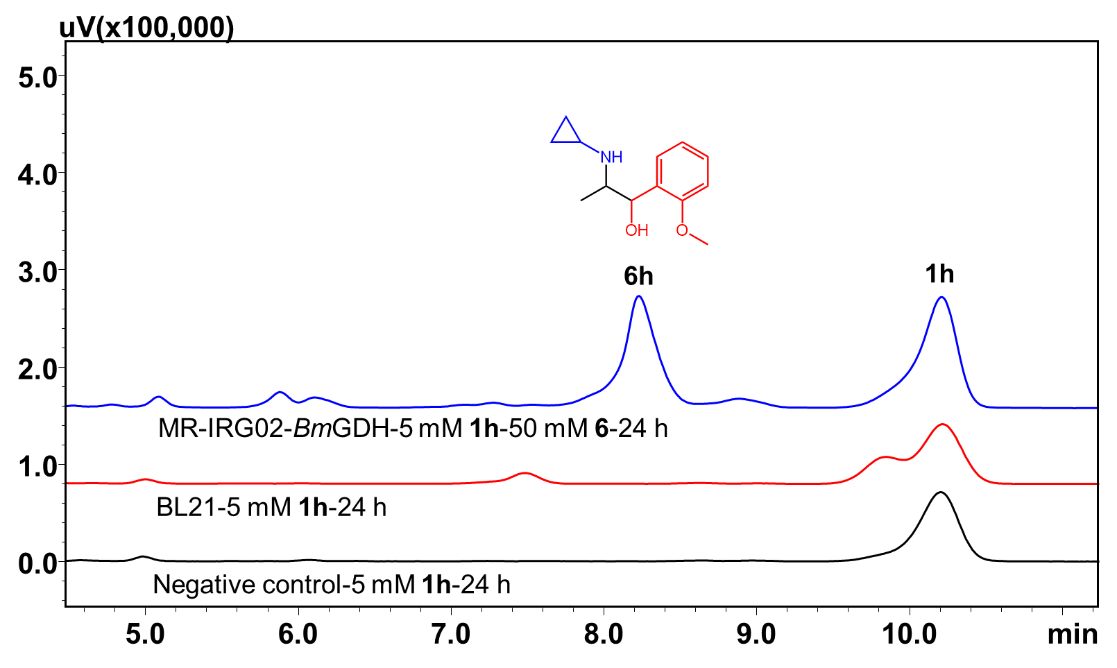


**Figure S11.22** HPLC analysis of MR-IRG02-*Bm*GDH catalyzed reductive amination of PAC analog **1h** with amine **6**. The negative control is the BL21 catalyzed reaction with possible by-product formation. The targeted product is **6h**.


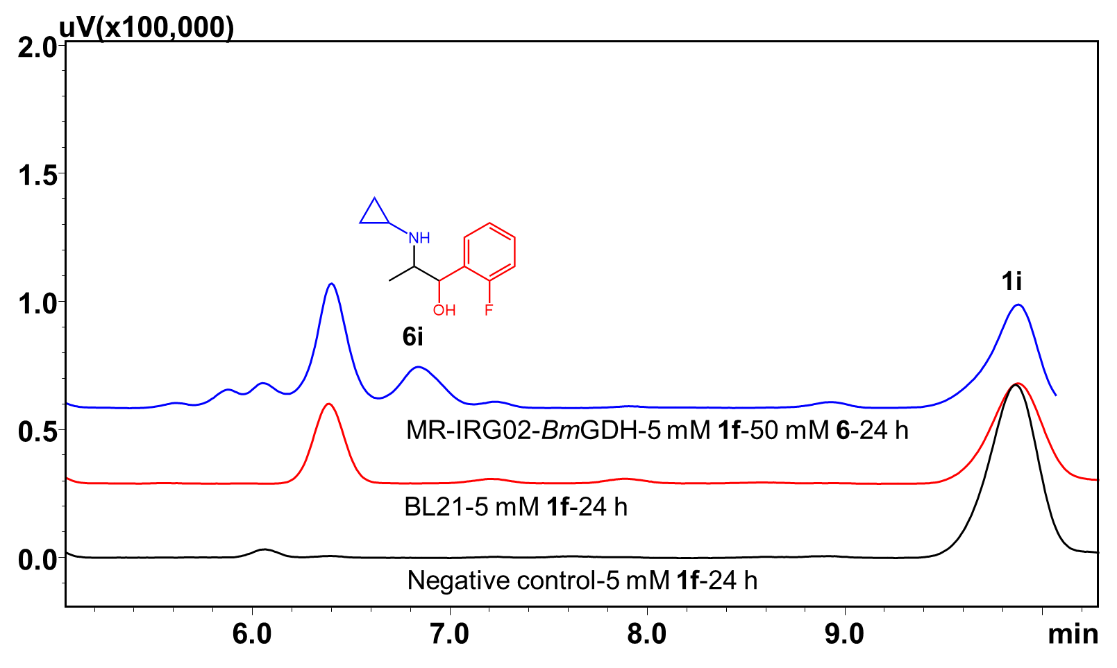


**Figure S11.23** HPLC analysis of MR-IRG02-*Bm*GDH catalyzed reductive amination of PAC analog **1i** with amine **6**. The negative control is the BL21 catalyzed reaction with possible by-product formation. The targeted product is **6i**.


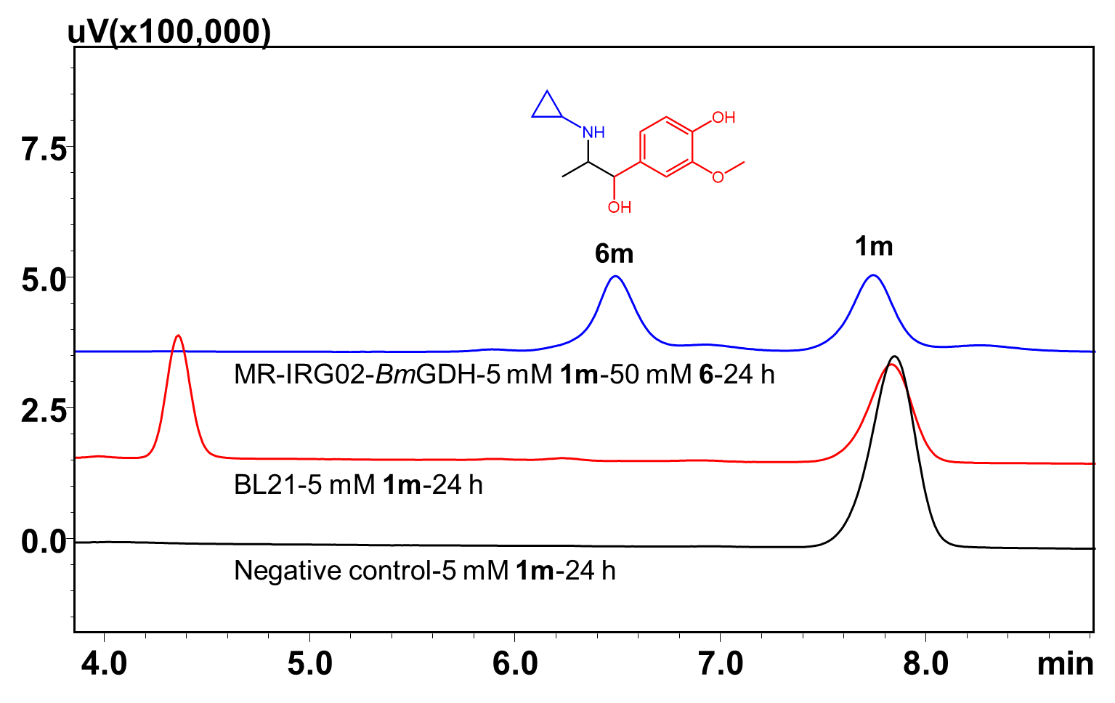


**Figure S11.24** HPLC analysis of MR-IRG02-*Bm*GDH catalyzed reductive amination of PAC analog **1m** with amine **6**. The negative control is the BL21 catalyzed reaction with possible by-product formation. The targeted product is **6m**.


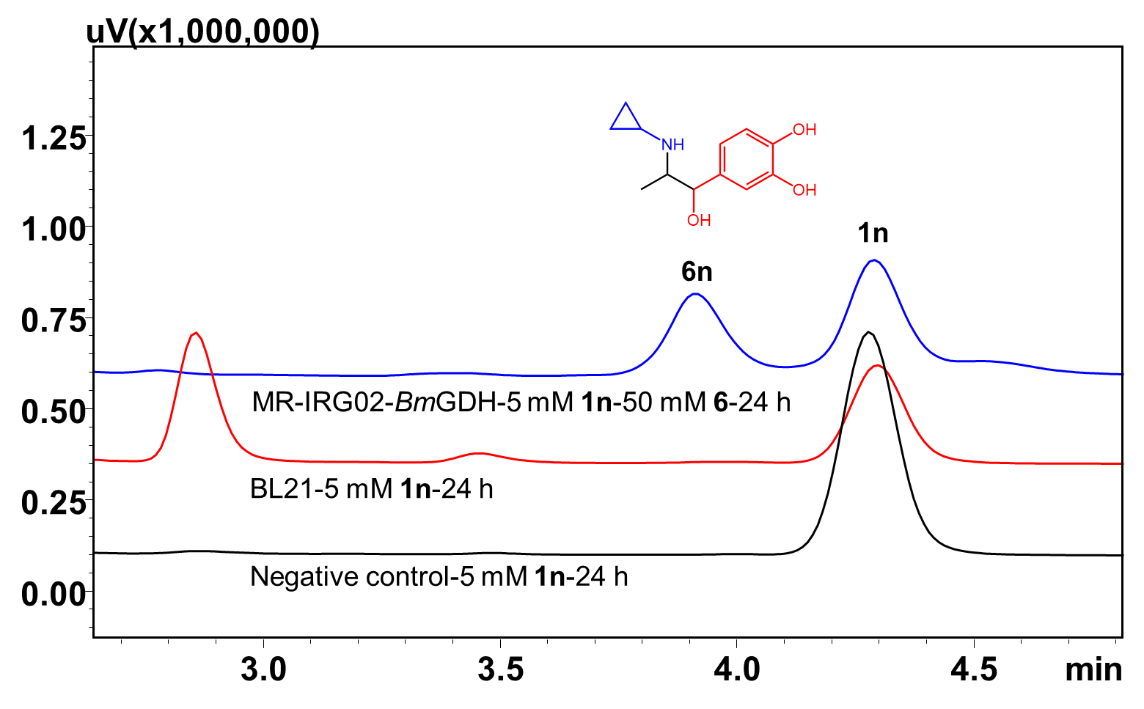


**Figure S11.25** HPLC analysis of MR-IRG02-*Bm*GDH catalyzed reductive amination of PAC analog **1n** with amine **6**. The negative control is the BL21 catalyzed reaction with possible by-product formation. The targeted d product is **6n**.


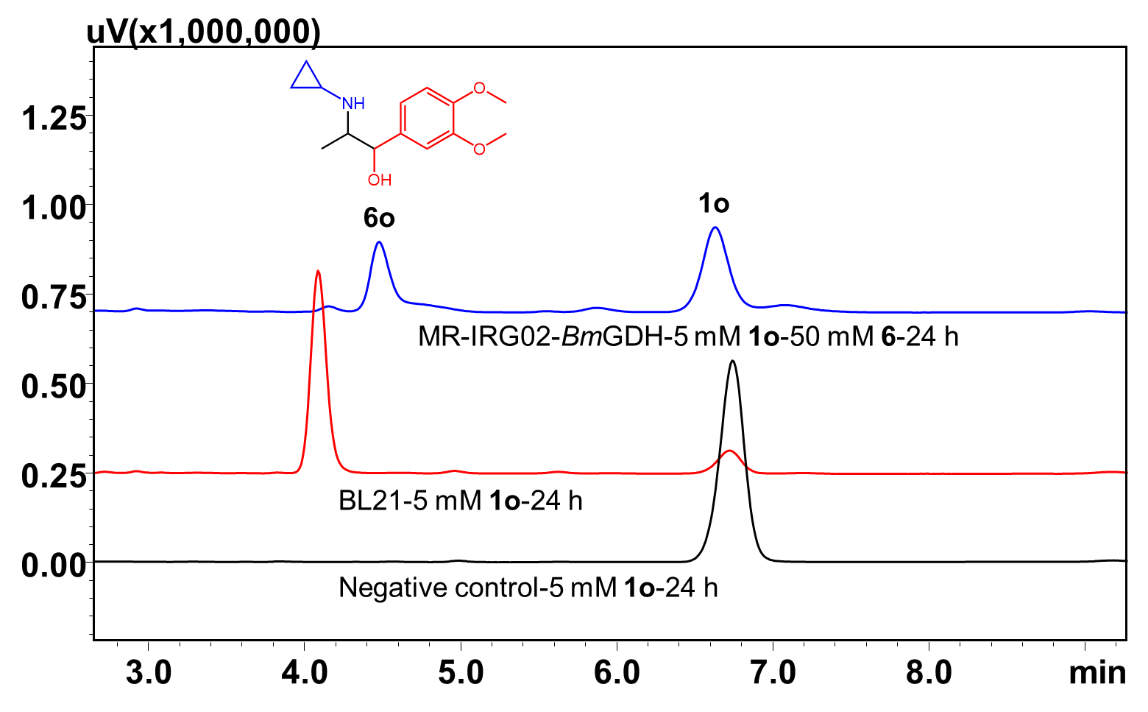


**Figure S11.26** HPLC analysis of MR-IRG02-*Bm*GDH catalyzed reductive amination of PAC analog **1o** with amine **6**. The negative control is the BL21 catalyzed reaction with possible by-product formation. The targeted product is **6o**.

**Figure S12.1** MS/MS spectrum of **1a**. The parent ion at m/z 151.0951 represents the [M+H]^+^ peak of **1a** (calcd m/z 151.0759, C_9_H_11_O_2_).

**Figure S12.2** MS/MS spectrum of **1b**. The parent ion at m/z 165.0603 represents the [M-H]^-^ peak of **1b** (calcd m/z 165.0552, C_9_H_10_O_3_).

**Figure S12.3** MS/MS spectrum of **1c**. The parent ion at m/z 181.1104 represents the [M+H]^+^ peak of **1c** (calcd m/z 181.0865, C_10_H_12_O_3_).

**Figure S12.4** MS/MS spectrum of **1d**. The parent ion at m/z 169.0635 represents the [M+H] ^+^ peak of **1d** (calcd m/z 169.0665, C_9_H_9_FO_2_).

**Figure S12.5** MS/MS spectrum of **1e**. The parent ion at m/z 185.1308 represents the [M+H]^+^ peak of **1e** (calcd m/z 185.0369, C_9_H_9_ClO_2_).

**Figure S12.6** MS/MS spectrum of **1f**. The parent ion at m/z 228.9566 represents the [M+H]^+^ peak of **1f** (calcd m/z 228.9864, C_9_H_9_BrO_2_).

**Figure S12.7** MS/MS spectrum of **1g**. The parent ion at m/z 165.0590 represents the [M-H]^-^ peak of **1g** (calcd m/z 165.0552, C_9_H_10_O_3_).

**Figure S12.8** MS/MS spectrum of **1h**. The parent ion at m/z 181.1061 represents the [M+H]^+^ peak of **1h** (calcd m/z 181.0865, C_10_H_12_O_3_).

**Figure S12.9** MS/MS spectrum of **1i**. The parent ion at m/z 169.0639 represents the [M+H] ^+^ peak of **1i** (calcd m/z 169.0665, C_9_H_9_FO_2_).

**Figure S12.10** MS/MS spectrum of **1j**. The parent ion at m/z 185.1295 represents the [M+H]^+^ peak of **1j** (calcd m/z 185.0369, C_9_H_9_ClO_2_).

**Figure S12.11** MS/MS spectrum of **1k**. The parent ion at m/z 229.1386 represents the [M+H]^+^ peak of compound **1k** (calcd m/z 228.9864, C_9_H_9_BrO_2_).

**Figure S12.12** MS/MS spectrum of **1l**. The parent ion at m/z 167.1203 represents the [M-H]^-^ peak of **1l** (calcd m/z 167.0708, C_9_H_10_O_3_).

**Figure S12.13** MS/MS spectrum of **1m**. The parent ion at m/z 195.0647 represents the [M-H] ^-^ peak of **1m** (calcd m/z 195.0657, C_10_H_12_O_4_).

**Figure S12.14** MS/MS spectrum of **1n**. The parent ion at m/z 181.0546 represents the [M-H]^-^ peak of **1n** (calcd m/z 181.0501, C_9_H_10_O_4_).

**Figure S12.15** MS/MS spectrum of **1o**. The parent ion at m/z 210.9797 represents the [M+H]^+^ peak of **1o** (calcd m/z 211.0970, C_11_H_14_O_4_).


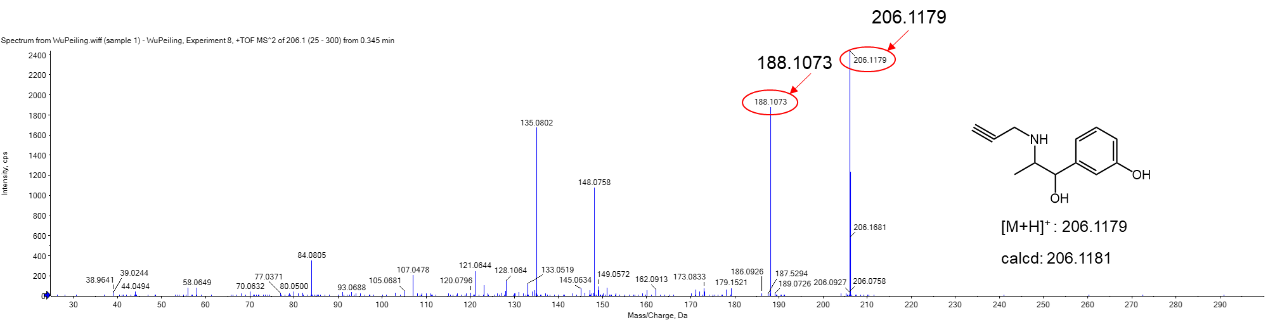


**Figure S12.16** MS/MS spectrum of **5l**. The parent ion at m/z 206.1179 represents the [M+H]^+^ peak of **5l** (calcd m/z 206.1181, C_12_H_15_NO_2_).


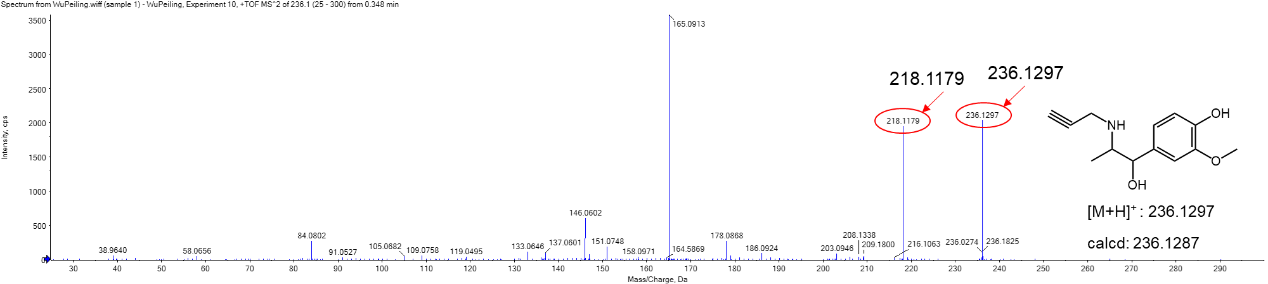


**Figure S12.17** MS/MS spectrum of **5m**. The parent ion at m/z 236.1297 represents the [M+H]^+^ peak of **5m** (calcd m/z 236.1287, C_13_H_17_NO_3_).


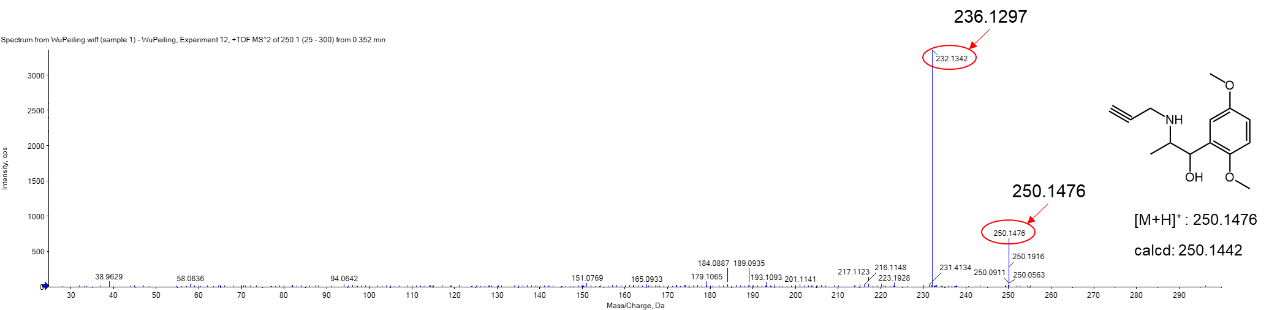


**Figure S12.18** MS/MS spectrum of **5o**. The parent ion at m/z 250.1476 represents the [M+H]^+^ peak of **5o** (calcd m/z 250.1442, C_14_H_19_NO_3_).


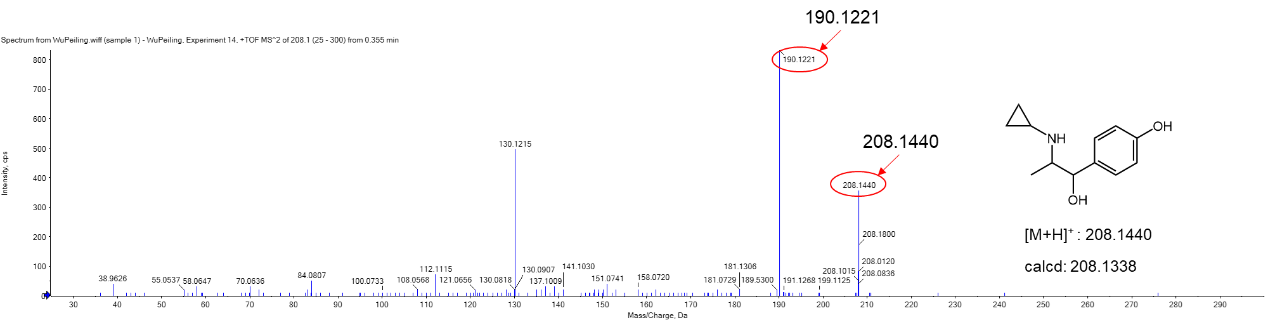


**Figure S12.19** MS/MS spectrum of **6b**. The parent ion at m/z 208.1440 represents the [M+H]^+^ peak of **6b** (calcd m/z 208.1338, C_12_H_17_NO_2_).


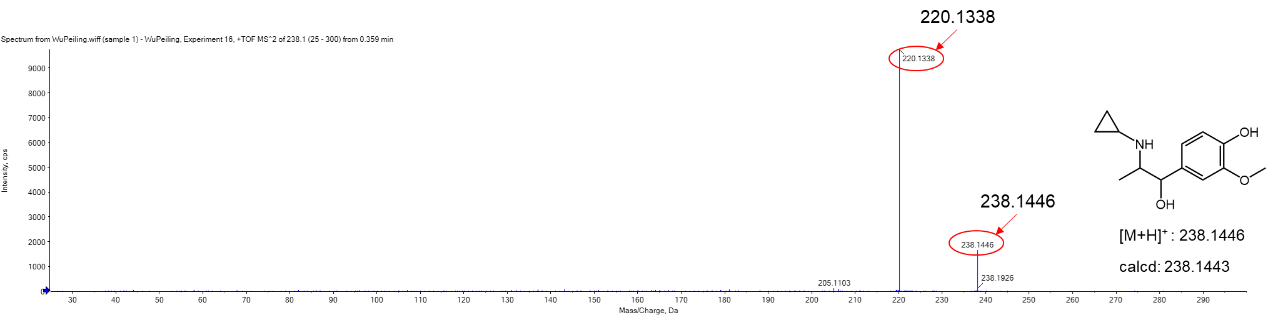


**Figure S12.20** MS/MS spectrum of **6m**. The parent ion at m/z 238.1446 represents the [M+H]^+^ peak of **6m** (calcd m/z 228.1443, C_13_H_19_NO_3_).


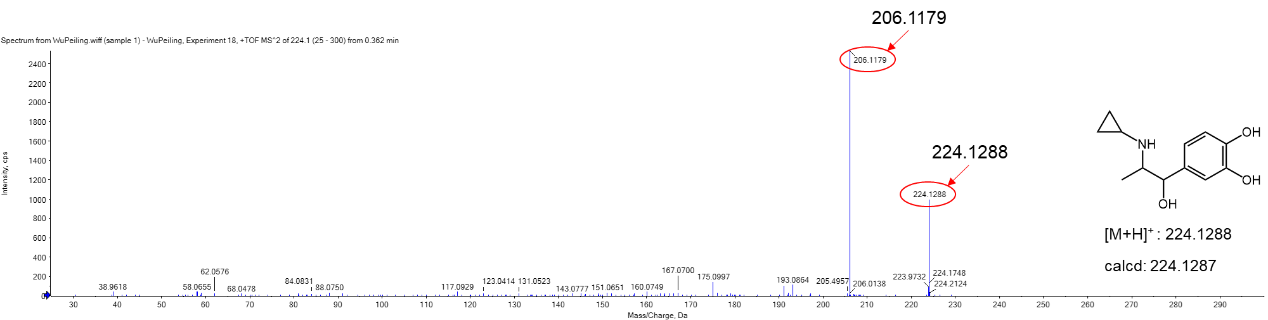


**Figure S12.21** MS/MS spectrum of **6n**. The parent ion at m/z 224.1288 represents the [M+H]^+^ peak of **6n** (calcd m/z 224.1287, C_12_H_17_NO_3_).

**References**

1. Zhang, J., Li, X., Chen, R., Tan, X., Liu, X., Ma, Y., Zhu, F., An, C., Wei, G., Yao, Y., et al. (2022). Actinomycetes-derived imine reductases with a preference towards bulky amine substrates. Communications Chemistry *5*, 123. <https://doi.org/10.1038/s42004-022-00743-y>.

2. Aleku, G.A., France, S.P., Man, H., Mangas-Sanchez, J., Montgomery, S.L., Sharma, M., Leipold, F., Hussain, S., Grogan, G., and Turner, N.J. (2017). A reductive aminase from Aspergillus oryzae. Nature Chemistry *9*, 961-969. <https://doi.org/10.1038/nchem.2782>.

3. Gilio, A.K., Thorpe, T.W., Heyam, A., Petchey, M.R., Pogrányi, B., France, S.P., Howard, R.M., Karmilowicz, M.J., Lewis, R., Turner, N., and Grogan, G. (2023). A Reductive Aminase Switches to Imine Reductase Mode for a Bulky Amine Substrate. ACS Catalysis *13*, 1669-1677. <https://doi.org/10.1021/acscatal.2c06066>.
